# DNA methylation profiles of diverse *Brachypodium distachyon* align with underlying genetic diversity

**DOI:** 10.1101/gr.205468.116

**Published:** 2016-11

**Authors:** Steven R. Eichten, Tim Stuart, Akanksha Srivastava, Ryan Lister, Justin O. Borevitz

**Affiliations:** 1 ARC Centre of Excellence in Plant Energy Biology, The Australian National University, Canberra, Australia, 2601;; 2 ARC Centre of Excellence in Plant Energy Biology, University of Western Australia, Perth, Australia, 6009

## Abstract

DNA methylation, a common modification of genomic DNA, is known to influence the expression of transposable elements as well as some genes. Although commonly viewed as an epigenetic mark, evidence has shown that underlying genetic variation, such as transposable element polymorphisms, often associate with differential DNA methylation states. To investigate the role of DNA methylation variation, transposable element polymorphism, and genomic diversity, whole-genome bisulfite sequencing was performed on genetically diverse lines of the model cereal *Brachypodium distachyon*. Although DNA methylation profiles are broadly similar, thousands of differentially methylated regions are observed between lines. An analysis of novel transposable element indel variation highlighted hundreds of new polymorphisms not seen in the reference sequence. DNA methylation and transposable element variation is correlated with the genome-wide amount of genetic variation present between samples. However, there was minimal evidence that novel transposon insertions or deletions are associated with nearby differential methylation. This study highlights unique relationships between genetic variation and DNA methylation variation within *Brachypodium* and provides a valuable map of DNA methylation across diverse resequenced accessions of this model cereal species.

Individuals of a species are often classified based on genetic variation found among them. In addition to genetic variation, interest has grown regarding other possible sources of heritable variation between individuals. Of these, methylation of cytosine residues (DNA methylation) act as an epigenomic mark that largely targets transposable elements and other repetitive sequence of the genome to prevent transposition and possibly silence cryptic promoters (Law and Jacobsen 2010; Weigel and Colot 2012; Diez et al. 2014; Kim and Zilberman 2014; Matzke and Mosher 2014). The function of DNA methylation also appears to impact gene expression largely through down-regulation via promoter methylation (Bucher et al. 2012), or possible up-regulation via gene body methylation (Zilberman and Henikoff 2007; Teixeira and Colot 2009; Maunakea et al. 2010). DNA methylation is one of a number of genome modifications that may be able to create an “epiallele” that can be inherited independently of any underlying genetic variation (Eichten et al. 2014). Although a number of genome-scale analyses of DNA methylation and its relationship to genome variation, chromatin modifications, and transcription have been undertaken in plants (Zilberman et al. 2007; Cokus et al. 2008; Lister et al. 2008; Schmitz et al. 2011, 2013; Chodavarapu et al. 2012; Miura et al. 2012; Eichten et al. 2013; Regulski et al. 2013; Stroud et al. 2013; Zhong et al. 2013), the relationship of DNA methylation relative to other classes of genetic variation and patterns of genomic organization, within and among species, is still emerging (Seymour et al. 2014; Dubin et al. 2015; Stuart et al. 2016; Quadrana et al. 2016).

Advances in DNA methylation profiling have allowed a number of plant species to be profiled at the whole-genome level (Zilberman et al. 2007; Cokus et al. 2008; Lister et al. 2008; Schmitz et al. 2011, 2013; Chodavarapu et al. 2012; Miura et al. 2012; Eichten et al. 2013; Regulski et al. 2013; Stroud et al. 2013; Zhong et al. 2013), leading to a basic understanding of the broad patterns of DNA methylation within the genome. The model cereal, *Brachypodium distachyon*, provides a unique plant system to study DNA methylation. With a small diploid genome (∼271 Mb), high genetic diversity (Vogel et al. 2009), global distribution (Garvin et al. 2008), and close relationship to barley and wheat (Draper et al. 2001), it provides a unique and important model system to investigate the function of DNA methylation. Brachypodium is a monocot with a genome size that is highly amenable to sequencing analyses compared to crop systems such as maize (Schnable et al. 2009), barley (International Barley Genome Sequencing Consortium et al. 2012), or wheat (International Wheat Genome Sequencing Consortium 2014). To complement recent genomic sequencing efforts in this model species (International Brachypodium Initiative 2010; Gordon et al. 2014), an understanding of the *Brachypodium* chromatin landscape can provide insights as to the potential effects of transposable element insertions, chromatin accessibility, and functional consequences of differential methylation in this globally diverse plant system.

DNA methylation in plants shows strong regional placement to target transposable element sequences for repression while also targeting other nonrepeat sequences within the genome (Zilberman et al. 2007; Cokus et al. 2008; Lister et al. 2008; Schmitz et al. 2011, 2013; Chodavarapu et al. 2012; Miura et al. 2012; Eichten et al. 2013; Regulski et al. 2013; Stroud et al. 2013; Zhong et al. 2013). Gene body methylation has been compared among orthologous genes in plants indicating a strong conservation of this intragenic methylation (Takuno and Gaut 2013) across species. Recent evidence in Brassicaceae has also shown that DNA methylation variation between species is tied to regions of genomic variability driven largely by transposable elements (Seymour et al. 2014). Because a major function of DNA methylation in plants is as a repressor of transposable element activity (Kim and Zilberman 2014), DNA methylation is highly correlated to the positions of known transposable elements within genomes. Beyond targeting repetitive elements directly, DNA methylation can often “spread” outside of the element boundary and influence nearby sequence (Hollister and Gaut 2009; Hollister et al. 2011; Ahmed et al. 2011; Eichten et al. 2012). From this, it is believed that differentially methylated regions (DMRs) can be driven by transposon insertion polymorphisms in which the presence or absence of an element dictates methylation levels in flanking low-copy sequence. Recent work across more than 200 natural *Arabidopsis thaliana* accessions have indicated that a large number of novel transposable element insertions and deletions are associated with local DMRs (Quadrana et al. 2016; Stuart et al. 2016). From this, transposon polymorphism is possibly a driver of genetically controlled DNA methylation variation, however the ability to identify novel TE insertions, and therefore prospective methylation variants, has been limited to date (van Opijnen and Camilli 2013; Nakagome et al. 2014; Quadrana et al. 2016; Stuart et al. 2016).

To investigate the DNA methylation landscape of *Brachypodium distachyon*, we profiled seven recently resequenced lines (Gordon et al. 2014) by whole-genome bisulfite sequencing ([WGBS] by post bisulfite adapter tagging [PBAT]) to obtain base pair resolution profiles of DNA methylation throughout their genomes. Differentially methylated regions (DMRs) across the different DNA methylation sequence contexts (CG, CHG, and CHH, in which H represents A, C, or T) are prominent across these lines when contrasted to the Bd21 reference genome. Genetic diversity between lines in the form of SNPs, but not newly identified transposable element insertion polymorphisms, aligns with many of the identified DMRs. These results highlight the importance of studying species beyond *Arabidopsis* and provide a glimpse of the interactions between genetic diversity and chromatin states within this important model cereal.

## Results

### DNA methylation patterns of the Bd21 reference genome

**Figure 1.**
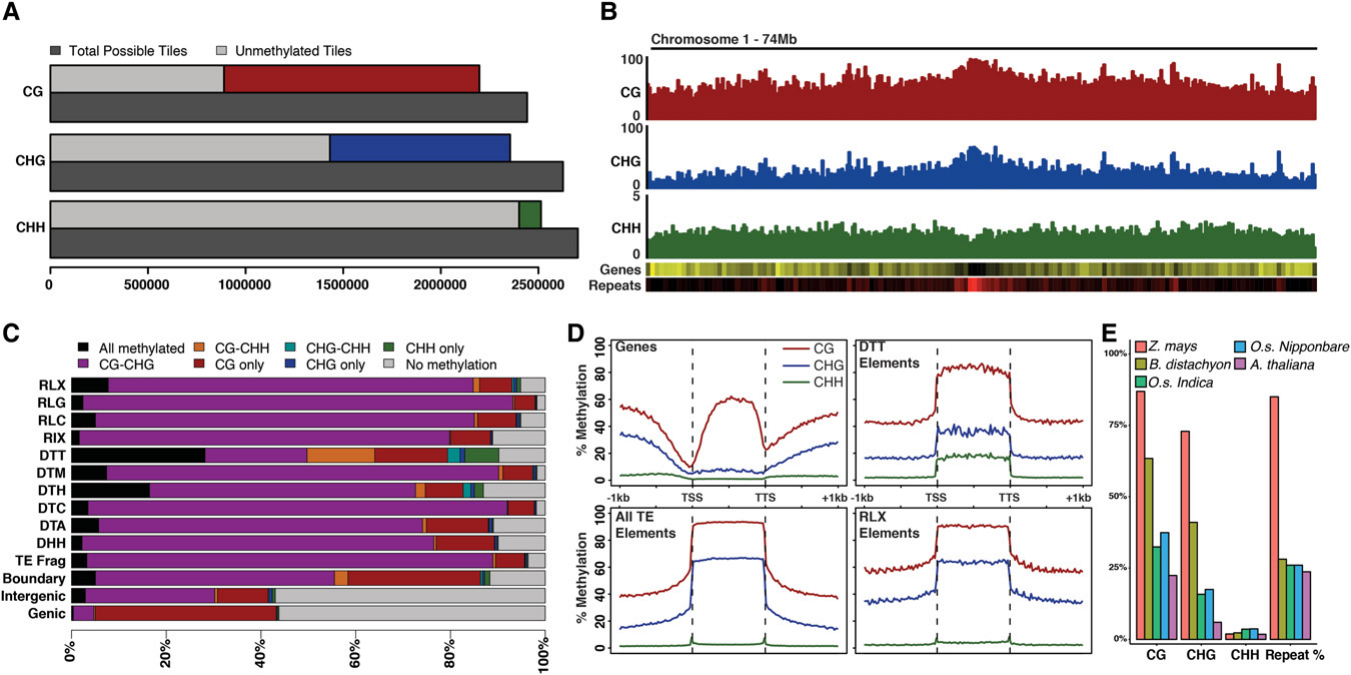
Genomic profiles of DNA methylation of *Brachypodium distachyon*. (*A*) Total number of 100-bp genomic tiles (dark gray) are compared to the number of genomic tiles with sequence coverage for all three methylation sequence contexts. Coverage bar is split between unmethylated tiles (light gray) and methylated tiles: (red) CG; (blue) CHG; (green) CHH. (*B*) Chromosome profile of average methylation in CG, CHG, and CHH contexts. Gene density (yellow high) and repeat density (red high) are also shown. (*C*) Percentage bar plot of genomic tile annotation states within the genome. Bars are divided by eight possible Bd21 methylation states. Retrotransposable superfamilies are indicated by an “R” prefix and DNA elements indicated by a “D” prefix. (*D*) Relative methylation of annotated genes, all transposons, DTT transposons only, and RLX transposons only. Flanking 1 kb of genes and repetitive elements are also shown outside the dashed vertical lines. (*E*) Average level of CG, CHG, CHH, and proportion of genomes consisting of repetitive sequences across the genome of various grass species.

To investigate DNA methylation patterns in the *Brachypodium distachyon* reference genome Bd21, WGBS via PBAT was performed and 100-bp paired-end reads were aligned resulting in approximately 22 million unique alignments with an average coverage of 8.1× (Supplemental Table S1). Although the sequencing data provide base pair resolution of cytosine methylation, broader patterns of DNA methylation encompassing multiple neighboring cytosines can highlight the importance of DNA methylation in maintaining chromatin states throughout the genome. Therefore, average DNA methylation patterns across the chromosomes using nonoverlapping 100-bp tiles were calculated for each of the three sequence contexts. The Bd21 reference genome contains approximately 2.4–2.7 million tiles with at least a single cytosine for each respective methylation context (Fig. 1A). The WGBS performed provides coverage across ∼90% of all possible tiles for each methylation context. DNA methylation levels across genomic tiles display a largely bimodal distribution for CG and CHG methylation levels (Supplemental Fig. S1). From this, tiles were called either “methylated” or “unmethylated” based on these distributions, requiring >50% weighted methylation (Schultz et al. 2012) for CG, 30% for CHG, and 10% for CHH. The proportion of methylated tiles for CHH methylation is much smaller than the CG and CHG contexts (Fig. 1A). When viewing methylation levels across chromosomes, higher levels of CG and CHG methylation are visible in the gene sparse and repeat rich pericentromeric regions (Fig. 1B). This is in contrast to CHH methylation which, although an order of magnitude less abundant than CG or CHG methylation, displays a more uniform distribution across the chromosome (Fig. 1B).

With each genomic tile classified based on its methylation type, a total of eight possible methylation states, ranging from no methylation to fully methylated in all contexts, can be identified for windows containing coverage across all cytosine contexts. The majority of genomic tiles display no methylation, with the remaining tiles largely falling into “CG-only” and “CG-CHG” methylation states (Supplemental Fig. S2). DNA methylation patterns may differ across different genomic features such as genes and transposable elements. Therefore, each genomic tile was grouped based on its nearest intersecting annotated feature (Supplemental Fig. S3). Twenty-eight percent of genomic tiles are found in TEs, 33% in genes, 37% are intergenic, and 2% overlap both genes and TEs. The type of DNA methylation within genes and transposon families provides evidence for different methylation profiles for different features (Fig. 1C). Genic and intergenic genomic tiles are frequently unmethylated compared to transposable element regions. Genic methylation is largely CG-only with intergenic regions displaying CG-only and CG-CHG tiles predominantly. Transposable elements are largely methylated, with CG-CHG-only tiles being most common. These patterns are consistent with other studies in plants; however, the DTT class of Sub1/Mariner elements shows a unique pattern of methylation with an increase in CHH methylation compared to other TEs (Fig. 1C; Supplemental Fig. S4). Genomic tiles that intersect the boundaries of both genes and TEs contain a combination of methylation states found in both genes and TEs. Overall, DNA methylation patterns are substantially different between annotation features, with DTT Mariner elements constituting a proportionally large source of CHH methylation within the genome (∼8% of all CHH-containing tiles in the genome are found in the ∼1% of genomic tiles that are DTT elements). The DTT elements appear to be some of the smaller transposons within the *Brachypodium* annotation and are often found near genes (Supplemental Fig. S5). The DTT elements may be targets of RNA-directed DNA methylation (RdDM) through the activity of 24-nt siRNAs (Matzke and Mosher 2014). An examination of small-RNA data publically available (International Brachypodium Initiative 2010) identified an enrichment of 24-nt siRNAs over DTT elements when normalized for superfamily abundance in the genome (Supplemental Fig. S6).

DNA methylation is known to target genes and transposable elements differently. Previous reports of DNA methylation within *Brachypodium distachyon* have indicated a high proportion of CG methylation within annotated gene bodies (Takuno and Gaut 2013). When comparing all annotated genes in the Bd21 reference genome, a clear pattern of CG gene body methylation was apparent with minimal levels of CHG and CHH methylation (Fig. 1D). This body methylation was maintained with intronic sequence removed, indicating gene body methylation is not a byproduct of intron content (Supplemental Fig. S7). There is a sharp decrease in CG and CHG methylation surrounding the transcription start site. In contrast, methylation patterns are strikingly different when averaged across genomic repeats in the Bd21 reference genome (Fig. 1D). CG methylation is close to a saturating level across repetitive elements, and CHG methylation is also increased compared to genes, although to a lesser extent. For both genes and repeats, DNA methylation appears to return to genomic-average levels once outside the boundaries of the annotated features (Fig. 1D). When genes and transposable elements were divided based on size, CG gene body methylation shows distinct increases positively correlated with gene size leading to minimal methylation for genes smaller than 2 kb (Supplemental Fig. S8). In contrast, repetitive elements of all sizes display high levels of CG methylation. CHG and CHH methylation is highest in smaller transposable elements and shows decreasing levels as repeat size increases (Supplemental Fig. S8). DTT elements, which are some of the smallest annotated transposable elements in the genome (with an average length of 121 bp), display elevated levels of CHH as indicated via tile analysis (Fig. 1D). This is in contrast to other repeat superfamilies such as the larger RLX elements (average length of 842 bp), which do not show elevated levels within the element boundaries.

Plant genomes differ greatly in their repetitive DNA content (i.e., transposable elements). A comparison of *Brachypodium distachyon* global methylation levels to other plant species was performed using published WGBS reads, analyzed with the same alignment parameters (Methods). When comparing average methylation level to the published repeat content of each genome (Goff et al. 2002; Yu et al. 2002; Schnable et al. 2009; International Brachypodium Initiative 2010; Maumas and Quesneville 2014), there is a clear positive association between repeat content, CG, and CHG methylation (Fig. 1E). In contrast, asymmetric CHH methylation does not appear to correlate with genomic repeat proportion. Bd21 displays 2.3% genomic CHH methylation compared to 1.8% in *Arabidopsis thaliana* and 3.7% in *Oryza sativa*. Curiously, Bd21 displays a higher proportion of CG and CHG methylation relative to its genomic repeat content compared to other species. Genic DNA methylation appears similar between species (Supplemental Fig. S9), with similar patterns of CG gene body methylation for all contexts.

Initial genome sequencing of the Bd21 reference line showed that Chromosome 5 displayed the lowest gene density and increased retrotransposon density compared to other chromosomes (International Brachypodium Initiative 2010). Given the positive relationship between repeat content and methylation level, overall DNA methylation levels were compared between chromosomes (Supplemental Fig. S10). As expected, Chromosome 5, with the highest repetitive content, displayed the highest levels of CG and CHG methylation compared to other chromosomes. Overall, DNA methylation in Bd21 follows similar patterns of methylation that have been observed in other plant species (Cokus et al. 2008; Lister et al. 2008; Chodavarapu et al. 2012; Eichten et al. 2013; Mirouze and Vitte 2014), with a slight increase in overall symmetric methylation levels given its genomic repeat content.

### Differential methylation across diverse *B. distachyon* samples

DNA methylation is known to vary among individuals of a species (Vaughn et al. 2007; Zhang et al. 2008; Eichten et al. 2013; Schmitz et al. 2013) and can act as a source of heritable variation impacting gene expression (Stokes et al. 2002; Vaughn et al. 2007; Zilberman et al. 2007; Becker et al. 2011; Schmitz et al. 2011). To investigate DNA methylation variation within *Brachypodium distachyon*, six additional WGBS profiles were created for the inbred lines Bd21-3, Bd3-1, Bd30-1, BdTR12c, Koz-3, and Bd1-1 (Supplemental Table S1). These lines were chosen because they have recently been sequenced as additional reference lines and are commonly used divergent strains within the species (Vogel et al. 2009; Gordon et al. 2014). Reads obtained for each sample were aligned to the SNP-corrected reference genomes corresponding to the selected inbred lines (Gordon et al. 2014). Overall patterns of methylation are highly similar between lines (Supplemental Fig. S11), indicating limited broad-scale variation. The resulting methylation data was used to identify differentially methylated regions (DMRs) between these lines.

**Figure 2.**
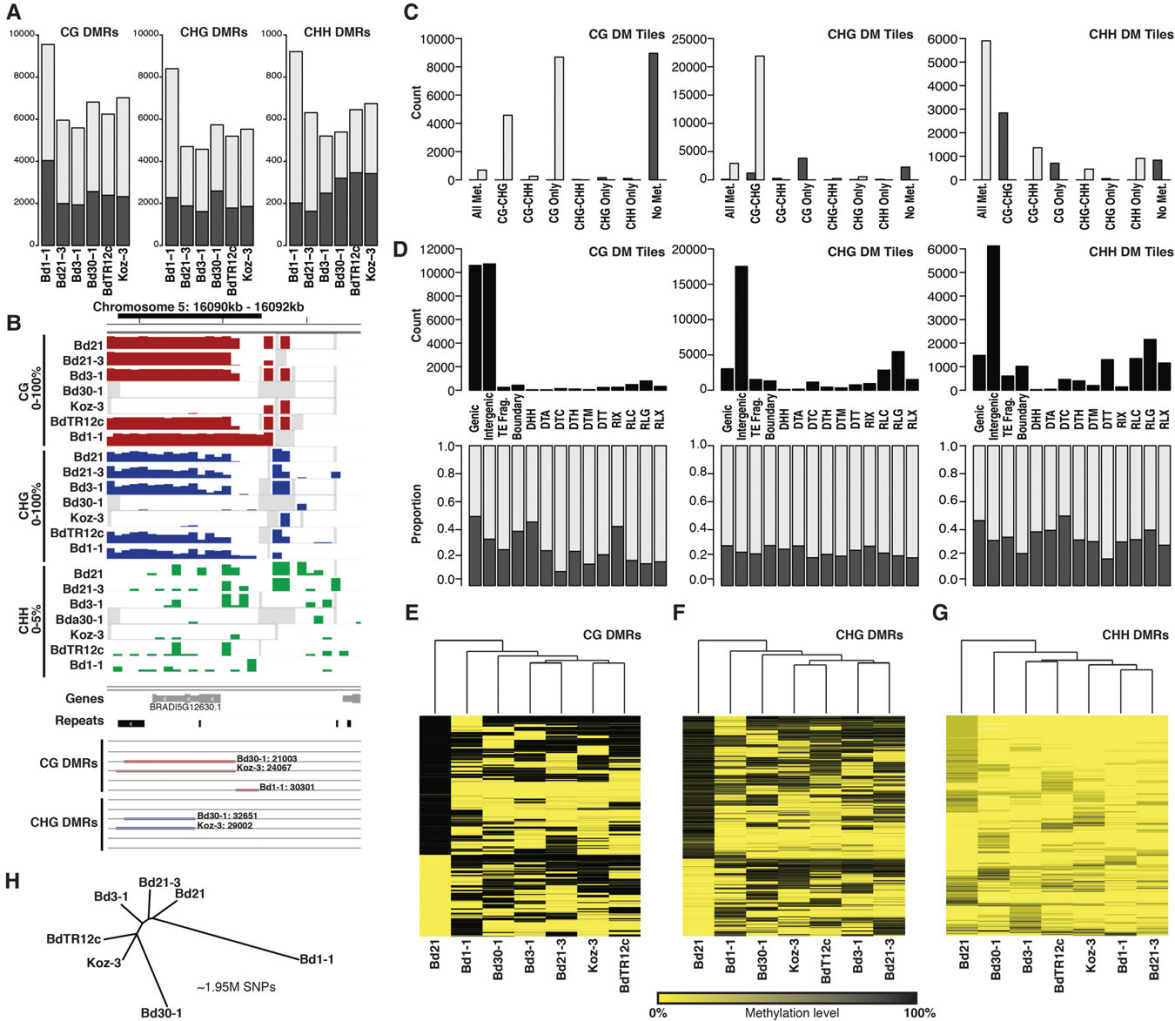
Differential methylation across *Brachypodium* inbred lines. (*A*) Total number of CG, CHG, and CHH DMRs classified for each of the six diverse inbreds. Gray and black indicate low and high methylation compared to Bd21, respectively. (*B*) An example DMR with 100-bp averaged methylation profiles for all seven inbreds. Gene and repeat models are provided *below*. Bd30-1, Koz-3, and Bd1-1 CG/CHG DMRs are highlighted. Gray windows indicate regions of no data (no read coverage). (*C*) Number of differentially methylated tiles across eight Bd21 methylation classes. Gray and black indicate low and high methylation compared to Bd21, respectively. (*D*) The total number of DM tiles (*top*) and proportion of low (gray) and high (black) methylation for DM tiles (*bottom*) across genomic annotation classes. (*E*–*G*) Hierarchical clustering of all identified CG, CHG, and CHH DMRs. Dendrogram indicates overall similarity between samples. Given all DMRs discovered against Bd21, Bd21 acts as an outgroup in this analysis. (*H*) Neighbor-joining tree of the seven reference lines based on 1.95 million SNPs with calls for all samples. SNPs encoded as Bd21 or alternate.

The impact of qualitative differential methylation within the genome appears to have more functional consequences when viewed as regions of multiple cytosines rather than single sites (Becker et al. 2011; Schmitz et al. 2011). Therefore, DNA methylation for all three contexts was averaged independently across nonoverlapping 100-bp windows. Also, because methylation levels between sequence contexts are maintained through different mechanisms (Law and Jacobsen 2010), it is important to classify differential methylation for each context independently. A series of filters was used to classify a DMR window as a CG, CHG, or CHH DMR: For each genotype pairwise comparison, differential methylation was calculated as two or more concurrent windows with at least 3× coverage between both lines and at least two cytosines with coverage that have a difference in methylation of at least 70% (CG) or 50% (CHG; Methods). After collapsing adjacent DMR windows, 5588–9550 CG-DMRs and 4568–8396 CHG-DMRs were identified across the genome for each line when compared to the Bd21 reference (Fig. 2A; Supplemental Table S2). CHH methylation is present at much lower abundances than CG/CHG (Fig. 1A,B) and therefore requires separate criteria for DMR discovery. CHH DMRs were classified as two or more concurrent windows with at least 3× coverage and at least eight cytosines with coverage within the analyzed windows. Differential methylation required one sample to display “low” (≤5%) and the other “high” (≥20%) methylation to be considered a DMR. In total, 520–921 CHH DMRs were identified between the diverse lines and Bd21 (Fig. 2G; Supplemental Table S3).

For all three methylation sequence contexts, the vast majority (∼99%) of genomic tiles do not display differential methylation. However, differential methylation was apparent in certain regions of the genome (Fig. 2B). We observed that differentially methylated (DM) tiles often display changes only in specific DNA methylation state combinations (Fig. 2C, Bd1-1 shown as example). For CG DM tiles, high methylation (when compared to the Bd21 reference) was almost exclusively found in tiles without methylation in any context for Bd21. Low CG methylation compared to Bd21 is most common across Bd21 tiles displaying CG, or both CG and CHG (CG-CHG), methylation. CHG DM tiles appear to come from a largely different set of genomic tiles with high CHG methylation events occurring most often in Bd21 regions displaying CG methylation. Low CHG methylation occurs for tiles where Bd21 displays CG-CHG methylation. High CHH methylation DM tiles also are most common where Bd21 displays CG-CHG methylated tiles. Surprisingly, low CHH methylation is predominantly derived from genomic tiles where Bd21 is methylated in all three sequence contexts (Fig. 2C). Overall differential methylation across the three sequence contexts appears to be derived from different chromatin states when considering their methylation state in Bd21.

Beyond comparisons of methylation state, DM tiles are found in different genomic features depending on sequence context (Fig. 2C, Bd1-1 shown). CG DM tiles are almost all found in genic or intergenic regions. Very few DM tiles are found in any transposable element classes. However, low CG DMR tiles appear enriched for most transposable element classes compared to the overall number of low DM tiles (60%) (Fig. 2D, bottom graph). CHG DM tiles most commonly display a low methylation state compared to the reference (75%). CHG DM tiles are found within intergenic regions most often with no evidence of annotation-specific enrichment of DM direction. CHH DM tiles, although predominantly intergenic, are much more common across transposable elements. The majority of annotated features display a similar proportion of methylation differences for CHH DM tiles. Although the Bd1-1 DM tiles are shown (Fig. 2C,D), these patterns appear largely conserved across the other five lines compared (Supplemental Fig. S12).

Individual 100-bp tiles can further be collapsed into larger regions of differential methylation (Methods). DMRs were found throughout all five chromosomes and were slightly depleted within gene-poor regions of the genome (Supplemental Fig. S13). DMRs ranged in size from 200 bp (minimum size allowed) to as large as 4.7 kb. CHH DMRs tend to be much shorter than CHG or CG DMRs (Supplemental Fig. S14). DMRs between lines displayed considerable overlap, with >30% of CG-DMRs found in one line at least partially overlapping with a DMR from a different comparison. Across the three context-specific types of DMRs identified, patterns highlighting the relationship between sequence contexts were easily identified (Supplemental Fig. S15). The majority of CG DMRs (65%) also display differential methylation in the CHG context (Supplemental Fig. S15). CHG DMRs show a similar pattern in respect to CG methylation; however, only ∼23% of CHG DMRs also display differences in CG methylation. Of the CHG DMRs that do not display differences in CG methylation state, almost all (∼99%) have high levels of CG methylation. CHH DMRs showed a less distinct pattern of increased methylation in other contexts. CG and CHG methylation is largely stable in CHH DMRs (Supplemental Fig. S15).

DNA methylation states for each DMR were calculated for all seven inbreds to identify common states that are shared between lines (Fig. 2B; Supplemental Table S2). To investigate the relationship between samples, hierarchical clustering of DMR states across all samples was performed (Fig. 2E–G). Clustering indicates that many DMRs display differential methylation in only one or two of the seven samples and may be rare variants unique to the individual line. Beyond Bd21, which acts as a pseudo out-group given its relationship to DMR identification, Bd1-1 is the most diverged line compared to the rest for both CG and CHG DMR sets. This, combined with Bd1-1 displaying the highest number of DMRs for all contexts (Fig. 2A), indicates that Bd1-1 contains the most diverged chromatin state compared to the other examined lines. This is consistent with the genetic relationship between lines as seen when constructing a neighbor-joining tree from over 2 million filtered SNPs (Fig. 2H; Gordon et al. 2014).

### Biological replicates quantify heritable methylation variation between genotypes

Many attempts to profile absolute DNA methylation levels have often relied on single replicate data and qualitative analysis, as presented above, because of high experimental cost. However, single-replicate data does not allow for the direct measurement of methylation variability among biological replicates and therefore limits the ability to robustly identify DMRs that consider intra-genotype variation. Recent work investigating Bd21 methylation patterns showed that there is a level of biological variability among biological replicates of the same inbred line (Roessler et al. 2016), highlighting the need to acknowledge intra-genotype variability and stability of DNA methylation.

**Figure 3.**
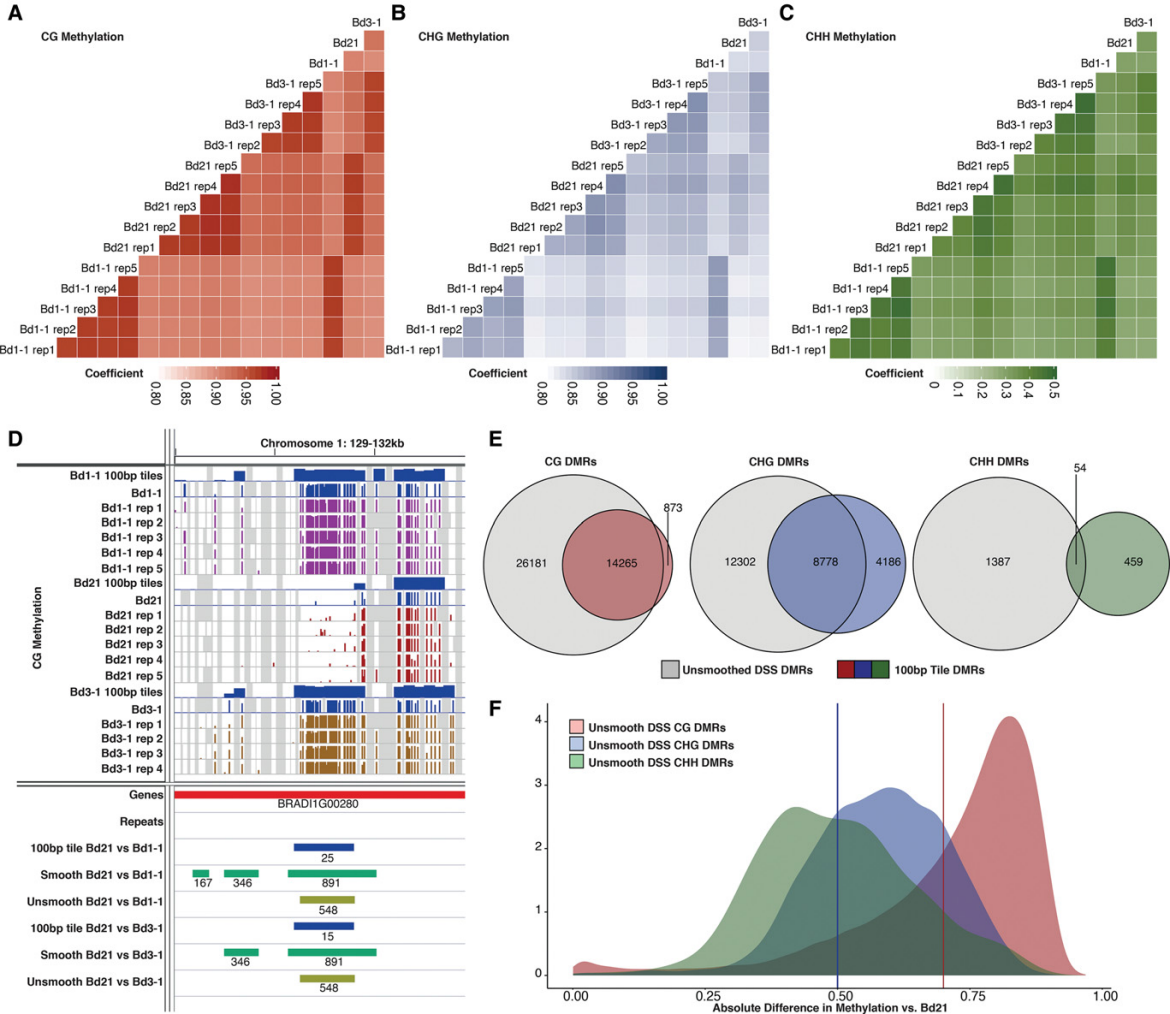
Comparisons of biological replicates of whole-genome bisulfite sequencing. (*A*–*C*) Pearson correlation plots of all genomic cytosines with coverage: (*A*) CG; (*B*) CHG; (*C*) CHH methylation. Note different scale for CHH methylation compared with the other two contexts. (*D*) Genome view of a CG DMR highlighting the 100-bp windowing (blue), smooth DSS (green), and unsmooth DSS (olive) DMRs called. Tracks of cytosine methylation, scaled 0%–100%, are shown along with matching experimental sample and resulting 100-bp window track. (*E*) Venn diagrams of unsmoothed DSS-based DMRs compared to 100-bp tile-base DMRs for CG, CHG, and CHH DMR sets. All DMRs for both Bd1-1 and Bd3-1 comparisons to the reference were included. (*F*) Density distribution of methylation differences between accession groups in unsmoothed DSS DMRs. Red vertical bar indicates fixed cutoff for 100-bp CG DMRs. Blue vertical bar indicates fixed cutoff for 100-bp CHG DMRs.

To investigate the biological variability of DNA methylation within an inbred line, 14 additional samples consisting of five replicates of Bd21, Bd1-1, and four replicates of Bd3-1, were analyzed by WGBS (Supplemental Table S4; Methods). Overall methylation values correlated highly (*r*^2^ ∼ 0.9) with single-replicate data of the previous experiment, and replicate samples showed high conservation for CG and CHG methylation states (Fig. 3A,B). CHH methylation is constantly reestablished de novo with no direct maintenance mechanisms compared to CG and CHG (Law and Jacobsen 2010) and may therefore display greater variability across samples. Indeed, CHH methylation appeared to be more variable with overall lower correlation coefficients (*r*^2^∼0.35) when compared to the other methylation contexts (Fig. 3C). This CHH methylation variability between biological replicates is found regardless of overall CHH methylation level, such as genomic tiles where CHH is elevated well beyond the genomic average (>30%) (Supplemental Fig. S16). Correlations of CHH level are increased by requiring a strict minimum sequencing depth over each site (Supplemental Fig. S17). However, this strict filtering is limiting as it substantially reduces the number of sites that can be profiled within the data set (96% of CHH sites are lost when requiring a minimum of 10 reads/sample). These results indicate that CHH methylation may require a much higher sequencing depth to determine relationships between samples when compared to CG and CHG methylation.

Pairwise differential methylation between the three replicated inbreds was evaluated using the dispersion shrinkage for sequencing data method (DSS) (Feng et al. 2014), which takes into account both the within and between line variation to develop a biologically robust measure of methylation variation at each cytosine position given the sequencing coverage available at each cytosine position. The resulting data was then collapsed into DMRs under the default package parameters to identify regional DNA methylation variation (Fig. 3D). A larger number of DMRs with smaller changes in DNA methylation were identified when the full replicate set was included in the analysis (Supplemental Fig. S18). The DSS method of analysis allows for the imputation of missing data by smoothing based on surrounding cytosine levels. When smoothing is allowed, an order of magnitude increase in the number of DMRs was found (Table 1). DMRs varied in size, with a median size of ∼1500 bp (Supplemental Table S5). Smoothed DSS data produces a large number of DMRs, but many of these appear to be called over regions completely lacking coverage across the replicates of one assayed accession, and these are unlikely to be true DMRs but rather attributable to genetic divergence and difficulties with read mapping (Supplemental Fig. S19). To limit the imputation of inflated DMR numbers, DSS was run without smoothing, and methylation count data for all replicates were required for each inbred line examined. This resulted in roughly half the number of CG and CHG DMRs and a substantial reduction in CHH DMRs being called compared to the smoothed data (Table 1; Supplemental Table S6). These unsmoothed DMRs were used for further investigation.

**Table 1.**
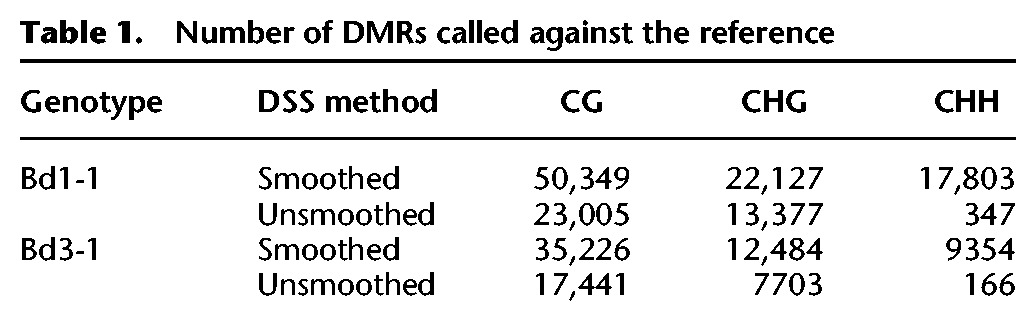
Number of DMRs called against the reference

To investigate the similarities between quantitative DMRs called using DSS and qualitative DMRs from 100-bp tiles, the data sets were intersected (Fig. 3E). For CG DMRs, ∼94% (14,265 of 15,138) of the combined Bd1-1 and Bd3-1 100-bp DMRs intersected with unsmoothed CG DMRs. However, ∼60% of unsmoothed CG DMRs are novel and missed from the qualitative analysis. Overlapping DMRs show a very different pattern for CHG and CHH DMRs with ∼68% and ∼4% of 100-bp DMRs intersecting the unsmooth DSS DMRs, respectively. Overall, there are many DMRs unique to the unsmoothed DSS method and minimal overlap for CHG and CHH DMRs between methods, highlighting the importance of accounting for biological variation in methylation levels when calling DMRs. This is particularly true for the more variable CHG and CHH contexts. However, it should be noted that unsmoothed DSS DMRs and 100-bp DMRs display similar positional relationships to annotated genes and transposable elements within the genome (Supplemental Fig. S20).

Although DNA methylation is a binary state at an individual cytosine, methylation levels are identified from a pool of cell types and lead to proportional differences. The DNA methylation level could be considered a quantitative trait rather than a binary state, as DSS analysis assumes. The unsmoothed DSS DMRs largely highlight differences in methylation of ∼80% for CG methylation, ∼30%–80% for CHG, and ∼20%–90% for CHH (Fig. 3F). The majority of DMRs being called by the unsmoothed DSS method display methylation differences at, or beyond, the filtering requirements of the 100-bp CG or CHG DMRs (Fig. 3F, vertical bars; Supplemental Fig. S21). However, there are 13,412 (∼33%) CG, 5640 (∼27%) CHG, and 116 (∼23%) CHH DMRs from the unsmoothed DSS method that are called as significant differences, which would have been omitted from the tile-based analysis as methylation differences are lower than the required threshold. These additional DMRs highlight the value of replicate data in providing additional power to identify variants with smaller overall changes in methylation.

### DNA methylation variation is correlated with genetic variation between lines

**Figure 4.**
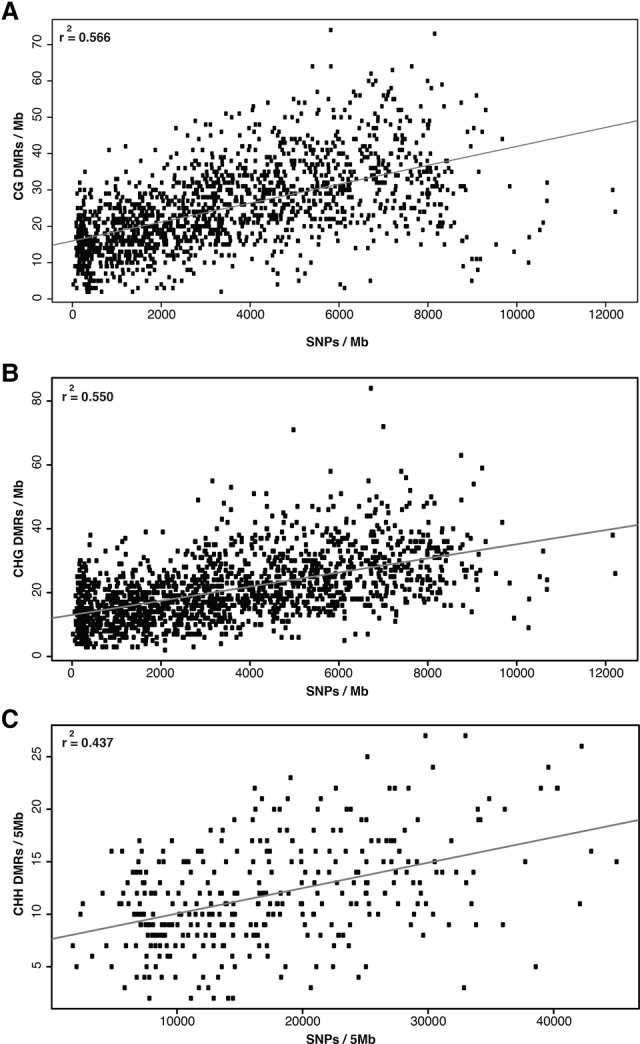
DMR density correlated with SNP density across CG (*A*), CHG (*B*), and CHH (*C*) sequence contexts. CG and CHG density was calculated over nonoverlapping 1-Mb windows. CHH density was calculated across 5-Mb windows given fewer CHH DMRs.

Recent evidence has shown that chromatin marks such as DNA methylation patterns along the genome coincide with patterns of genetic variation within the genome (Lisch and Bennetzen 2011; Fedoroff 2012; Rebollo et al. 2012; Weigel and Colot 2012; Eichten et al. 2013). Single nucleotide polymorphism (SNP) rates (Gordon et al. 2014) were compared with 100-bp tile-based DMR frequency throughout the genome (Fig. 4). DMRs for all three sequence contexts were more prevalent in genomic regions containing higher genetic diversity (Fig. 4A–C; Supplemental Fig. S22). These trends were present for each individual sample with stronger correlation values for CG (*r*^2^:0.314–0.699) and CHG (*r*^2^:0.279–0.541) compared to CHH (*r*^2^:0.063–0.545), which may be attributable to limited CHH DMR number (Supplemental Fig. S22).

Although there is a relationship between the local frequency of DNA methylation variation and genetic variation, this does not appear to fully explain the presence of DMRs within the genome. Across all sample contrasts, 873 DMRs are identified in low-diversity regions (>5000 bp per SNP). The correlations observed indicate a similar relationship to previous studies in maize, for which ∼50% of DMRs appear locally associated with SNP state (Eichten et al. 2013). When using unsmoothed DSS DMRs (Supplemental Table S6), similar patterns are observed with lower correlation values (maximum of 0.39) compared to the 100-bp tile-based DMRs (Supplemental Fig. S23).

### Frequent transposable element polymorphisms create novel genetic diversity within the genome

**Figure 5.**
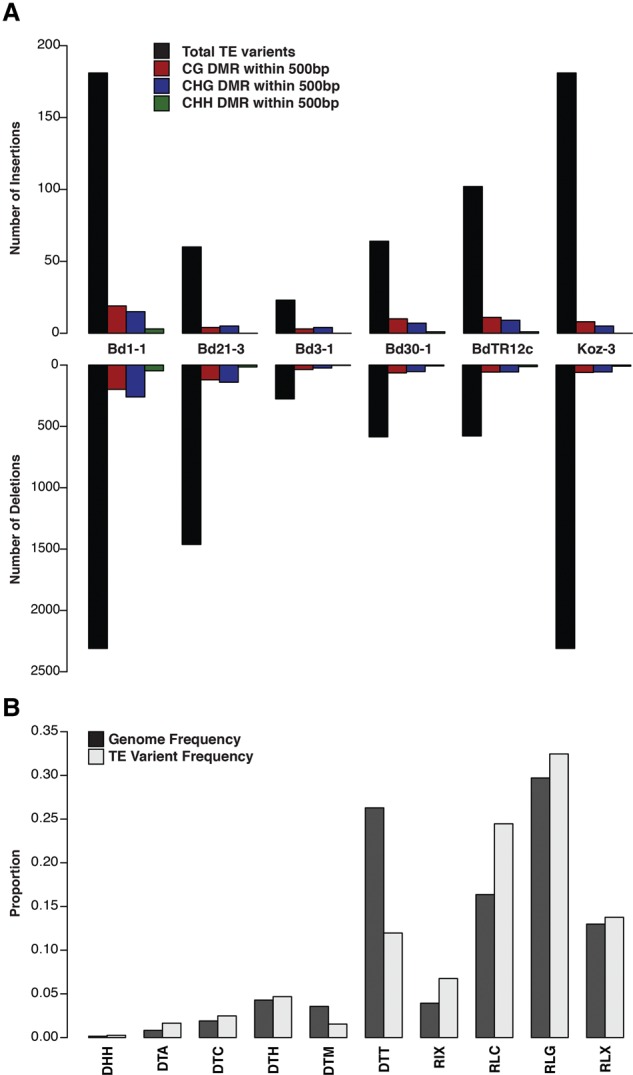
Transposable element polymorphisms across sequenced lines. (*A*) Bar plot indicating total number of transposable element insertions and deletions compared to the Bd21 reference along with the number of DMRs found within 500 bp for each sequence context. (*B*) Bar plot of transposable element families. The genomic frequency of each family (black bars) is compared with the frequency of identified TE insertions and deletion (gray bars).

Transposable elements (TEs) are often targets of DNA methylation (Cokus et al. 2008; Lister et al. 2008; Eichten et al. 2013; Lisch 2013; Vitte et al. 2014). Transposons inserted into new genomic locations may lead to DMRs in the surrounding low-copy sequence though the spreading of DNA methylation from TEs to surrounding sequences (Ahmed et al. 2011; Eichten et al. 2013; Quadrana et al. 2016; Stuart et al. 2016). To investigate the abundance and impact of TE polymorphisms on DNA methylation variation, transposon polymorphisms were identified and compared to the reference Bd21 sequence using the *TEPID* analysis package (https://github.com/ListerLab/TEPID) (Stuart et al. 2016). Paired-end sequencing data was used from the resequencing efforts of the seven reference *Brachypodium* lines (Gordon et al. 2014) to identify polymorphic TE insertion sites within their respective genomes (Methods). In total, 443 novel TE insertions and 3576 deletions were identified across the six non-Bd21 genomes (Fig. 5A; Supplemental Table S7). Similar to SNPs and DMRs, Bd1-1 displayed the most insertions and deletions of all tested genotypes, with Bd21-3 and Bd3-1 displaying the fewest. Bd1-1 is the most genetically distant accession of the set compared to Bd21, with an average of 178 bp between SNPs (Gordon et al. 2014). Bd21-3 and Bd3-1 are the most similar with 537 bp and 488 bp between SNPs, respectively. Surprisingly, Bd21-3 displays a large number of TE deletions given its close relationship with the Bd21 reference (Fig. 5A). The majority (70%) of TE insertions and many (40%) deletions are present just once in one of the genotypes studied (Supplemental Fig. S24). However, there are examples of transposable elements being inserted multiple times within and/or across different genotypes (Supplemental Fig. S25). A nonredundant set of 4019 transposable element polymorphisms is provided in Supplemental Table S7.

The transposable element insertions and deletions were also classified based on the TE family from which they are derived (Fig. 5B). Although the majority of polymorphic TEs are found at a similar distribution to their genomic average, RLC retrotransposons appear to be more polymorphic, and DTT DNA transposons appear less polymorphic. There were no TE families that did not display at least one insertion or deletion event. Overall, hundreds of putative transposon insertions and deletions are identified compared to the reference.

Given the expected relationship between DNA methylation and transposable elements found in other species (Ahmed et al. 2011; Eichten et al. 2013; Quadrana et al. 2016; Stuart et al. 2016), CG, CHG, and CHH DMRs identified between each genotype and Bd21 were compared to the sites of TE insertions or deletions to determine the relationship between nonreference TE sites and methylation variation. Possible associated DMRs were filtered to those within 500 bp of a TE polymorphism as likely local associated features. Surprisingly, there appears to be no clear enrichment for identified DMRs to be near either transposon insertions or deletions (Fig. 5A). This is most prominent in CHH methylation, in which almost no TE polymorphic site has a CHH DMR nearby. It would be expected that new TE insertions and deletions would lead to hypermethylation and hypomethylation, respectively, in the containing genotype. Indeed, a small enrichment for hypermethylation was shown for novel TE insertions as well as hypomethylation for deletions for individual genotypes when compared to the Bd21 reference (Supplemental Fig. S26).

## Discussion

DNA methylation was shown to associate with a wide variety of annotated features, leading to new insights into the mechanisms of genomic repression of transposable elements, as well as gene regulation (Zilberman et al. 2007; Takuno and Gaut 2013; Zemach et al. 2013). The DNA methylation profiles of the Bd21 reference genome, as well as six additional resequenced lines (Gordon et al. 2014) of *Brachypodium distachyon*, provide a useful map of DNA methylation across genetically diverse accessions of a model cereal.

Overall, levels of DNA methylation in *Brachypodium* appear to follow similar patterns to other plant species (Mirouze and Vitte 2014) in which global methylation level is proportional to repeat content of the genome (Fig. 1E). More than half of all CG sites appear methylated across the Bd21 genome (Fig. 1A). This is in contrast with CHG and, especially, CHH methylation that are found at much lower levels. These contexts display a similar enrichment for DNA methylation within pericentromeric regions as previously described in plants (Fig. 1B; Borowska et al. 2011). Patterns of gene-body methylation are similar to those described in the first reported bisulfite sequencing of Bd21 (Fig. 1D; Takuno and Gaut 2013). Curiously, there is limited CHG methylation within gene bodies compared to other monocots such as maize (Supplemental Fig. S9; Eichten et al. 2013). This may be attributable to the higher repeat content of the maize genome (Fig. 1E) and the high proportion of genes containing intronic transposable elements in maize (West et al. 2014).

In comparison, transposable elements of all family types display large amounts of CG and CHG methylation and rarely are found in an unmethylated state. Similar broad patterns were seen in other plant systems (Zhang et al. 2008). The DTT class of DNA transposable elements displays a somewhat unique pattern of methylation with a proportionally large amount of the genome's CHH methylation tiles (Fig. 1C). This class of Sub1/Mariner transposable elements is a common feature of the Bd21 genome with more than 20,000 annotated elements. DTT elements have been noted for high levels of CHH methylation in other species, as a common target of “CHH islands” defining the boundary between active and repressed chromatin (Gent et al. 2013; Li et al. 2015a). As reported in maize, DTT elements are often found near genes as a common target of CHH methylation (Fig. 1D; Supplemental Fig. S5). It is certainly clear that DNA methylation patterns are often unique to specific genomic elements with a wide variety of functions (Diez et al. 2014; Kim and Zilberman 2014).

Differences in DNA methylation between genotypes may provide insight into genomic regulation and chromatin restructuring. With the DNA methylation profiles of six additional accessions, differentially methylated regions (DMRs) were identified compared to the Bd21 reference (Fig. 2A). It should be noted that the vast majority of the genome does not display variable methylation patterns among samples, indicating a largely stable chromatin landscape across the species. Indeed, the majority of methylation patterns appear almost identical across annotated features (Supplemental Fig. S11). Even so, variations in DNA methylation levels were observed across accessions, with thousands of DMRs identified across the three methylation contexts studied (Fig. 2F). The number of DMRs identified in each accession appears to be linked to the underlying genetic distance between the lines as determined by resequencing (Fig. 2D,E; Gordon et al. 2014). DMRs in the CG context display ∼30% overlap between accessions, indicating many conserved methylation variants compared to the reference Bd21, which may be the outlier in certain cases (Fig. 2B,C). Asymmetric CHH methylation variation appears to often arise from transposable element sequences. Because CHH methylation is a de novo modification that requires constant targeting, it is possible that certain transposable elements are being actively silenced by the RNA-directed DNA methylation pathway (RdDM) (Matzke and Mosher 2014). In contrast to CHH methylation, CG DMRs are rarely found in transposable elements (Fig. 2C). This is similar to previous findings in *Arabidopsis thaliana* (Vaughn et al. 2007; Schmitz et al. 2013). As a repressive mark targeting fully silenced transposons (Kim and Zilberman 2014), it is unlikely that CG DMRs would arise from regions of the genome fully marked for heterochromatic silencing. Given the order of magnitude difference in symmetric (CG and CHG) compared to asymmetric (CHH) methylation, it is clear that unique pathways (such as RdDM) (Matzke and Mosher 2014) are major drivers of the methylation state of these DMRs. Therefore, the ability to analyze these DMRs separately is of clear importance.

Biological replication of DNA methylation profiles has largely been lacking to date. By investigating DNA methylation across replicate samples of Bd21, Bd1-1, and Bd3-1, we found high levels of correlation for CG methylation among replicates (Fig. 3A). This correlation was slightly weaker for CHG and substantially less for CHH (Fig. 3B,C). Previous reports in replicated Bd21 bisulfite libraries have indicated a high level of variability among replicates (Roessler et al. 2016). Our data largely supports this for CHH methylation as correlation levels are quite low when compared to other types of genomic assays across replicates (e.g., RNA-seq biological replicates *r*^2^ ∼ 0.99) (Makarevitch et al. 2015). However, the limited CHH correlation between biological replicates may be in part attributable to limited sequencing depth over CHH sites. When sites are filtered to require a minimum of 10–40 reads, correlation levels similar to CG and CHG are observed (Supplemental Fig. S17). Because average CHH methylation is often observed at much lower levels than CG and CHG methylation, it likely requires substantially increased sequencing depth to properly determine its level and relate biological samples to one another.

Symmetric DNA methylation variation is captured often by both single and multiple replicate data (Fig. 3E); however, there are many DSS-based DMRs that appear to be missed by the tile approach (Fig. 3F). Depending on the questions to answer, one may prefer to have a larger number of DMRs as possible candidate variants (combined with a larger chance of false positives). This is in contrast to the conservative approach of multiple filters required for the tile-based DMRs. The two DMR approaches also highlight the variability of CHH methylation across samples and the methods used to identify it. There was almost no overlap among CHH DMRs between methods (Fig. 3E). This lack of conservation between methods may indicate a fundamental limitation in the DMR identification methods at our available sequencing coverage level. However, DSS does include overall depth at cytosine positions as a weighting factor in determining differential methylation. Technically, the limited CHH DMR overlap may be tied to the minimal correlation between biological replicates for this sequence context (*r*^2^ ∼ 0.35) in that there is not likely to be much overlap observable without much deeper coverage. Biologically, as CHH methylation is continually established de novo, it may not show the same level of biological stability when compared to the symmetric methylation contexts that have maintenance methyltransferases to maintain fidelity over DNA replication (Law and Jacobsen 2010).

A major question regarding the study of DNA methylation is the likelihood that observed patterns are either dependent on genetic state or act independently as a separate epigenetic layer of regulatory information (Richards 2006; Eichten et al. 2014). By looking at high-quality SNPs across the reference lines sequenced (Gordon et al. 2014), DNA methylation variation was correlated with increased levels of genetic variation (Fig. 4). For all three contexts, correlation values from 0.42 to 0.56 were observed, indicating that a proportion of all DMRs identified across these diverse lines are likely tied to the genetic variation found nearby. Although this correlation is observed, it does not eliminate the possibility of unlinked DNA methylation variation that acts independent of genetic state. The results show that the relationship between genetics and DNA methylation is clearly complex (Eichten et al. 2014), with some, but not all, DNA methylation variation associated with genetic states.

A possible genetic source of DNA methylation variation may be transposable elements as they are known to be the major target of DNA methylation that acts to suppress their activity (Kim and Zilberman 2014; Mirouze and Vitte 2014). It is possible that variation in transposable element content could create novel targets for DNA methylation and lead to differential methylation between samples (Eichten et al. 2012; Mirouze and Vitte 2014; Quadrana et al. 2016; Stuart et al. 2016). An analysis of paired-end sequencing data of the six resequenced lines identified hundreds of novel transposable element insertions and deletions compared to the Bd21 reference (Fig. 5A). Evidence in maize and *Arabidopsis* has suggested that many DMRs within the genome may be tied to the presence or absence of certain transposable elements (Hollister and Gaut 2009; Ahmed et al. 2011; Hollister et al. 2011; Eichten et al. 2012, 2013; Quadrana et al. 2016; Stuart et al. 2016). Surprisingly, there is no evidence for DNA methylation variation surrounding novel transposable element insertions or deletions (Fig. 5A). It is possible that if some transposon polymorphisms are recent events, they may not be targeted for heterochromatin silencing. It is also possible that these insertions or deletions may be occurring in regions that are already highly heterochromatic, leading to minimal changes in overall DNA methylation patterns in the surrounding regions and limiting sequencing coverage of repetitive regions. Indeed, the overall levels of genome-wide methylation in *Brachypodium distachyon* are higher than those found in *Arabidopsis thaliana*, in which clear associations between transposon variation and DMRs has been seen (Stuart et al. 2016). Additional study as to the differences between plant systems and these genetic polymorphisms will be required to determine the breadth of relationships observed to date across species.

The landscape of DNA methylation and transposable element polymorphisms within diverse accessions of *Brachypodium distachyon* indicate chromatin variation that is often tied to underlying genetic variation. However, there was no clear evidence to tie novel transposable element polymorphisms to nearby DNA methylation variation. Although examples of transposable element presence–absence variation influencing methylation state has been reported (Hollister and Gaut 2009; Ahmed et al. 2011; Hollister et al. 2011; Eichten et al. 2012, 2013; Stuart et al. 2016), other reports indicate minimal association between transposable element variation and methylation (Li et al. 2015b), which may indicate species-specific relationships. Given the relationship between DNA methylation state and genetic background, it would be of interest to investigate natural populations with less genetic variation among them. Preventing large population structure may assist further studies to identify the activity of these methylation variants and novel insertions in relationship to possible functional consequences such as transcriptional regulation.

## Methods

### Tissue collection

Seeds were germinated in moist petri dishes for one week at 10°C and transferred to soil. Plants were grown under 12 h light conditions at 18°C–21°C in controlled growth rooms. Three-wk-old mature leaf tissue was harvested from each of the seven inbred *B. distachyon* lines.

### *Brachypodium* bisulfite sequencing

gDNA was extracted from harvested tissue using Qiagen DNAeasy Plant kit and quantified using the Qubit HsDNA (Life Technologies). Fifty nanograms of purified DNA was bisulfite-converted using the Zymo DNA-Gold bisulfite conversion kit (Zymo Research). Whole-genome bisulfite sequencing (WGBS) libraries were constructed using the EpiGnome Post-Bisulfite Library Kit (Epicentre) according to the manufacturer's instructions (EPILIT405 rev. C). Libraries were quantified on the PerkinElmer GXII and Agilent BioAnalyzer to confirm library quality. Libraries were subsequently pooled and sequenced (Paired-end, 100 bp) across a HiSeq 2000 (Illumina) lane with a 10% PhiX control DNA spiked in for cluster control.

### Read alignment and methylation calling

The resulting reads (Supplemental Table S1) were trimmed to remove adapter contamination and poor quality reads using TrimGalore (http://www.bioinformatics.babraham.ac.uk/projects/trim_galore/). Trimmed reads were then aligned to SNP-corrected versions of the Bd21 reference genome (Gordon et al. 2014) using Bismark (v0.12.5) (Krueger and Andrews 2011). As EpiGnome PBAT libraries appear to create a large number of chimeric reads, alignment had to be performed in three stages to maximize mapping efficiency. First, a traditional directional paired-end alignment through Bismark was performed (flags: -n 2 -l 20 --un). The unmapped “Read 1” reads were then processed through a directional single-end alignment in Bismark (flags: -n 2 -l 20). The unmapped “Read 2” reads were also processed through directional single-end alignment with the additional “--pbat” flag, allowing mapping to the complementary strands. The three resulting alignments were run through the Bismark methylation extractor (flags: --comprehensive --report --buffer_size 8G) in their expected paired-end or single-end modes. Paired-end methylation extraction included the “--no_overlap” flag to prevent counting the same cytosine if covered by both the forward and reverse read. Output was then merged by sequence context (CG, CHG, CHH) and run through bismark2bedGraph (flags: --CX). One hundred–base pair tiled windows providing proportion methylated, number of methylated reads, and number of unmethylated reads across the genome were also created for downstream analysis.

### DMR identification

The number of DMRs identified in this study are similar to other studies in plants. However, the various methods and filtering criteria that are used largely inhibit direct comparisons between lists. The described DMRs in this study are largely filtered to be a conservative estimate of variable methylation sites by requiring strict read count, size, and differential methylation levels and biological reproducibility.

Identification of differentially methylated regions between *Brachypodium* samples was performed using a novel pipeline based on 100-bp tiled windows across the genome. In brief, for each pairwise sample comparison, all windows were called differentially methylated if the absolute difference in proportion methylation met a given threshold (CG 80%; CHG 50%; CHH 20%). Windows were then filtered to require at least 10× coverage across the window to be valid. All adjacent windows were collapsed into a single DMR. All results were compared, and the largest region was kept for any overlapping DMRs between pairwise comparisons. All DMRs used for analysis were mapped to annotated genes (Bdistachyon_192) and genomic repeats (Brachy_TEs_V2.2) (International Brachypodium Initiative 2010) using BEDTools (Quinlan and Hall 2010).

### Replicate methylation data

Third-leaf tissue from five Bd21, five Bd1-1, and four Bd3-1 individuals was harvested, DNA extracted, and libraries prepared as described above. Each plant was isolated separately and used as a biological replicate. One hundred–base pair single-end sequencing on the HiSeq2000 (Illumina) was performed with a 10% PhiX control DNA spiked in for cluster control. DMRs were called using DSS without smoothing and a *Q*-value below 0.01. All other parameters were kept as default. The comparisons were done pairwise with respect to all three lines and sequence context (CG, CHG, CHH).

### Identification of transposon polymorphisms

Transposon polymorphisms were identified using TEPID (https://github.com/ListerLab/TEPID) (Stuart et al. 2016). Briefly, *Brachypodium* paired-end reads sequenced by Gordon et al. (2014) were mapped to the SNP-corrected reference for each accession using Bowtie 2 (Langmead and Salzberg 2012) and YAHA (Faust and Hall 2012), using the mapping script included in the TEPID package with insert sizes estimated from Gordon et al. (2014). TE polymorphisms were then identified by running “tepid-discover” using the *Brachypodium* TE annotation from Gordon et al. (2014), included in the TEPID package, and the “--mask” option set to mask all scaffold chromosomes. Although de novo transposable element annotations are not available for the studied accessions, both the false positive and negative rates of TE discovery are expected to be ∼10% based on prior use cases (Stuart et al. 2016). TE insertion calls were then refined and accessions genotyped for each variant using “tepid-refine.” The resulting variants do not specifically identify the internal sequence of individual TE insertions beyond the expected annotated feature of which it is derived. The final output provides chromosomal positioning of identified TE insertions and deletions within the reference genome.

### SNP density calculation across genome

The overlapping set of SNPs between SOAP and MAQ as identified in Gordon et al. (2014) were used for this analysis. SNPs were subset to those found between Bd21 and each of the six accessions independently and binned by genomic location into 1-Mb (CG, CHG) or 5-Mb (CHH) windows with no overlap. DMRs for each accession and sequence context were binned in a similar fashion using BEDTools (Quinlan and Hall 2010).

### Methylation levels of other plant species

Whole-genome bisulfite sequencing sequence data of other plant species (Supplemental Table S8) were collected from the sequence read archive of associated publications (Lister et al. 2008; Chodavarapu et al. 2012; Eichten et al. 2013). Reads were processed with TrimGalore and Bismark with the same parameters as *Brachypodium* samples. Reads were mapped as either paired-end or single-end, given availability. Supplemental Table S5 lists all samples, alignment metrics, and reference genomes used for non-*Brachypodium* analyses.

## Data access

Whole-genome bisulfite sequencing reads from this study have been submitted to the NCBI Sequence Read Archive (SRA; http://www.ncbi.nlm.nih.gov/sra/) under accession numbers SRX993729 and SRX1270859. Scripts for sequencing alignment and DMR calling are available in Supplemental Data 1.

## Supplementary Material

Supplemental Material
