# Supplementary material for: DNA methylation profiles of diverse Brachypodium distachyon align with underlying genetic diversity
Source: Genome Res. 2016 Nov;26(11):1520–31. doi: 10.1101/gr.205468.116 (PMC5088594; doi:10.1101/gr.205468.116)
Supplement: Supplemental Material [file supp_26_11_1520__index.html]

DNA methylation profiles of diverse Brachypodium distachyon align with underlying genetic diversity — Supplemental Material 

# DNA methylation profiles of diverse *Brachypodium distachyon* align with underlying genetic diversity

## Supplemental Material

- Supplemental\_Data\_1.zip
- Supplemental\_Fig\_S1.pdf
- Supplemental\_Fig\_S2.pdf
- Supplemental\_Fig\_S3.pdf
- Supplemental\_Fig\_S4.pdf
- Supplemental\_Fig\_S5.pdf
- Supplemental\_Fig\_S6.pdf
- Supplemental\_Fig\_S7.pdf
- Supplemental\_Fig\_S8.pdf
- Supplemental\_Fig\_S9.pdf
- Supplemental\_Fig\_S10.pdf
- Supplemental\_Fig\_S11.pdf
- Supplemental\_Fig\_S12.pdf
- Supplemental\_Fig\_S13.pdf
- Supplemental\_Fig\_S14.pdf
- Supplemental\_Fig\_S15.pdf
- Supplemental\_Fig\_S16.pdf
- Supplemental\_Fig\_S17.pdf
- Supplemental\_Fig\_S18.pdf
- Supplemental\_Fig\_S19.pdf
- Supplemental\_Fig\_S20.pdf
- Supplemental\_Fig\_S21.pdf
- Supplemental\_Fig\_S22.pdf
- Supplemental\_Table\_1.xlsx
- Supplemental\_Table\_2.xlsx
- Supplemental\_Table\_3.xlsx
- Supplemental\_Table\_4.xlsx
- Supplemental\_Table\_5.xlsx
- Supplemental\_Table\_6.xlsx
- Supplemental\_Table\_7.xlsx
- Supplemental\_Table\_8.xlsx
- Supplemental\_Fig\_S23.pdf
- Supplemental\_Fig\_S24.pdf
- Supplemental\_Fig\_S25.pdf
- Supplemental\_Fig\_S26.pdf
- Supplemental\_Legends.docx
