# Supplementary material for: DNA methylation profiles of diverse Brachypodium distachyon align with underlying genetic diversity
Source: Genome Res. 2016 Nov;26(11):1520–31. doi: 10.1101/gr.205468.116 (PMC5088594; doi:10.1101/gr.205468.116)
Supplement: Supplemental Material [file supp_gr.205468.116_Supplemental_Data_1.zip › FIGURE1_reanalysis_of_tiles.html]

Reanalysis of Bd21 tiles


# Reanalysis of Bd21 tiles

#### *SRE*

#### *28 July 2015*

The Bd21Control genome was divided into 100bp windows and the number of possible CG, CHG, and CHH sites was calculated. These describe the total number of tiles in the genome that could contain information:

```
all.cg=read.delim('CGsites_100bp.bed',head=F)
all.chg=read.delim('CHGsites_100bp.bed',head=F)
all.chh=read.delim('CHHsites_100bp.bed',head=F)
```

We can then merge these sites together to create a final `all.windows` dataframe:

```
t1=merge(all.cg,all.chg,by=c('V1','V2','V3'),all=T)
all.windows=merge(t1,all.chh,by=c('V1','V2','V3'),all=T)
colnames(all.windows)=c('V1','V2','V3','CGsites','CHGsites','CHHsites')

cg.possible=table(is.na(all.windows$CGsites))[1]
chg.possible=table(is.na(all.windows$CHGsites))[1]
chh.possible=table(is.na(all.windows$CHHsites))[1]
```

For each sequence context, there is a number of possible windows with at least one site. We can now determine how many windows we actually have data for:

```
#read in Bd21 mapped tile data
cg=read.delim('Bd21_CpG_100bp.wig',head=F,skip=1)
chg=read.delim('Bd21_CHG_100bp.wig',head=F,skip=1)
chh=read.delim('Bd21_CHH_100bp.wig',head=F,skip=1)

#get rid of scaffolds
cg=cg[grep('^Bd',cg$V1),]
chg=chg[grep('^Bd',chg$V1),]
chh=chh[grep('^Bd',chh$V1),]

cg=cg[with(cg,order(cg[,1],cg[,2])),]
chg=chg[with(chg,order(chg[,1],chg[,2])),]
chh=chh[with(chh,order(chh[,1],chh[,2])),]

all=merge(all.windows,cg,by=c('V1','V2','V3'),all=T)
all=merge(all,chg,by=c('V1','V2','V3'),all=T)
colnames(all)=c('V1','V2','V3','CGsites','CHGsites','CHHsites','cg_prop','cg_met','_cg_unmet','cg_total','cg_site','chg_prop','chg_met','chg_unmet','chg_total','chg_site')
all=merge(all,chh,by=c('V1','V2','V3'),all=T)
colnames(all)=c('V1','V2','V3','CGsites','CHGsites','CHHsites','cg_prop','cg_met','_cg_unmet','cg_total','cg_site','chg_prop','chg_met','chg_unmet','chg_total','chg_site','chh_prop','chh_met','chh_unmet','chh_total','chh_site')

cg.coverage=table(is.na(all$cg_total)==T)[1]
chg.coverage=table(is.na(all$chg_total)==T)[1]
chh.coverage=table(is.na(all$chh_total)==T)[1]
```

Lets look at the overall distribution of DNA methylation for each class:

```
par(mfrow=c(1,3))
plot(density(all$cg_prop,na.rm=T),main='CG methylation',xlab='Methylation %',col='#990000',lwd=3)
plot(density(all$chg_prop,na.rm=T),main='CHG methylation',xlab='Methylation %',col='#003399',lwd=3)
plot(density(all$chh_prop,na.rm=T),main='CHH methylation',xlab='Methylation %',col='#336600',lwd=3,xlim=c(0,20))
```

From this we can see that methylation is largely bimodal with windows being highly methylated, or showing close to zero methylation.

Of the windows that have coverage, we can make a binary call if a window is deemed methylated or not. For CG this can be 50%, for CHG, this can be 30%. CHH is not bimodal, so we will just say things over 10% are methylated:

```
cg.class=ifelse(all$cg_prop>50,1,0)
chg.class=ifelse(all$chg_prop>30,1,0)
chh.class=ifelse(all$chh_prop>10,1,0)

all=cbind(all,cg.class,chg.class,chh.class)
```

From this binary classification, we can go through and place each window (with coverage across all context) within one of eight catagories:

```
window_class=
ifelse(all$cg.class==1 & all$chg.class==1 & all$chh.class==1,'all_met',
ifelse(all$cg.class==1 & all$chg.class==0 & all$chh.class==0,'cg_only',
ifelse(all$cg.class==0 & all$chg.class==1 & all$chh.class==0,'chg_only',
ifelse(all$cg.class==0 & all$chg.class==0 & all$chh.class==1,'chh_only',
ifelse(all$cg.class==1 & all$chg.class==1 & all$chh.class==0,'cg_chg',
ifelse(all$cg.class==1 & all$chg.class==0 & all$chh.class==1,'cg_chh',
ifelse(all$cg.class==0 & all$chg.class==1 & all$chh.class==1,'chg_chh',
ifelse(all$cg.class==0 & all$chg.class==0 & all$chh.class==0,'no_met',
'NA'))))))))

all=cbind(all,window_class)

barplot(table(all$window_class,exclude=NULL),las=2)
```

From these classes, we can see that the majority of tiles display either CG and CHG methylation, CG only, or no methylation at all. Note that there are also many windows that do not have coverage information across all three sequence context, and therefore cannot be placed in these catagories without ambiguity.

From here, we can bring in annotation information to see if there are specific transposable element classes, or genes, that are enriched for these classes. A bed file was created by taking all Bd21Control windows and identifying the closest gene or transposable element from the published annotations (v2.2):

```
anno=read.delim('all.windows.to.genes.to.tes.bed',head=F)
```

From this file, we can classify each genomic tile as either intersecting a gene, a transposon, or falling into an intergenic region:

```
anno.class=
  ifelse(anno$V11==0 & anno$V19==0, 'TE-gene boundary',
  ifelse(anno$V11==0,'gene',
  ifelse(anno$V19==0,as.character(anno$V16),
  'intergenic')))

anno2=cbind(anno[,1:3],anno.class)
#fix the NA name for transposable element fragments
anno2$anno.class=ifelse(is.na(anno2$anno.class)==T,'TE_frag',as.character(anno2$anno.class))
all=merge(all,anno2,by=c('V1','V2','V3'))
write.table(all,'Bd21_total_tile_annotation.txt',sep='\t',row.names=F,quote=F)
```

With all of this information gathered, we can begin to look at class enrichments across the genome:

```
barplot(table(all$anno.class),las=2,main='Number of tiles across annotation classes')
```

```
barplot(table(all$window_class),las=2,main='Number of tiles across methylation classes')
```

```
prop.met.class=prop.table(table(all$anno.class,all$window_class),margin=2)

par(mar=c(5,4,4,7),xpd=T)
barplot(prop.met.class,col=rainbow(length(rownames(prop.met.class))),las=2)
legend("topright",inset=c(-.15,0),fill=rainbow(length(rownames(prop.met.class))),legend=rownames(prop.met.class))
```

```
prop.anno.class=prop.table(table(all$window_class,all$anno.class),margin=2)
prop.anno.class=prop.anno.class[,c(7,8,14,13,1,2,3,4,5,6,9,10,11,12)]
pdf('fig1c.pdf',width=10,height=5)
par(mar=c(5,4,4,7),xpd=T)
barplot(prop.anno.class,col=c('black','#8B008B','#CD6600','#990000','#008B8B','#003399','#336600','grey'),las=2,horiz=T)
legend("topright",inset=c(-.16,0),fill=c('black','#8B008B','#CD6600','#990000','#008B8B','#003399','#336600','grey'),legend=rownames(prop.anno.class))
dev.off()
```

```
## quartz_off_screen 
##                 2
```

```
barplot(prop.anno.class,col=c('black','#8B008B','#CD6600','#990000','#008B8B','#003399','#336600','grey'),las=2)
legend("topright",inset=c(-.16,0),fill=c('black','#8B008B','#CD6600','#990000','#008B8B','#003399','#336600','grey'),legend=rownames(prop.anno.class))
```

The plots shown above highlight that genes and intergenic regions show a very different proportion of each of the eight methylation classes compared to the transposable element classes. One TE class with a slightly different methylation profile is the DTT or Mariner elements that show a much higher proportion of classes containing CHH methylation compared to the other TE classes.

```
table(all$chh.class)
```

```
## 
##       0       1 
## 2700685  126255
```

```
#126255 total tiles contain CHH)
table(all$window_class,all$anno.class)
```

```
##           
##               DHH    DTA    DTC    DTH    DTM    DTT   gene intergenic
##   all_met      75    227   1814   2034   1118   5599   2769      24573
##   cg_chg     2500   2706  45931   6934  12461   4276  30873     228882
##   cg_chh       17     33     81    255    136   2837   2172       4866
##   cg_only     413    517   2866    988    942   3052 272218      89536
##   chg_chh       3      6     38    197     33    529    278       1788
##   chg_only     17     26    158     93     68    195   1534       5470
##   chh_only      7     10     36    235     38   1427   2196       5459
##   no_met      334    433    967   1612    261   1945 400555     477616
##           
##               RIX    RLC    RLG    RLX TE_frag TE-gene boundary
##   all_met     652   5641   8415   5529    2433             2863
##   cg_chg    30899  89329 313623  54856   64254            28624
##   cg_chh       83    748   1105    954     436             1630
##   cg_only    3321   9083  15026   4870    4587            15870
##   chg_chh      11    189    206    291      98              256
##   chg_only    127    600    724    437     238              221
##   chh_only     42    311    388    580     219              631
##   no_met     4385   5760   6045   3692    2705             6633
```

```
#DTT contains 10392 total tiles contain CHH
prop.table(table(all$anno.class))
```

```
## 
##              DHH              DTA              DTC              DTH 
##      0.001446396      0.001778783      0.020654549      0.005535056 
##              DTM              DTT             gene       intergenic 
##      0.005891869      0.010520204      0.317583614      0.355064812 
##              RIX              RLC              RLG              RLX 
##      0.015968120      0.049858744      0.130442016      0.029561008 
##          TE_frag TE-gene boundary 
##      0.029939606      0.025755224
```

```
#DTT makes up ~1% of all genomic tiles
```

Lets make a summary plot for a figure:

```
plot.table=matrix(NA,nrow=4,ncol=3)
colnames(plot.table)=c('CG','CHG','CHH')
rownames(plot.table)=c('Total tiles with sites','Total tiles with coverage >0','Tiles unmethylated','Tiles methylated')
plot.table[1,]=c(cg.possible,chg.possible,chh.possible)
plot.table[2,]=c(cg.coverage,chg.coverage,chh.coverage)
plot.table[3:4,1]=table(cg.class)
plot.table[3:4,2]=table(chg.class)
plot.table[3:4,3]=table(chh.class)
barplot(plot.table[1:2,],beside=T,horiz=T,las=1)
```

```
barplot(plot.table[3:4,],beside=F,horiz=T,las=1,col=c('#336600','grey','#003399','grey','#990000','grey'))
```

```
pdf('figure_tile_sumary.pdf',width=10,height=6)
barplot(plot.table[1:2,],beside=T,horiz=T,las=1)
barplot(plot.table[3:4,],beside=F,horiz=T,las=1,col=c('#336600','grey','#003399','grey','#990000','grey'))
dev.off()
```

```
## quartz_off_screen 
##                 2
```

We can also look at the overall distribution of the methylation tile classes across chromosome one (unfinished)

```
asub=subset(all,all$window_class=='all_met' & all$V1=='Bd1')
library(fields)
```

```
## Warning: package 'fields' was built under R version 3.1.3
```

```
## Loading required package: spam
```

```
## Warning: package 'spam' was built under R version 3.1.3
```

```
## Loading required package: grid
## Spam version 1.3-0 (2015-10-24) is loaded.
## Type 'help( Spam)' or 'demo( spam)' for a short introduction 
## and overview of this package.
## Help for individual functions is also obtained by adding the
## suffix '.spam' to the function name, e.g. 'help( chol.spam)'.
## 
## Attaching package: 'spam'
## 
## The following objects are masked from 'package:base':
## 
##     backsolve, forwardsolve
## 
## Loading required package: maps
```

```
## Warning: package 'maps' was built under R version 3.1.3
```

```
## 
##  # ATTENTION: maps v3.0 has an updated 'world' map.        #
##  # Many country borders and names have changed since 1990. #
##  # Type '?world' or 'news(package="maps")'. See README_v3. #
```

```
abin=stats.bin(asub$V2,asub$window_class,N=1000)
data=cbind(matrix(abin$centers,ncol=1),abin$stats["N",])
plot(data)
```
