# Supplementary material for: DNA methylation profiles of diverse Brachypodium distachyon align with underlying genetic diversity
Source: Genome Res. 2016 Nov;26(11):1520–31. doi: 10.1101/gr.205468.116 (PMC5088594; doi:10.1101/gr.205468.116)
Supplement: Supplemental Material [file supp_gr.205468.116_Supplemental_Data_1.zip › FIGURE2_DMR_filtering_walkthrough.html]

Working through filter methods for DMR calling of Bd\_7meth samples


# Working through filter methods for DMR calling of Bd\_7meth samples

#### *SRE*

#### *14 January 2015*

First, we have R code run on edmund to take all 100bp wig files and merge them together into a union file of all windows with at least one sample showing data:

```
list=dir(pattern="*CpG_100bp.wig")
list2=matrix(unlist(strsplit(list,"_")),ncol=3,byrow=T)[,1]

out=read.delim(list[1],head=F,skip=1)
colnames(out)[(ncol(out)-4):ncol(out)]=c(paste(list2[1],"percentage",sep='_'),paste(list2[1],"met",sep='_'),paste(list2[1],"unmet",sep='_'),paste(list2[1],"total",sep='_'),paste(list2[1],"sitecount",sep='_'))

for(i in 2:length(list)){
temp=read.delim(list[i],head=F,skip=1)
temp.merge=merge(out,temp,by=c('V1','V2','V3'),all=T)
out=temp.merge

colnames(out)[(ncol(out)-4):ncol(out)]=c(paste(list2[i],"percentage",sep='_'),paste(list2[i],"met",sep='_'),paste(list2[i],"unmet",sep='_'),paste(list2[i],"total",sep='_'),paste(list2[i],"sitecount",sep='_'))

}

write.table(out,'7_geno_CG_100bp_windows_all.txt',sep='\t',row.names=F,quote=F)

#
```

This was done for CG, CHG, and CHH windows leading to three ‘…*windows*all.txt’ files.

Read in the data for CG methylation and pull the columns of interest, although there is a methylation percentage column included, to get around any issues with rounding error, we will recalculate these and reorganize the data:

```
all=read.delim('7_geno_CG_100bp_windows_all.txt',head=T)

names(all)
```

```
##  [1] "V1"                               "V2"                              
##  [3] "V3"                               "Bd1.1_CpG_100bp.wig_percentage"  
##  [5] "Bd1.1_CpG_100bp.wig_met"          "Bd1.1_CpG_100bp.wig_unmet"       
##  [7] "Bd1.1_CpG_100bp.wig_total"        "Bd1.1_CpG_100bp.wig_sitecount"   
##  [9] "Bd21.3_CpG_100bp.wig_percentage"  "Bd21.3_CpG_100bp.wig_met"        
## [11] "Bd21.3_CpG_100bp.wig_unmet"       "Bd21.3_CpG_100bp.wig_total"      
## [13] "Bd21.3_CpG_100bp.wig_sitecount"   "Bd21_CpG_100bp.wig_percentage"   
## [15] "Bd21_CpG_100bp.wig_met"           "Bd21_CpG_100bp.wig_unmet"        
## [17] "Bd21_CpG_100bp.wig_total"         "Bd21_CpG_100bp.wig_sitecount"    
## [19] "Bd30.1_CpG_100bp.wig_percentage"  "Bd30.1_CpG_100bp.wig_met"        
## [21] "Bd30.1_CpG_100bp.wig_unmet"       "Bd30.1_CpG_100bp.wig_total"      
## [23] "Bd30.1_CpG_100bp.wig_sitecount"   "Bd3.1_CpG_100bp.wig_percentage"  
## [25] "Bd3.1_CpG_100bp.wig_met"          "Bd3.1_CpG_100bp.wig_unmet"       
## [27] "Bd3.1_CpG_100bp.wig_total"        "Bd3.1_CpG_100bp.wig_sitecount"   
## [29] "BdTR12c_CpG_100bp.wig_percentage" "BdTR12c_CpG_100bp.wig_met"       
## [31] "BdTR12c_CpG_100bp.wig_unmet"      "BdTR12c_CpG_100bp.wig_total"     
## [33] "BdTR12c_CpG_100bp.wig_sitecount"  "Koz.3_CpG_100bp.wig_percentage"  
## [35] "Koz.3_CpG_100bp.wig_met"          "Koz.3_CpG_100bp.wig_unmet"       
## [37] "Koz.3_CpG_100bp.wig_total"        "Koz.3_CpG_100bp.wig_sitecount"
```

```
f1=all[,5]/all[,7]
f2=all[,10]/all[,12]
f3=all[,15]/all[,17]
f4=all[,20]/all[,22]
f5=all[,25]/all[,27]
f6=all[,30]/all[,32]
f7=all[,35]/all[,37]

data=cbind(all[,1:3],f1,f2,f3,f4,f5,f6,f7,all[,c(7,8,12,13,17,18,22,23,27,28,32,33,37,38)])
colnames(data)[1:10]=c('chrom','start','stop','Bd1-1','Bd21-3','Bd21','Bd30-1','Bd3-1','BdTR12c','Koz-3')
```

We will also need to have the ‘contrast’ values. These are just the diverse lines methylation percentage minus the Bd21 (control) methylation percentage:

```
f1f3=f1-f3
f2f3=f2-f3
f4f3=f4-f3
f5f3=f5-f3
f6f3=f6-f3
f7f3=f7-f3

data=cbind(data,f1f3,f2f3,f4f3,f5f3,f6f3,f7f3)
colnames(data)[25:30]=c('Bd1-1vsBd21','Bd21-3vsBd21','Bd30-1vsBd21','Bd3-1vsBd21','BdTR12cvsBd21','Koz-3vsBd21')
```

To start, we will better understand what thresholds are important by working with Bd1-1 vs Bd21 for now. Therefore we can subset all windows to those that have *any* data in Bd1-1 and Bd21:

```
test=subset(data,data[,11]>=1 & data[,15]>=1)
dim(test)
```

```
## [1] 2094577      30
```

A total of 2.09Million CG sites with data

Some windows have MAJOR coverage (ex over 5000 reads). Looking in IGV, and they look alright, nothing too clonal so perhaps just a super enriched site.

## Looking at the number of windows based on read count filter (for both samples)

```
coverage.prop=NULL
for(i in 1:15){
coverage.prop=c(coverage.prop,nrow(subset(test,test[,11]>=i & test[,15]>=i))/nrow(test))
}
```

```
prop=cbind(matrix(1:15,ncol=1),coverage.prop)
plot(prop, main="Proportion of windows in which\n both samples have met coverage threshold",xlab='total read coverage')
```

At this stage, we want to maximize the number of windows to look at, but still have some coverage threshold (as a window with one read, indicating 0 or 100% methylation does not have power and can largely skew results to all or nothing states). Going with at least three reads maintains ~88% of the windows for further analysis and provides confidence that the results are robust.

## Looking at number of cytosines with coverage in both samples

```
#note, this is dividing by two given CG sites are counted on both + and - strand
hist(test[,12]/2, main='Histogram of cytosines in windows for Bd1-1',xlab='cytosine site count')
```

Doing a similar filter test as above but specfically for the number of cytosine sites (CG,CHG, or CHH) with coverage in the window:

```
site.prop=NULL
for(i in 1:15){
site.prop=c(site.prop,nrow(subset(test,test[,12]>=i & test[,16]>=i))/nrow(test))
}
```

```
site=cbind(matrix(1:15,ncol=1),site.prop)
plot(site, main="Proportion of windows in which\n both samples have met site count threshold",xlab='total cytosine count')
```

So site count starts to decay very quickly compared to read count (as expected really). I think we want to limit this to having at least 2 cytosines, as even that is just ~84% of windows kept. More than that may be just lose too many windows.

## Now for methylation difference between samples (weighted methylation count)

```
hist(test[,25],breaks=200,main='methylation difference between Bd1-1 and Bd21',xlab='methylation difference (Bd1-1 - Bd21)')
```

Vast majority of windows have no difference at all. Lets remove those just to get a better look at the outliers:

```
temp=subset(test,test[,25]!=0)
hist(temp[,25],breaks=200,main='methylation difference between Bd1-1 and Bd21\n (0 difference removed)',xlab='methylation difference (Bd1-1 - Bd21)')
```

Differential methylation is viewed as a binary (on/off) state as far as I am currently concerned. From that, a minimal change should be 60% or so as that would require one sample to be ‘low’ and one ‘high’ in state. At anything less, you could call a DMR for a sample with 100% vs 50% (which is arguably also methylated, or in no man’s land at best).

```
met.prop=NULL
for(i in c(.60,.70,.80,.90,.95)){
met.prop=c(met.prop,nrow(subset(test,test[,25]>=i | test[,25]<=-i))/nrow(test))
}
```

```
met=cbind(matrix(c(60,70,80,90,95),ncol=1),met.prop)
plot(met, main="Proportion of windows in which\n methylation difference is greater than",xlab='Difference in methylation (%)')
```

The higher the difference in methylation, the fewer windows that meet the criteria. Even at a ‘loose’ 60% difference, we are only seeing less than 1% of windows meeting this criteria. A difference of 70% should work while still being fairly accepting.

# So, throw them all together, and how many windows do we see…

```
dmr.windows=subset(data,data[,11]>=3 #Bd1-1 read filter
                   & data[,15]>=3 #Bd21 read filter
                   & data[,12]>=2 #Bd1-1 site filter
                   & data[,16]>=2 #Bd21 site filter
                   & (data[,25] >=0.7 | data[,25]<= -0.7) #methylation difference filter
                   )

nrow(dmr.windows)
```

```
## [1] 56780
```

```
hist(dmr.windows[,25],breaks=200)
```

```
summary(dmr.windows[,25])
```

```
##    Min. 1st Qu.  Median    Mean 3rd Qu.    Max. 
## -1.0000 -0.9630 -0.7778 -0.1555  0.8889  1.0000
```

So we get ~57k windows meeting these criteria. More are negative values, indicating Bd21 has higher methylation than Bd1-1 (in the Bd1-1 - Bd21 difference calculation). If we group nextdoor windows, we can get a distribution of DMR sizes…

```
group.id=c(1,rep(NA,nrow(dmr.windows)-1))
for(q in 2:nrow(dmr.windows)){
  group.id[q]=ifelse(dmr.windows[q,2] - dmr.windows[q-1,2]<=100 & dmr.windows[q,1]==dmr.windows[q-1,1] & dmr.windows[q,25] * dmr.windows[q-1,25] > 0,group.id[q-1],group.id[q-1]+1)
    }

dmr.window.size=table(table(group.id))
barplot(dmr.window.size,main='Number of sequential windows for each DMR',xlab='Number of windows (size = number * 100bp)',ylab='Frequency')
```

```
dmr.windows=cbind(dmr.windows,group.id)

write.table(dmr.windows,'Bd1-1.CG.dmr.windows.txt',sep='\t',row.names=F,quote=F)
sum(dmr.window.size[2:length(dmr.window.size)])
```

```
## [1] 9550
```

So there are mostly DMRs of a single 100bp window in size. If we require a DMR to be at least two windows in sizes (>= 200bp), we would end up with 9620 DMRs rather than 57k. I would certainly put more faith in two concurent windows rather than a single window in the genome.

if we do these filteres for all lines vs Bd21 (control), we get:

```
#bd1-1
results=matrix(NA,ncol=2,nrow=6)
results[1,1]=nrow(dmr.windows)
results[1,2]=sum(dmr.window.size[2:length(dmr.window.size)])

#the rest
for(i in 1:5){
  coll=c(13,17,19,21,23)
  nameo=c('Bd21-3','Bd30-1','Bd3-1','BdTR12c','Koz-3')
dmr.windows=subset(data,data[,coll[i]]>=3 #Bd1-1 read filter
                   & data[,15]>=3 #Bd21 read filter
                   & data[,coll[i]+1]>=2 #Bd1-1 site filter
                   & data[,16]>=2 #Bd21 site filter
                   & (data[,i+25] >=0.7 | data[,i+25]<= -0.7) #methylation difference filter
                   )
results[i+1,1]=nrow(dmr.windows)

group.id=c(1,rep(NA,nrow(dmr.windows)-1))
for(q in 2:nrow(dmr.windows)){
  group.id[q]=ifelse(dmr.windows[q,2] - dmr.windows[q-1,2]<=100 & dmr.windows[q,1]==dmr.windows[q-1,1] & dmr.windows[q,i+25] * dmr.windows[q-1,i+25] > 0,group.id[q-1],group.id[q-1]+1)
  }

dmr.windows=cbind(dmr.windows,group.id)

#dump out passing windows into files for later analysis
write.table(dmr.windows,paste(nameo[i],'.CG.dmr.windows.txt',sep=''),sep='\t',row.names=F,quote=F)

dmr.window.size=table(table(group.id))
#barplot(dmr.window.size,main='Number of sequential windows for each DMR',xlab='Number of windows (size = number * 100bp)',ylab='Frequency')

results[i+1,2]=sum(dmr.window.size[2:length(dmr.window.size)])
}
```

```
rownames(results)=c('Bd1-1','Bd21-3','Bd30-1','Bd3-1','BdTR12c','Koz-3')
colnames(results)=c('total dmr windows','200bp+ DMRs')
barplot(results[,1],beside=T,main='Total number of 100bp DMR windows\npassing thresholds')
```

```
barplot(results[,2],beside=T,main='Total number of 200bp+ DMR windows\npassing thresholds')
```

Similar relationship between lines and DMR number under both size filteres, however sub 10k is much easier to work with.

The **.dmr.windows.txt** files that were created will allow these filters to be applied. Files are moved to edmund to perform grouping of windows and bed file creation. Making bedfiles of DMRs passing the filters:

```
for FILE in *.txt
do
perl -pi -e '$_ = "" if ($. == 1);' $FILE
done

#NOTE the summary movement of columns 25-30. These are the contrast values for each set of DMRs and allows a hypo/hypermethylation call to be made
bedtools groupby -prec 15 -g 31 -c 1,2,3,25 -o first,min,max,first -i Bd1-1.CG.dmr.windows.txt > Bd1-1.CG.dmr.windows.txt.bed
bedtools groupby -prec 15 -g 31 -c 1,2,3,26 -o first,min,max,first -i Bd21-3.CG.dmr.windows.txt > Bd21-3.CG.dmr.windows.txt.bed
bedtools groupby -prec 15 -g 31 -c 1,2,3,27 -o first,min,max,first -i Bd30-1.CG.dmr.windows.txt > Bd30-1.CG.dmr.windows.txt.bed
bedtools groupby -prec 15 -g 31 -c 1,2,3,28 -o first,min,max,first -i Bd3-1.CG.dmr.windows.txt > Bd3-1.CG.dmr.windows.txt.bed
bedtools groupby -prec 15 -g 31 -c 1,2,3,29 -o first,min,max,first -i BdTR12c.CG.dmr.windows.txt > BdTR12c.CG.dmr.windows.txt.bed
bedtools groupby -prec 15 -g 31 -c 1,2,3,30 -o first,min,max,first -i Koz-3.CG.dmr.windows.txt > Koz-3.CG.dmr.windows.txt.bed


#then in R (still on edmund)

a=dir(pattern="*txt.bed")

for(i in 1:length(a)) {
temp=read.delim(a[i],head=F)
size=temp$V4 - temp$V3
type=ifelse(temp$V5 > 0, "hyper","hypo")
temp=cbind(temp,size,type)
temp=temp[,c(2,3,4,1,6,7)]
temp=cbind(temp,rep(unlist(strsplit(a[i],'.dmr'))[1],nrow(temp)))
colnames(temp)=c('chrom','start','stop','dmrid','size','type','contrast')

write.table(temp,paste(a[i],".bed",sep=''),sep='\t',row.names=F,quote=F,col.names=F)
temp.sub=subset(temp,temp$size > 99)
write.table(temp.sub,paste(a[i],".sizeflt.bed",sep=''),sep='\t',row.names=F,quote=F,col.names=F)
}
```

The final DMRs for analysis are in the **sizeflt.bed** files.

### NOW CHG

Read in the data and pull the columns of interest, also check the percentage levels compared to actual new met/total calculations (likely some rounding issues)

```
all=read.delim('7_geno_CHG_100bp_windows_all.txt',head=T)

f1=all[,5]/all[,7]
f2=all[,10]/all[,12]
f3=all[,15]/all[,17]
f4=all[,20]/all[,22]
f5=all[,25]/all[,27]
f6=all[,30]/all[,32]
f7=all[,35]/all[,37]

data=cbind(all[,1:3],f1,f2,f3,f4,f5,f6,f7,all[,c(7,8,12,13,17,18,22,23,27,28,32,33,37,38)])
colnames(data)[1:10]=c('chrom','start','stop','Bd1-1','Bd21-3','Bd21','Bd30-1','Bd3-1','BdTR12c','Koz-3')
```

Test calculate at different thresholds

```
f1f3=f1-f3
f2f3=f2-f3
f4f3=f4-f3
f5f3=f5-f3
f6f3=f6-f3
f7f3=f7-f3

data=cbind(data,f1f3,f2f3,f4f3,f5f3,f6f3,f7f3)
colnames(data)[25:30]=c('Bd1-1vsBd21','Bd21-3vsBd21','Bd30-1vsBd21','Bd3-1vsBd21','BdTR12cvsBd21','Koz-3vsBd21')
```

Just work with Bd1-1 vs Bd21 for now

```
test=subset(data,data[,11]>=1 & data[,15]>=1)
dim(test)
```

```
## [1] 2251031      30
```

**Looking at the number of windows based on read count filter (for both samples)**

```
coverage.prop=NULL
for(i in 1:15){
coverage.prop=c(coverage.prop,nrow(subset(test,test[,11]>=i & test[,15]>=i))/nrow(test))
}
```

```
prop=cbind(matrix(1:15,ncol=1),coverage.prop)
plot(prop, main="Proportion of CHG windows in which\n both samples have met coverage threshold",xlab='total read coverage')
```

**Looking at number of cytosines with coverage in both samples**

```
#note, this is dividing by two given CG sites are counted on both + and - strand
hist(test[,12]/2, main='Histogram of CHG cytosines in windows for Bd1-1',xlab='cytosine site count')
```

```
site.prop=NULL
for(i in 1:15){
site.prop=c(site.prop,nrow(subset(test,test[,12]>=i & test[,16]>=i))/nrow(test))
}
```

```
site=cbind(matrix(1:15,ncol=1),site.prop)
plot(site, main="Proportion of CHG windows in which\n both samples have met site count threshold",xlab='total cytosine count')
```

**Now for methylation difference between samples (weighted methylation count)**

```
hist(test[,25],breaks=200,main='CHG methylation difference between Bd1-1 and Bd21',xlab='methylation difference (Bd1-1 - Bd21)')
```

```
temp=subset(test,test[,25]!=0)
hist(temp[,25],breaks=200,main='CHG methylation difference between Bd1-1 and Bd21\n (0 difference removed)',xlab='methylation difference (Bd1-1 - Bd21)')
```

```
met.prop=NULL
for(i in c(.60,.70,.80,.90,.95)){
met.prop=c(met.prop,nrow(subset(test,test[,25]>=i | test[,25]<=-i))/nrow(test))
}
```

```
met=cbind(matrix(c(60,70,80,90,95),ncol=1),met.prop)
plot(met, main="Proportion of windows in which\n methylation difference is greater than",xlab='Difference in methylation (%)')
```

# So, throw them all together, and how many windows do we see…

```
dmr.windows=subset(data,data[,11]>=3 #Bd1-1 read filter
                   & data[,15]>=3 #Bd21 read filter
                   & data[,12]>=2 #Bd1-1 site filter
                   & data[,16]>=2 #Bd21 site filter
                   & (data[,25] >=0.5 | data[,25]<= -0.5) #methylation difference filter updated July2015
                   )

nrow(dmr.windows)
```

```
## [1] 60600
```

```
hist(dmr.windows[,25],breaks=200)
```

```
summary(dmr.windows[,25])
```

```
##    Min. 1st Qu.  Median    Mean 3rd Qu.    Max. 
## -1.0000 -0.7172 -0.5794 -0.3215  0.5000  1.0000
```

```
group.id=c(1,rep(NA,nrow(dmr.windows)-1))
for(q in 2:nrow(dmr.windows)){
  group.id[q]=ifelse(dmr.windows[q,2] - dmr.windows[q-1,2]<=100 & dmr.windows[q,1]==dmr.windows[q-1,1] & dmr.windows[q,25] * dmr.windows[q-1,25] > 0,group.id[q-1],group.id[q-1]+1)
  }

dmr.window.size=table(table(group.id))
barplot(dmr.window.size,main='Number of sequential windows for each CHG DMR',xlab='Number of windows (size = number * 100bp)',ylab='Frequency')
```

```
dmr.windows=cbind(dmr.windows,group.id)

write.table(dmr.windows,'Bd1-1.CHG.dmr.windows.txt',sep='\t',row.names=F,quote=F)
sum(dmr.window.size[2:length(dmr.window.size)])
```

```
## [1] 8396
```

```
#bd1-1
results=matrix(NA,ncol=2,nrow=6)
results[1,1]=nrow(dmr.windows)
results[1,2]=sum(dmr.window.size[2:length(dmr.window.size)])

#the rest
for(i in 1:5){
  coll=c(13,17,19,21,23)
  nameo=c('Bd21-3','Bd30-1','Bd3-1','BdTR12c','Koz-3')
dmr.windows=subset(data,data[,coll[i]]>=3 #Bd1-1 read filter
                   & data[,15]>=3 #Bd21 read filter
                   & data[,coll[i]+1]>=2 #Bd1-1 site filter
                   & data[,16]>=2 #Bd21 site filter
                   & (data[,i+25] >=0.5 | data[,i+25]<= -0.5) #methylation difference filter
                   )
results[i+1,1]=nrow(dmr.windows)

group.id=c(1,rep(NA,nrow(dmr.windows)-1))
for(q in 2:nrow(dmr.windows)){
  group.id[q]=ifelse(dmr.windows[q,2] - dmr.windows[q-1,2]<=100 & dmr.windows[q,1]==dmr.windows[q-1,1] & dmr.windows[q,i+25] * dmr.windows[q-1,i+25] > 0,group.id[q-1],group.id[q-1]+1)
  }

dmr.windows=cbind(dmr.windows,group.id)

write.table(dmr.windows,paste(nameo[i],'.CHG.dmr.windows.txt',sep=''),sep='\t',row.names=F,quote=F)

dmr.window.size=table(table(group.id))
#barplot(dmr.window.size,main='Number of sequential windows for each CHG DMR',xlab='Number of windows (size = number * 100bp)',ylab='Frequency')

results[i+1,2]=sum(dmr.window.size[2:length(dmr.window.size)])
}
```

```
rownames(results)=c('Bd1-1','Bd21-3','Bd30-1','Bd3-1','BdTR12c','Koz-3')
colnames(results)=c('total dmr windows','200bp+ DMRs')
barplot(results[,1],beside=T,main='Total number of 100bp CHG DMR windows\npassing thresholds')
```

```
barplot(results[,2],beside=T,main='Total number of 200bp+ CHG DMR windows\npassing thresholds')
```

So although fewer DMRs in total, CHG displays very similar characteristics to CG DMRs. I will use the same filters for both CG and CHG methylation.

The **.dmr.windows.txt** files that were created will allow these filters to be applied. Files are moved to edmund to perform grouping of windows and bed file creation. Making bedfiles of DMRs passing the filters:

```
for FILE in *.txt
do
perl -pi -e '$_ = "" if ($. == 1);' $FILE
done

#NOTE the summary movement of columns 25-30. These are the contrast values for each set of DMRs and allows a hypo/hypermethylation call to be made
bedtools groupby -prec 15 -g 31 -c 1,2,3,25 -o first,min,max,first -i Bd1-1.CHG.dmr.windows.txt > Bd1-1.CHG.dmr.windows.txt.bed
bedtools groupby -prec 15 -g 31 -c 1,2,3,26 -o first,min,max,first -i Bd21-3.CHG.dmr.windows.txt > Bd21-3.CHG.dmr.windows.txt.bed
bedtools groupby -prec 15 -g 31 -c 1,2,3,27 -o first,min,max,first -i Bd30-1.CHG.dmr.windows.txt > Bd30-1.CHG.dmr.windows.txt.bed
bedtools groupby -prec 15 -g 31 -c 1,2,3,28 -o first,min,max,first -i Bd3-1.CHG.dmr.windows.txt > Bd3-1.CHG.dmr.windows.txt.bed
bedtools groupby -prec 15 -g 31 -c 1,2,3,29 -o first,min,max,first -i BdTR12c.CHG.dmr.windows.txt > BdTR12c.CHG.dmr.windows.txt.bed
bedtools groupby -prec 15 -g 31 -c 1,2,3,30 -o first,min,max,first -i Koz-3.CHG.dmr.windows.txt > Koz-3.CHG.dmr.windows.txt.bed


#then in R (still on edmund)

a=dir(pattern="*txt.bed")

for(i in 1:length(a)) {
temp=read.delim(a[i],head=F)
size=temp$V4 - temp$V3
type=ifelse(temp$V5 > 0, "hyper","hypo")
temp=cbind(temp,size,type)
temp=temp[,c(2,3,4,1,6,7)]
temp=cbind(temp,rep(unlist(strsplit(a[i],'.dmr'))[1],nrow(temp)))
colnames(temp)=c('chrom','start','stop','dmrid','size','type','contrast')

write.table(temp,paste(a[i],".bed",sep=''),sep='\t',row.names=F,quote=F,col.names=F)
temp.sub=subset(temp,temp$size > 99)
write.table(temp.sub,paste(a[i],".sizeflt.bed",sep=''),sep='\t',row.names=F,quote=F,col.names=F)
}
```

The final DMRs for analysis are in the **sizeflt.bed** files.

# CHH Methylation and DMRs…

CHH methylation is a comletely different beast. As an order of magnitude less (0-5% on average), it requires very different filters to identify differential methylation. Let’s investigate by loading in the data in a similar fashion:``{r, cache=TRUE}

```
all=read.delim('7_geno_CHH_100bp_windows_all.txt',head=T)

names(all)
```

```
##  [1] "V1"                 "V2"                 "V3"                
##  [4] "Bd1.1_percentage"   "Bd1.1_met"          "Bd1.1_unmet"       
##  [7] "Bd1.1_total"        "Bd1.1_sitecount"    "Bd21.3_percentage" 
## [10] "Bd21.3_met"         "Bd21.3_unmet"       "Bd21.3_total"      
## [13] "Bd21.3_sitecount"   "Bd21_percentage"    "Bd21_met"          
## [16] "Bd21_unmet"         "Bd21_total"         "Bd21_sitecount"    
## [19] "Bd30.1_percentage"  "Bd30.1_met"         "Bd30.1_unmet"      
## [22] "Bd30.1_total"       "Bd30.1_sitecount"   "Bd3.1_percentage"  
## [25] "Bd3.1_met"          "Bd3.1_unmet"        "Bd3.1_total"       
## [28] "Bd3.1_sitecount"    "BdTR12c_percentage" "BdTR12c_met"       
## [31] "BdTR12c_unmet"      "BdTR12c_total"      "BdTR12c_sitecount" 
## [34] "Koz.3_percentage"   "Koz.3_met"          "Koz.3_unmet"       
## [37] "Koz.3_total"        "Koz.3_sitecount"
```

```
f1=all[,5]/all[,7]
f2=all[,10]/all[,12]
f3=all[,15]/all[,17]
f4=all[,20]/all[,22]
f5=all[,25]/all[,27]
f6=all[,30]/all[,32]
f7=all[,35]/all[,37]

data=cbind(all[,1:3],f1,f2,f3,f4,f5,f6,f7,all[,c(7,8,12,13,17,18,22,23,27,28,32,33,37,38)])
colnames(data)[1:10]=c('chrom','start','stop','Bd1-1','Bd21-3','Bd21','Bd30-1','Bd3-1','BdTR12c','Koz-3')
```

We will also need to have the ‘contrast’ values. These are just the diverse lines methylation percentage minus the Bd21 (control) methylation percentage:

```
f1f3=f1-f3
f2f3=f2-f3
f4f3=f4-f3
f5f3=f5-f3
f6f3=f6-f3
f7f3=f7-f3

data=cbind(data,f1f3,f2f3,f4f3,f5f3,f6f3,f7f3)
colnames(data)[25:30]=c('Bd1-1vsBd21','Bd21-3vsBd21','Bd30-1vsBd21','Bd3-1vsBd21','BdTR12cvsBd21','Koz-3vsBd21')
```

To start, we will better understand what thresholds are important by working with Bd1-1 vs Bd21 for now. Therefore we can subset all windows to those that have *any* data in Bd1-1 and Bd21:

```
test=subset(data,data[,11]>=1 & data[,15]>=1)
dim(test)
```

```
## [1] 2430387      30
```

```
hist(test[,4],breaks=100,main='Bd1-1 CHH methylation histogram',xlab='Proportion methylated')
```

```
hist(test[,25],breaks=200)
```

**Looking at the number of windows based on read count filter (for both samples)**

```
coverage.prop=NULL
for(i in 1:15){
coverage.prop=c(coverage.prop,nrow(subset(test,test[,11]>=i & test[,15]>=i))/nrow(test))
}
```

```
prop=cbind(matrix(1:15,ncol=1),coverage.prop)
plot(prop, main="Proportion of CHG windows in which\n both samples have met coverage threshold",xlab='total read coverage')
```

A very similar trend regarding coverage. Having 3+ reads likely will work here as well.

**Looking at number of cytosines with coverage in both samples**

```
#note, this is dividing by two given CG sites are counted on both + and - strand
hist(test[,12], main='Histogram of CHH cytosines in windows for Bd1-1',xlab='cytosine site count')
```

The number of cytosines i nthe CHH context is much more centered with many cytosines.

```
site.prop=NULL
for(i in 1:15){
site.prop=c(site.prop,nrow(subset(test,test[,12]>=i & test[,16]>=i))/nrow(test))
}
```

```
site=cbind(matrix(1:15,ncol=1),site.prop)
plot(site, main="Proportion of CHH windows in which\n both samples have met site count threshold",xlab='total cytosine count')
```

A major difference compared to CG/CHG windows is that the increased number of cytosines does not lead to a strong dropoff of windows passing thresholds. Where before we had major drops after a count of 2, we could reach the same threshold of windows passing at a requirement of 8+ cytosines. Let’s go with that.

**Now for methylation difference between samples (weighted methylation count)**

```
hist(test[,25],breaks=200,main='CHH methylation difference between Bd1-1 and Bd21',xlab='methylation difference (Bd1-1 - Bd21)')
```

```
temp=subset(test,test[,25]!=0)
hist(temp[,25],breaks=200,main='CHH methylation difference between Bd1-1 and Bd21\n (0 difference removed)',xlab='methylation difference (Bd1-1 - Bd21)')
```

In this case, when we get rid of the exact no-difference windows (0), we still see that the majority of differences are less than 20% in either direction. This requires a significant change of methylation difference filter as 60%+ will discard almost everything in the dataset.

```
met.prop=NULL
for(i in c(.10,.20,.30,.40)){
met.prop=c(met.prop,nrow(subset(test,test[,25]>=i | test[,25]<=-i))/nrow(test))
}
```

```
met=cbind(matrix(c(10,20,30,40),ncol=1),met.prop)
plot(met, main="Proportion of windows in which\n methylation difference is greater than",xlab='Difference in methylation (%)')
```

So we may want a difference of 20-30%, however this does require that we be more carefuly regarding filtering based on methyation prop per sample. Otherwise the (albeit rare) 80-100 or 40-60 differences would pass, when they really should not:

```
bad.method=subset(temp,temp[,25] >= 0.20 | temp[,25] <= -0.20)
dim(bad.method)
```

```
## [1] 50147    30
```

```
hist(bad.method[,4],breaks=100,main='Bd1-1 methylation in DMRs')
```

```
hist(bad.method[,6],breaks=100,main='Bd21 methyation in DMRs')
```

```
hist(bad.method[,25],breaks=100,main='Bd1-1 - Bd21 methylation')
```

```
plot(bad.method[,4],bad.method[,6],main='Methylation scatter plot for bad method CHH DMRs',ylab='Bd21',xlab='Bd1-1')
```

As shown, with this basic method. You do not really filter to CHH states that are ‘on’ or ‘off’ between lines. There are many that are ‘more on’ or ‘more off’ which are very ambiguous at the moment. Instead, we can filter to force one sample to be low and one to be high:

```
better.method=subset(test,(test[,4] >= 0.25 & test[,6] <= 0.05) | (test[,4] <= 0.05 & test[,6] >= 0.25))
dim(better.method)
```

```
## [1] 24040    30
```

```
hist(better.method[,4],breaks=100,main='Bd1-1 methylation in DMRs')
```

```
hist(better.method[,6],breaks=100,main='Bd21 methyation in DMRs')
```

```
hist(better.method[,25],breaks=100,main='Bd1-1 - Bd21 methylation')
```

```
plot(better.method[,4],better.method[,6],main='Methylation scatter plot for good method CHH DMRs',ylab='Bd21',xlab='Bd1-1')
```

When we require that one sample has ‘low’ CHH (<5%) and the other sample has ‘high’ (>25%), we eliminate the issues seen in the first filter method. From this, we have identified

```
dmr.windows=subset(data,data[,11]>=3 #Bd1-1 read filter
                   & data[,15]>=3 #Bd21 read filter
                   & data[,12]>=8 #Bd1-1 site filter (CHH specific levels)
                   & data[,16]>=8 #Bd21 site filter (CHH specific levels)
                   & ((data[,4] >= 0.20 & data[,6] <= 0.05) | (data[,4] <= 0.05 & data[,6] >= 0.20)) #methylation difference filter
                   )

nrow(dmr.windows)
```

```
## [1] 16605
```

```
hist(dmr.windows[,25],breaks=200)
```

```
summary(dmr.windows[,25])
```

```
##    Min. 1st Qu.  Median    Mean 3rd Qu.    Max. 
## -1.0000 -0.3095 -0.2192 -0.1157  0.2105  1.0000
```

So about 10.6k windows passing the filters. Are there any neighboring windows ?

```
group.id=c(1,rep(NA,nrow(dmr.windows)-1))
for(q in 2:nrow(dmr.windows)){
  group.id[q]=ifelse(dmr.windows[q,2] - dmr.windows[q-1,2]<=100 & dmr.windows[q,1]==dmr.windows[q-1,1] & dmr.windows[q,25] * dmr.windows[q-1,25] > 0,group.id[q-1],group.id[q-1]+1)
  }

dmr.window.size=table(table(group.id))
barplot(dmr.window.size,main='Number of sequential windows for each CHH DMR',xlab='Number of windows (size = number * 100bp)',ylab='Frequency')
```

```
dmr.windows=cbind(dmr.windows,group.id)

write.table(dmr.windows,'Bd1-1.CHH.dmr.windows.txt',sep='\t',row.names=F,quote=F)
sum(dmr.window.size[2:length(dmr.window.size)])
```

```
## [1] 921
```

I am not sure how far I want to go in regards to CHH methylation in this manuscript. I may just report the levels, then focus on CG and CHG methylation differences.

Make and write out CHH dmr files:

```
#bd1-1
results=matrix(NA,ncol=2,nrow=6)
results[1,1]=nrow(dmr.windows)
results[1,2]=sum(dmr.window.size[2:length(dmr.window.size)])

#the rest
for(i in 1:5){
  coll=c(13,17,19,21,23)
  col2=c(5,7,8,9,10)
  nameo=c('Bd21-3','Bd30-1','Bd3-1','BdTR12c','Koz-3')
dmr.windows=subset(data,data[,coll[i]]>=3 #Bd1-1 read filter
                   & data[,15]>=3 #Bd21 read filter
                   & data[,coll[i]+1]>=8 #Bd1-1 site filter
                   & data[,16]>=8 #Bd21 site filter
                   & ((data[,col2[i]] >= 0.20 & data[,6] <= 0.05) | (data[,col2[i]] <= 0.05 & data[,6] >= 0.20)) #methylation difference filter
                   )
results[i+1,1]=nrow(dmr.windows)

group.id=c(1,rep(NA,nrow(dmr.windows)-1))
for(q in 2:nrow(dmr.windows)){
  group.id[q]=ifelse(dmr.windows[q,2] - dmr.windows[q-1,2]<=100 & dmr.windows[q,1]==dmr.windows[q-1,1] & dmr.windows[q,i+25] * dmr.windows[q-1,i+25] > 0,group.id[q-1],group.id[q-1]+1)
  }

dmr.windows=cbind(dmr.windows,group.id)

write.table(dmr.windows,paste(nameo[i],'.CHH.dmr.windows.txt',sep=''),sep='\t',row.names=F,quote=F)

dmr.window.size=table(table(group.id))
#barplot(dmr.window.size,main='Number of sequential windows for each CHG DMR',xlab='Number of windows (size = number * 100bp)',ylab='Frequency')

results[i+1,2]=sum(dmr.window.size[2:length(dmr.window.size)])
}
```

```
rownames(results)=c('Bd1-1','Bd21-3','Bd30-1','Bd3-1','BdTR12c','Koz-3')
colnames(results)=c('total dmr windows','200bp+ DMRs')
barplot(results[,1],beside=T,main='Total number of 100bp CHH DMR windows\npassing thresholds')
```

```
barplot(results[,2],beside=T,main='Total number of 200bp+ CHH DMR windows\npassing thresholds')
```

So 260-500 CHH dmrs per line… Not too bad.

The **.dmr.windows.txt** files that were created will allow these filters to be applied. Files are moved to edmund to perform grouping of windows and bed file creation. Making bedfiles of DMRs passing the filters:

```
for FILE in *.txt
do
perl -pi -e '$_ = "" if ($. == 1);' $FILE
done

#NOTE the summary movement of columns 25-30. These are the contrast values for each set of DMRs and allows a hypo/hypermethylation call to be made
bedtools groupby -prec 15 -g 31 -c 1,2,3,25 -o first,min,max,first -i Bd1-1.CHH.dmr.windows.txt > Bd1-1.CHH.dmr.windows.txt.bed
bedtools groupby -prec 15 -g 31 -c 1,2,3,26 -o first,min,max,first -i Bd21-3.CHH.dmr.windows.txt > Bd21-3.CHH.dmr.windows.txt.bed
bedtools groupby -prec 15 -g 31 -c 1,2,3,27 -o first,min,max,first -i Bd30-1.CHH.dmr.windows.txt > Bd30-1.CHH.dmr.windows.txt.bed
bedtools groupby -prec 15 -g 31 -c 1,2,3,28 -o first,min,max,first -i Bd3-1.CHH.dmr.windows.txt > Bd3-1.CHH.dmr.windows.txt.bed
bedtools groupby -prec 15 -g 31 -c 1,2,3,29 -o first,min,max,first -i BdTR12c.CHH.dmr.windows.txt > BdTR12c.CHH.dmr.windows.txt.bed
bedtools groupby -prec 15 -g 31 -c 1,2,3,30 -o first,min,max,first -i Koz-3.CHH.dmr.windows.txt > Koz-3.CHH.dmr.windows.txt.bed


#then in R (still on edmund)

a=dir(pattern="*txt.bed")

for(i in 1:length(a)) {
temp=read.delim(a[i],head=F)
size=temp$V4 - temp$V3
type=ifelse(temp$V5 > 0, "hyper","hypo")
temp=cbind(temp,size,type)
temp=temp[,c(2,3,4,1,6,7)]
temp=cbind(temp,rep(unlist(strsplit(a[i],'.dmr'))[1],nrow(temp)))
colnames(temp)=c('chrom','start','stop','dmrid','size','type','contrast')

write.table(temp,paste(a[i],".bed",sep=''),sep='\t',row.names=F,quote=F,col.names=F)
temp.sub=subset(temp,temp$size > 99)
write.table(temp.sub,paste(a[i],".sizeflt.bed",sep=''),sep='\t',row.names=F,quote=F,col.names=F)
}
```

The final DMRs for analysis are in the **sizeflt.bed** files.
