# Supplementary material for: DNA methylation profiles of diverse Brachypodium distachyon align with underlying genetic diversity
Source: Genome Res. 2016 Nov;26(11):1520–31. doi: 10.1101/gr.205468.116 (PMC5088594; doi:10.1101/gr.205468.116)
Supplement: Supplemental Material [file supp_gr.205468.116_Supplemental_Data_1.zip › FIGURE2_DMRtiles_to_genomic_tiles.html]

DMR tiles vs genomic tiles


# DMR tiles vs genomic tiles

#### *SRE*

#### *29 July 2015*

Checking things with Bd1-1 CG DMRs at first

```
#grab old file

all=read.delim('../../1. Bd21_analysis/20150724_all_tile_methylation_percentages/Bd21_total_tile_annotation.txt',head=T)
```

```
library(reshape2)
bd1.cg.dmr=read.delim('CGDMRS/Bd1-1.CG.dmr.windows.txt.bed',head=F)
bd1.cg.dmr=bd1.cg.dmr[,2:5]
colnames(bd1.cg.dmr)=c('V1','V2','V3','V4')

all.test=merge(all,bd1.cg.dmr,by=c('V1','V2','V3'))
cg.dmr.type=ifelse(all.test$V4>0,'hyper','hypo')
all.test=cbind(all.test,cg.dmr.type)
barplot(table(all.test$cg.dmr.type,all.test$window_class),beside=T,las=2,ylim=c(0,10000))
```

```
#legend('topright',c('hyper','hypo'),col=c('black','grey'),lty=1,lwd=5)
a=table(all.test$cg.dmr.type,all.test$window_class)

table1=matrix(NA,ncol=14,nrow=1)
temp=table(all.test$anno.class)
table1[,1:14]=table(all.test$anno.class)
colnames(table1)=names(temp)
table1=table1[,c(7,8,13,14,1,2,3,4,5,6,9,10,11,12)]
barplot(table1,las=2,col='black',ylim=c(0,12000))
```

```
table2=table(all.test$cg.dmr.type,all.test$anno.class)
table2=table2[,c(7,8,13,14,1,2,3,4,5,6,9,10,11,12)]
barplot(prop.table(table2,margin=2),las=2)
```

```
bd1.chg.dmr=read.delim('CHGDMRS/Bd1-1.CHG.dmr.windows.txt.bed',head=F)
bd1.chg.dmr=bd1.chg.dmr[,2:5]
colnames(bd1.chg.dmr)=c('V1','V2','V3','V4')
all.test=merge(all,bd1.chg.dmr,by=c('V1','V2','V3'))
chg.dmr.type=ifelse(all.test$V4>0,'hyper','hypo')
all.test=cbind(all.test,chg.dmr.type)
barplot(table(all.test$chg.dmr.type,all.test$window_class),beside=T,las=2,ylim=c(0,25000))
```

```
#legend('topright',c('hyper','hypo'),col=c('black','grey'),lty=1,lwd=5)
b=table(all.test$chg.dmr.type,all.test$window_class)

table1=matrix(NA,ncol=14,nrow=1)
temp=table(all.test$anno.class)
table1[,1:14]=table(all.test$anno.class)
colnames(table1)=names(temp)
table1=table1[,c(7,8,13,14,1,2,3,4,5,6,9,10,11,12)]
barplot(table1,las=2,col='black',ylim=c(0,20000))
```

```
table2=table(all.test$chg.dmr.type,all.test$anno.class)
table2=table2[,c(7,8,13,14,1,2,3,4,5,6,9,10,11,12)]
barplot(prop.table(table2,margin=2),las=2)
```

```
bd1.chh.dmr=read.delim('CHHDMRS/Bd1-1.CHH.dmr.windows.txt.bed',head=F)
bd1.chh.dmr=bd1.chh.dmr[,2:5]
colnames(bd1.chh.dmr)=c('V1','V2','V3','V4')
all.test=merge(all,bd1.chh.dmr,by=c('V1','V2','V3'))
chh.dmr.type=ifelse(all.test$V4>0,'hyper','hypo')
all.test=cbind(all.test,chh.dmr.type)
barplot(table(all.test$chh.dmr.type,all.test$window_class),beside=T,las=2,ylim=c(0,6000))
```

```
#legend('topright',c('hyper','hypo'),col=c('black','grey'),lty=1,lwd=5)

c=table(all.test$chh.dmr.type,all.test$window_class)
table1=matrix(NA,ncol=14,nrow=1)
temp=table(all.test$anno.class)
table1[,1:14]=table(all.test$anno.class)
colnames(table1)=names(temp)
table1=table1[,c(7,8,13,14,1,2,3,4,5,6,9,10,11,12)]
barplot(table1,las=2,col='black',ylim=c(0,6000))
```

```
table2=table(all.test$chh.dmr.type,all.test$anno.class)
table2=table2[,c(7,8,13,14,1,2,3,4,5,6,9,10,11,12)]
barplot(prop.table(table2,margin=2),las=2)
```

```
a1=melt(a)
a1=cbind(a1,rep('CGDMR',nrow(a1)))
colnames(a1)[4]='type'
b1=melt(b)
b1=cbind(b1,rep('CHGDMR',nrow(b1)))
colnames(b1)[4]='type'
c1=melt(c)
c1=cbind(c1,rep('CHHDMR',nrow(c1)))
colnames(c1)[4]='type'

d=rbind(a1,b1,c1)
d$type=paste(d$type,d$Var1,sep='_')
pp=dcast(d,Var2 ~ type, value.var='value')
rownames(pp)=pp$Var2
pp=pp[,2:7]
par(mar=c(5,4,4,8),xpd=T)
barplot(prop.table(t(pp),margin=2),col=c('#990000','#C26666','#003399','#99ADD6','#336600','#ADC299'),las=2)
legend('topright',inset=c(-.25,0),fill=c('#990000','#C26666','#003399','#99ADD6','#336600','#ADC299'),legend=rownames(prop.table(t(pp),margin=2)),bty='n')
```

```
bd1.cg.dmr=read.delim('CGDMRS/Bd3-1.CG.dmr.windows.txt.bed',head=F)
bd1.cg.dmr=bd1.cg.dmr[,2:5]
colnames(bd1.cg.dmr)=c('V1','V2','V3','V4')

all.test=merge(all,bd1.cg.dmr,by=c('V1','V2','V3'))
cg.dmr.type=ifelse(all.test$V4>0,'hyper','hypo')
all.test=cbind(all.test,cg.dmr.type)
barplot(table(all.test$cg.dmr.type,all.test$window_class),beside=T,las=2)
legend('topright',c('hyper','hypo'),col=c('black','grey'),lty=1,lwd=5)
```

```
bd1.cg.dmr=read.delim('CGDMRS/Bd21-3.CG.dmr.windows.txt.bed',head=F)
bd1.cg.dmr=bd1.cg.dmr[,2:5]
colnames(bd1.cg.dmr)=c('V1','V2','V3','V4')

all.test=merge(all,bd1.cg.dmr,by=c('V1','V2','V3'))
cg.dmr.type=ifelse(all.test$V4>0,'hyper','hypo')
all.test=cbind(all.test,cg.dmr.type)
barplot(table(all.test$cg.dmr.type,all.test$window_class),beside=T,las=2)
legend('topright',c('hyper','hypo'),col=c('black','grey'),lty=1,lwd=5)
```

```
bd1.cg.dmr=read.delim('CGDMRS/Bd30-1.CG.dmr.windows.txt.bed',head=F)
bd1.cg.dmr=bd1.cg.dmr[,2:5]
colnames(bd1.cg.dmr)=c('V1','V2','V3','V4')

all.test=merge(all,bd1.cg.dmr,by=c('V1','V2','V3'))
cg.dmr.type=ifelse(all.test$V4>0,'hyper','hypo')
all.test=cbind(all.test,cg.dmr.type)
barplot(table(all.test$cg.dmr.type,all.test$window_class),beside=T,las=2)
legend('topright',c('hyper','hypo'),col=c('black','grey'),lty=1,lwd=5)
```

```
bd1.cg.dmr=read.delim('CGDMRS/BdTR12c.CG.dmr.windows.txt.bed',head=F)
bd1.cg.dmr=bd1.cg.dmr[,2:5]
colnames(bd1.cg.dmr)=c('V1','V2','V3','V4')

all.test=merge(all,bd1.cg.dmr,by=c('V1','V2','V3'))
cg.dmr.type=ifelse(all.test$V4>0,'hyper','hypo')
all.test=cbind(all.test,cg.dmr.type)
barplot(table(all.test$cg.dmr.type,all.test$window_class),beside=T,las=2)
legend('topright',c('hyper','hypo'),col=c('black','grey'),lty=1,lwd=5)
```

```
bd1.cg.dmr=read.delim('CGDMRS/Koz-3.CG.dmr.windows.txt.bed',head=F)
bd1.cg.dmr=bd1.cg.dmr[,2:5]
colnames(bd1.cg.dmr)=c('V1','V2','V3','V4')

all.test=merge(all,bd1.cg.dmr,by=c('V1','V2','V3'))
cg.dmr.type=ifelse(all.test$V4>0,'hyper','hypo')
all.test=cbind(all.test,cg.dmr.type)
barplot(table(all.test$cg.dmr.type,all.test$window_class),beside=T,las=2)
legend('topright',c('hyper','hypo'),col=c('black','grey'),lty=1,lwd=5)
```

Majority of CG hypermethylation events come from tiles previously having no methylation of any kind. Loss of methylation is most common from CG-only regions as well as CG-CHG

```
bd1.chg.dmr=read.delim('CHGDMRS/Bd1-1.CHG.dmr.windows.txt.bed',head=F)
bd1.chg.dmr=bd1.chg.dmr[,2:5]
colnames(bd1.chg.dmr)=c('V1','V2','V3','V4')
all.test=merge(all,bd1.chg.dmr,by=c('V1','V2','V3'))
chg.dmr.type=ifelse(all.test$V4>0,'hyper','hypo')
all.test=cbind(all.test,chg.dmr.type)
barplot(table(all.test$chg.dmr.type,all.test$window_class),beside=T,las=2)
legend('topright',c('hyper','hypo'),col=c('black','grey'),lty=1,lwd=5)
```

```
bd1.chg.dmr=read.delim('CHGDMRS/Bd3-1.CHG.dmr.windows.txt.bed',head=F)
bd1.chg.dmr=bd1.chg.dmr[,2:5]
colnames(bd1.chg.dmr)=c('V1','V2','V3','V4')
all.test=merge(all,bd1.chg.dmr,by=c('V1','V2','V3'))
chg.dmr.type=ifelse(all.test$V4>0,'hyper','hypo')
all.test=cbind(all.test,chg.dmr.type)
barplot(table(all.test$chg.dmr.type,all.test$window_class),beside=T,las=2)
legend('topright',c('hyper','hypo'),col=c('black','grey'),lty=1,lwd=5)
```

```
bd1.chg.dmr=read.delim('CHGDMRS/Bd21-3.CHG.dmr.windows.txt.bed',head=F)
bd1.chg.dmr=bd1.chg.dmr[,2:5]
colnames(bd1.chg.dmr)=c('V1','V2','V3','V4')
all.test=merge(all,bd1.chg.dmr,by=c('V1','V2','V3'))
chg.dmr.type=ifelse(all.test$V4>0,'hyper','hypo')
all.test=cbind(all.test,chg.dmr.type)
barplot(table(all.test$chg.dmr.type,all.test$window_class),beside=T,las=2)
legend('topright',c('hyper','hypo'),col=c('black','grey'),lty=1,lwd=5)
```

```
bd1.chg.dmr=read.delim('CHGDMRS/Bd30-1.CHG.dmr.windows.txt.bed',head=F)
bd1.chg.dmr=bd1.chg.dmr[,2:5]
colnames(bd1.chg.dmr)=c('V1','V2','V3','V4')
all.test=merge(all,bd1.chg.dmr,by=c('V1','V2','V3'))
chg.dmr.type=ifelse(all.test$V4>0,'hyper','hypo')
all.test=cbind(all.test,chg.dmr.type)
barplot(table(all.test$chg.dmr.type,all.test$window_class),beside=T,las=2)
legend('topright',c('hyper','hypo'),col=c('black','grey'),lty=1,lwd=5)
```

```
bd1.chg.dmr=read.delim('CHGDMRS/BdTR12c.CHG.dmr.windows.txt.bed',head=F)
bd1.chg.dmr=bd1.chg.dmr[,2:5]
colnames(bd1.chg.dmr)=c('V1','V2','V3','V4')
all.test=merge(all,bd1.chg.dmr,by=c('V1','V2','V3'))
chg.dmr.type=ifelse(all.test$V4>0,'hyper','hypo')
all.test=cbind(all.test,chg.dmr.type)
barplot(table(all.test$chg.dmr.type,all.test$window_class),beside=T,las=2)
legend('topright',c('hyper','hypo'),col=c('black','grey'),lty=1,lwd=5)
```

```
bd1.chg.dmr=read.delim('CHGDMRS/Koz-3.CHG.dmr.windows.txt.bed',head=F)
bd1.chg.dmr=bd1.chg.dmr[,2:5]
colnames(bd1.chg.dmr)=c('V1','V2','V3','V4')
all.test=merge(all,bd1.chg.dmr,by=c('V1','V2','V3'))
chg.dmr.type=ifelse(all.test$V4>0,'hyper','hypo')
all.test=cbind(all.test,chg.dmr.type)
barplot(table(all.test$chg.dmr.type,all.test$window_class),beside=T,las=2)
legend('topright',c('hyper','hypo'),col=c('black','grey'),lty=1,lwd=5)
```

The vast majority of CHG loses appear in regions that were previously cg\_chg methylated. Gains in CHG ethylation are most common in cg-only regions

Now CHH:

```
bd1.chh.dmr=read.delim('CHHDMRS/Bd1-1.CHH.dmr.windows.txt.bed',head=F)
bd1.chh.dmr=bd1.chh.dmr[,2:5]
colnames(bd1.chh.dmr)=c('V1','V2','V3','V4')
all.test=merge(all,bd1.chh.dmr,by=c('V1','V2','V3'))
chh.dmr.type=ifelse(all.test$V4>0,'hyper','hypo')
all.test=cbind(all.test,chh.dmr.type)
barplot(table(all.test$chh.dmr.type,all.test$window_class),beside=T,las=2)
legend('topright',c('hyper','hypo'),col=c('black','grey'),lty=1,lwd=5)
```

```
bd1.chh.dmr=read.delim('CHHDMRS/Bd3-1.CHH.dmr.windows.txt.bed',head=F)
bd1.chh.dmr=bd1.chh.dmr[,2:5]
colnames(bd1.chh.dmr)=c('V1','V2','V3','V4')
all.test=merge(all,bd1.chh.dmr,by=c('V1','V2','V3'))
chh.dmr.type=ifelse(all.test$V4>0,'hyper','hypo')
all.test=cbind(all.test,chh.dmr.type)
barplot(table(all.test$chh.dmr.type,all.test$window_class),beside=T,las=2)
legend('topright',c('hyper','hypo'),col=c('black','grey'),lty=1,lwd=5)
```

```
bd1.chh.dmr=read.delim('CHHDMRS/Bd21-3.CHH.dmr.windows.txt.bed',head=F)
bd1.chh.dmr=bd1.chh.dmr[,2:5]
colnames(bd1.chh.dmr)=c('V1','V2','V3','V4')
all.test=merge(all,bd1.chh.dmr,by=c('V1','V2','V3'))
chh.dmr.type=ifelse(all.test$V4>0,'hyper','hypo')
all.test=cbind(all.test,chh.dmr.type)
barplot(table(all.test$chh.dmr.type,all.test$window_class),beside=T,las=2)
legend('topright',c('hyper','hypo'),col=c('black','grey'),lty=1,lwd=5)
```

```
bd1.chh.dmr=read.delim('CHHDMRS/Bd30-1.CHH.dmr.windows.txt.bed',head=F)
bd1.chh.dmr=bd1.chh.dmr[,2:5]
colnames(bd1.chh.dmr)=c('V1','V2','V3','V4')
all.test=merge(all,bd1.chh.dmr,by=c('V1','V2','V3'))
chh.dmr.type=ifelse(all.test$V4>0,'hyper','hypo')
all.test=cbind(all.test,chh.dmr.type)
barplot(table(all.test$chh.dmr.type,all.test$window_class),beside=T,las=2)
legend('topright',c('hyper','hypo'),col=c('black','grey'),lty=1,lwd=5)
```

```
bd1.chh.dmr=read.delim('CHHDMRS/BdTR12c.CHH.dmr.windows.txt.bed',head=F)
bd1.chh.dmr=bd1.chh.dmr[,2:5]
colnames(bd1.chh.dmr)=c('V1','V2','V3','V4')
all.test=merge(all,bd1.chh.dmr,by=c('V1','V2','V3'))
chh.dmr.type=ifelse(all.test$V4>0,'hyper','hypo')
all.test=cbind(all.test,chh.dmr.type)
barplot(table(all.test$chh.dmr.type,all.test$window_class),beside=T,las=2)
legend('topright',c('hyper','hypo'),col=c('black','grey'),lty=1,lwd=5)
```

```
bd1.chh.dmr=read.delim('CHHDMRS/Koz-3.CHH.dmr.windows.txt.bed',head=F)
bd1.chh.dmr=bd1.chh.dmr[,2:5]
colnames(bd1.chh.dmr)=c('V1','V2','V3','V4')
all.test=merge(all,bd1.chh.dmr,by=c('V1','V2','V3'))
chh.dmr.type=ifelse(all.test$V4>0,'hyper','hypo')
all.test=cbind(all.test,chh.dmr.type)
barplot(table(all.test$chh.dmr.type,all.test$window_class),beside=T,las=2)
legend('topright',c('hyper','hypo'),col=c('black','grey'),lty=1,lwd=5)
```

CHH DMR tiles show a very different pattern compared to the other contexts. the majority of CHH loses appear from tiles previously methylated in all contexts. Gains in CHH are most common in tiles that already have cg and chg methylation.

Note, all these need to be copared to the overall number of tiles in each catagory if we really want to find an enrichment:

```
barplot(table(all$window_class),las=2)
```

## Now, do look at all 6 diverse inbreds to see if these are conserved patterns

```
library(reshape2)
out=NULL
for(i in 1:6){
  samples=c('Bd1-1','Bd3-1','Bd21-3','Bd30-1','BdTR12c','Koz-3')
  input=read.delim(paste('CGDMRS/',samples[i],'.CG.dmr.windows.txt.bed',sep=''),head=F)
  dmr.set=input[,2:5]
  colnames(dmr.set)=c('V1','V2','V3','V4')
  dmr.set$V4=ifelse(dmr.set$V4>0,'hyper','hypo')
  colnames(dmr.set)[4]=samples[i]
  all.test=merge(all,dmr.set,by=c('V1','V2','V3'))
  values=melt(table(all.test[,ncol(all.test)],all.test$window_class))
  values=cbind(values,rep(samples[i],nrow(values)))
  out=rbind(out,values)
}
```
