# Supplementary material for: DNA methylation profiles of diverse Brachypodium distachyon align with underlying genetic diversity
Source: Genome Res. 2016 Nov;26(11):1520–31. doi: 10.1101/gr.205468.116 (PMC5088594; doi:10.1101/gr.205468.116)
Supplement: Supplemental Material [file supp_gr.205468.116_Supplemental_Data_1.zip › FIGURE3_DSS_DMR_analysis_sept25.html]

Brachypodium Replicate Methylome DSS DMR calling


# Brachypodium Replicate Methylome DSS DMR calling

#### *SRE*

#### *29 September 2015*

```
library(ggplot2)
library(xts)
library(grid)
source('ggcorr.R')
```

# Correlation data across proportion methylation levels of all replicate and experiment samples

```
cg=read.delim('CG.proportion.met.correlation_including_first_samples.txt',head=T)
chg=read.delim('CHG.proportion.met.correlation_including_first_samples.txt',head=T)
chh=read.delim('CHH.proportion.met.correlation_including_first_samples.txt',head=T)
ggcorr(data=NULL,cor_matrix=as.matrix(cg),hjust=0.9) + scale_fill_continuous(low = "grey", high = "black",limits=c(0.8,1))
```

```
## Warning: Non Lab interpolation is deprecated
```

```
## Scale for 'fill' is already present. Adding another scale for 'fill', which will replace the existing scale.
```

```
ggcorr(data=NULL,cor_matrix=as.matrix(chg),hjust=0.9) + scale_fill_continuous(low = "grey", high = "black",limits=c(0.8,1))
```

```
## Warning: Non Lab interpolation is deprecated
```

```
## Scale for 'fill' is already present. Adding another scale for 'fill', which will replace the existing scale.
```

```
ggcorr(data=NULL,cor_matrix=as.matrix(chh),hjust=0.9) + scale_fill_continuous(low = "grey", high = "black",limits=c(0,1))
```

```
## Warning: Non Lab interpolation is deprecated
```

```
## Scale for 'fill' is already present. Adding another scale for 'fill', which will replace the existing scale.
```

Note scale change for chh. Replicate data matches well with each other as well as the matched experimental sample. Very high overall correlations for CG and CHG. CHH is much lower (0.3-0.5).

# DMRs

Akanksha has provided full sets of DMRs called via the DSS package and method. There are two sets of output files for calling DMRs across the Brachypodium genome as a set of DMRs called pairwise between our three replicate samples (Bd21, Bd1-1, and Bd3-1) for all three sequence contexts (CG, CHG, and CHH):

1. DSS was set to smooth over missing data (impute places) and no data exclusion was conducted. This is the ‘default’ DSS method. It also is the method that will call the largest number of DMRs and the least conservative.
2. DSS did not smooth over missing data. No data exclusion was conducted. This method should be somewhat more strict than method one while still not requiring coverage thresholds for either depth or replicate number.

The `txt` files provided are now read into R:

```
smooth.1vs3.cg=read.delim('DMRs_all_smooth/dmr.sm_Bd1vs3_CG.txt',head=T)
smooth.1vs3.cg$contrast="Bd1-1vsBd3-1"
smooth.1vs3.cg$context="CG"
smooth.1vs3.chg=read.delim('DMRs_all_smooth/dmr.sm_Bd1vs3_CHG.txt',head=T)
smooth.1vs3.chg$contrast="Bd1-1vsBd3-1"
smooth.1vs3.chg$context="CHG"
smooth.1vs3.chh=read.delim('DMRs_all_smooth/dmr.sm_Bd1vs3_CHH.txt',head=T)
smooth.1vs3.chh$contrast="Bd1-1vsBd3-1"
smooth.1vs3.chh$context="CHH"

smooth.21vs1.cg=read.delim('DMRs_all_smooth/dmr.sm_Bd21vs1_CG.txt',head=T)
smooth.21vs1.cg$contrast="Bd21vsBd1-1"
smooth.21vs1.cg$context="CG"
smooth.21vs1.chg=read.delim('DMRs_all_smooth/dmr.sm_Bd21vs1_CHG.txt',head=T)
smooth.21vs1.chg$contrast="Bd21vsBd1-1"
smooth.21vs1.chg$context="CHG"
smooth.21vs1.chh=read.delim('DMRs_all_smooth/dmr.sm_Bd21vs1_CHH.txt',head=T)
smooth.21vs1.chh$contrast="Bd21vsBd1-1"
smooth.21vs1.chh$context="CHH"

smooth.21vs3.cg=read.delim('DMRs_all_smooth/dmr.sm_Bd21vs3_CG.txt',head=T)
smooth.21vs3.cg$contrast="Bd21vsBd3-1"
smooth.21vs3.cg$context="CG"
smooth.21vs3.chg=read.delim('DMRs_all_smooth/dmr.sm_Bd21vs3_CHG.txt',head=T)
smooth.21vs3.chg$contrast="Bd21vsBd3-1"
smooth.21vs3.chg$context="CHG"
smooth.21vs3.chh=read.delim('DMRs_all_smooth/dmr.sm_Bd21vs3_CHH.txt',head=T)
smooth.21vs3.chh$contrast="Bd21vsBd3-1"
smooth.21vs3.chh$context="CHH"

smooth.all=rbind(smooth.1vs3.cg,smooth.1vs3.chg,smooth.1vs3.chh,smooth.21vs1.cg,smooth.21vs1.chg,smooth.21vs1.chh,smooth.21vs3.cg,smooth.21vs3.chg,smooth.21vs3.chh)

#this is to remove the additional header lines that were errors in the creation of the file.
smooth.all=smooth.all[grep("^chr",smooth.all$chr,invert=T),]

#non-smoothed DMRs
unsmooth.1vs3.cg=read.delim('DMRs_all_unsmooth/dmr_Bd1vs3_CG.txt',head=T)
unsmooth.1vs3.cg$contrast="Bd1-1vsBd3-1"
unsmooth.1vs3.cg$context="CG"
unsmooth.1vs3.chg=read.delim('DMRs_all_unsmooth/dmr_Bd1vs3_CHG.txt',head=T)
unsmooth.1vs3.chg$contrast="Bd1-1vsBd3-1"
unsmooth.1vs3.chg$context="CHG"
unsmooth.1vs3.chh=read.delim('DMRs_all_unsmooth/dmr_Bd1vs3_CHH.txt',head=T)
unsmooth.1vs3.chh$contrast="Bd1-1vsBd3-1"
unsmooth.1vs3.chh$context="CHH"

unsmooth.21vs1.cg=read.delim('DMRs_all_unsmooth/dmr_Bd21vs1_CG.txt',head=T)
unsmooth.21vs1.cg$contrast="Bd21vsBd1-1"
unsmooth.21vs1.cg$context="CG"
unsmooth.21vs1.chg=read.delim('DMRs_all_unsmooth/dmr_Bd21vs1_CHG.txt',head=T)
unsmooth.21vs1.chg$contrast="Bd21vsBd1-1"
unsmooth.21vs1.chg$context="CHG"
unsmooth.21vs1.chh=read.delim('DMRs_all_unsmooth/dmr_Bd21vs1_CHH.txt',head=T)
unsmooth.21vs1.chh$contrast="Bd21vsBd1-1"
unsmooth.21vs1.chh$context="CHH"

unsmooth.21vs3.cg=read.delim('DMRs_all_unsmooth/dmr_Bd21vs3_CG.txt',head=T)
unsmooth.21vs3.cg$contrast="Bd21vsBd3-1"
unsmooth.21vs3.cg$context="CG"
unsmooth.21vs3.chg=read.delim('DMRs_all_unsmooth/dmr_Bd21vs3_CHG.txt',head=T)
unsmooth.21vs3.chg$contrast="Bd21vsBd3-1"
unsmooth.21vs3.chg$context="CHG"
unsmooth.21vs3.chh=read.delim('DMRs_all_unsmooth/dmr_Bd21vs3_CHH.txt',head=T)
unsmooth.21vs3.chh$contrast="Bd21vsBd3-1"
unsmooth.21vs3.chh$context="CHH"

unsmooth.all=rbind(unsmooth.1vs3.cg,unsmooth.1vs3.chg,unsmooth.1vs3.chh,unsmooth.21vs1.cg,unsmooth.21vs1.chg,unsmooth.21vs1.chh,unsmooth.21vs3.cg,unsmooth.21vs3.chg,unsmooth.21vs3.chh)

#this is to remove the additional header lines that were errors in the creation of the file.
unsmooth.all=unsmooth.all[grep("^chr",unsmooth.all$chr,invert=T),]

a=table(smooth.all$contrast,smooth.all$context)
b=table(unsmooth.all$contrast,unsmooth.all$context)

ggplot(as.data.frame(a),aes(Var2,Freq))+geom_bar(aes(fill=Var1),stat="identity",position="dodge") + theme_sre + xlab("Contrast") + ylab("Number of DMRs") + ggtitle("Smooth DMRs as called by DSS by contrast and context") + labs(fill="DMR Contrast")
```

```
table(smooth.all$contrast,smooth.all$context)
```

```
##               
##                   CG   CHG   CHH
##   Bd1-1vsBd3-1 49998 22483 19770
##   Bd21vsBd1-1  50349 22127 17803
##   Bd21vsBd3-1  35226 12484  9354
```

```
ggplot(as.data.frame(b),aes(Var2,Freq))+geom_bar(aes(fill=Var1),stat="identity",position="dodge") + theme_sre + xlab("Contrast") + ylab("Number of DMRs") + ggtitle("Unsmooth DMRs as called by DSS by contrast and context") + labs(fill="DMR Contrast")
```

```
table(unsmooth.all$contrast,unsmooth.all$context)
```

```
##               
##                   CG   CHG   CHH
##   Bd1-1vsBd3-1 21497 12347   288
##   Bd21vsBd1-1  23005 13377   347
##   Bd21vsBd3-1  17441  7703   166
```

Many more DMRs are identified in the smoothed data compared to the unsmoothed with a simstrantial decrease in CHH DMR number for unsmoothed calls. Bd21 vs Bd3-1 shows the fewest DMRs across contexts for both smoothed and unsmoothed data.

```
ggplot(smooth.all) + geom_density(aes(as.numeric(length),fill=factor(contrast)),alpha=0.5,size=1) + theme_sre + xlab("Smooth DSS DMR size") + scale_x_continuous(limits=c(0,4000)) +facet_grid(. ~ context)
```

Smooth DMRs vary greatly in size with the majority around ~1600bp in size. This is suprisingly consistent for all three sequence contexts

```
ggplot(unsmooth.all) + geom_density(aes(as.numeric(length),fill=factor(contrast)),alpha=0.5,size=1) + theme_sre + xlab("Unsmooth DSS DMR size") + scale_x_continuous(limits=c(0,4000)) +facet_grid(. ~ context)
```

Unsmoothed DMRs have a very different distribution with DMRs being either sub 500bp or greater than 500bp. CHH DMRs are more often 800-1000bp in size.

```
ggplot(smooth.all) + geom_density(aes(as.numeric(nCG),fill=factor(contrast)),alpha=0.5,size=1) + theme_sre + xlab("Smooth DSS DMR site count") + facet_grid(. ~ context)
```

Looking at the number of cytosines in each DMR context, appears to be some variation with cytosine counts upwards of 500 for CG and CHG DMRs (with many much less). CHH DMRs have a few with sites over 800.

```
ggplot(unsmooth.all) + geom_density(aes(as.numeric(nCG),fill=factor(contrast)),alpha=0.5,size=1) + theme_sre + xlab("Unsmooth DSS DMR site count") + facet_grid(. ~ context) + ggtitle("Unsmooth DSS DMRs - DMR site count")
```

The unsmoothed dataset again looks very different. DMRs have less than 300 cytosines in them with DMRs largely falling into two classes (those with ~50 or fewer sites and those ~200 or so). All three contrasts have very similar distributions. CHH shows a bit more variation likely due to the much smaller sample size.

```
ggplot(smooth.all) + geom_density(aes(as.numeric(as.character(diff.Methy)),fill=factor(contrast)),alpha=0.5,size=1) + theme_sre + xlab("Smooth DSS DMR methylation difference") + facet_grid(. ~ context) + ggtitle("Smooth DSS DMRs - Difference in methylation value")
```

When looking at the difference in group methylation values for DMRs. We see that many differences are being called with less than a 50% change in methylation. This is very different to the unsmoothed data (below) in which changes are often at 50% or more in any called DMR. Smoothing likely imputes enough data to increase power and call DMRs at a lower threshold and still meet the significance requirements.

Also, in smooth DMRs, Bd21 appears to have more higher methylation states compared to Bd3-1 and Bd1-1. Bd3-1 appears to have higher states than Bd3-1 (red distribution greater <0).

```
ggplot(unsmooth.all) + geom_density(aes(as.numeric(as.character(diff.Methy)),fill=factor(contrast)),alpha=0.5,size=1) + theme_sre + xlab("Unsmooth DSS DMR methylation difference") + facet_grid(. ~ context) + ggtitle("Unsmooth DSS DMRs - Difference in methylation value")
```

```
ggplot(smooth.all) + geom_density(aes(as.numeric(as.character(areaStat)),fill=factor(contrast)),alpha=0.5,size=1) + theme_sre + xlab("Smooth DSS areaStat") + facet_grid(. ~ context) + xlim(-5000,5000) + ggtitle("Smooth DSS DMRs - areaStatistic")
```

The areaStat describes the sum of test statistics for all sites within the region (DMR). Therefore, the direction is tied to methylation difference direction with more areaStat values being <0 for Bd21 contrasts, as Bd21 has less methylation at DMRs compared to either of the other inbreds.

```
ggplot(unsmooth.all) + geom_density(aes(as.numeric(as.character(areaStat)),fill=factor(contrast)),alpha=0.5,size=1) + theme_sre + xlab("Unsmooth DSS areaStat") + facet_grid(. ~ context) + xlim(-5000,5000) + ggtitle("Unsmooth DSS DMRs - areaStatistic")
```

```
write.table(smooth.all,'DSS_smooth.all.bed',sep='\t',row.names=F,quote=F,col.names=F)
write.table(unsmooth.all,'DSS_unsmooth.all.bed',sep='\t',row.names=F,quote=F,col.names=F)
```

For the manuscript. I am going to focus on the contrasts that include Bd21. This allows for a more direct comparison to the 100bp tile DMR set. A key question and goal of the DSS approach is that replicates may allow for more nuanced DMRs to be called (i.e. quantitative variation). The 100bp tile data has a set of fixed thresholds of a difference in methylation proportion between the two samples. To compare, the absolute value of mean methylation difference from DSS groups (replicates) was plotted for all Bd21-included DMRs by context:

```
unsmooth.set=subset(unsmooth.all,unsmooth.all$contrast!='Bd1-1vsBd3-1')
ggplot(unsmooth.set) + geom_density(aes(abs(as.numeric(as.character(diff.Methy))),fill=factor(context)),alpha=0.5,size=1) + theme_sre + xlab("Unsmooth DSS DMR methylation difference") + ggtitle("Unsmooth DSS DMRs vs Bd21only - Absolute Difference in methylation value") + geom_vline(xintercept=0.7,colour="red") + geom_vline(xintercept=0.5,colour="blue")
```

From this, it is clear that the majority of CG DMRs are showing a similar methylation difference at, or beyond, the 100bp method threshold of 0.7. CHG is a bit more loose with some DMRs being called at < 50% difference.

```
unsmooth.set.CG=subset(unsmooth.set,unsmooth.set$context=='CG')
unsmooth.set.CHG=subset(unsmooth.set,unsmooth.set$context=='CHG')
unsmooth.set.CHH=subset(unsmooth.set,unsmooth.set$context=='CHH')
#number of CG DMRs that are under 100bp threshold
dim(subset(unsmooth.set.CG,abs(as.numeric(as.character(unsmooth.set.CG$diff.Methy))) < 0.7))
```

```
## [1] 13412    11
```

```
#number of CHG DMRs that are under 100bp threshold
dim(subset(unsmooth.set.CHG,abs(as.numeric(as.character(unsmooth.set.CHG$diff.Methy))) < 0.5))
```

```
## [1] 5640   11
```

```
#number of CG DMRs that are under 100bp threshold
dim(subset(unsmooth.set.CHH,(as.numeric(as.character(unsmooth.set.CHH$meanMethy1)) < 0.05 & as.numeric(as.character(unsmooth.set.CHH$meanMethy2)) > 0.20) | (as.numeric(as.character(unsmooth.set.CHH$meanMethy2)) < 0.05 & as.numeric(as.character(unsmooth.set.CHH$meanMethy1)) > 0.20)))
```

```
## [1] 116  11
```

```
library(VennDiagram)
```

```
## Warning: package 'VennDiagram' was built under R version 3.1.3
```

```
## Loading required package: futile.logger
```

```
## Warning: package 'futile.logger' was built under R version 3.1.3
```

```
draw.pairwise.venn(40446,15138,14265,fill=c('yellow','blue'))
```

```
## (polygon[GRID.polygon.1460], polygon[GRID.polygon.1461], polygon[GRID.polygon.1462], polygon[GRID.polygon.1463], text[GRID.text.1464], text[GRID.text.1465], lines[GRID.lines.1466], text[GRID.text.1467], text[GRID.text.1468], text[GRID.text.1469])
```

CG DMRS are largely overlapping. Blue is 100bp tile set, yellow is DSS unsmoothed set

```
draw.pairwise.venn(21080,12964,8778,fill=c('yellow','blue'))
```

```
## (polygon[GRID.polygon.1470], polygon[GRID.polygon.1471], polygon[GRID.polygon.1472], polygon[GRID.polygon.1473], text[GRID.text.1474], text[GRID.text.1475], text[GRID.text.1476], text[GRID.text.1477], text[GRID.text.1478])
```

CHG DMRs less so

```
draw.pairwise.venn(513,1441,54,fill=c('yellow','blue'))
```

```
## (polygon[GRID.polygon.1479], polygon[GRID.polygon.1480], polygon[GRID.polygon.1481], polygon[GRID.polygon.1482], text[GRID.text.1483], text[GRID.text.1484], text[GRID.text.1485], lines[GRID.lines.1486], text[GRID.text.1487], text[GRID.text.1488])
```

CHH shows basically no overlap at all. Highlighting apparent variation.

## Looking at ‘conservative’ DSS DMR calling

This requires that there is coverage across all samples in the comparison (i.e. no NA values for a cytosine)

```
smooth.1vs3.cg=read.delim('conservative_method_results/dmr.sm_Bd1vs3_CG.txt',head=T)
smooth.1vs3.cg$contrast="Bd1-1vsBd3-1"
smooth.1vs3.cg$context="CG"
smooth.1vs3.chg=read.delim('conservative_method_results/dmr.sm_Bd1vs3_CHG.txt',head=T)
smooth.1vs3.chg$contrast="Bd1-1vsBd3-1"
smooth.1vs3.chg$context="CHG"
smooth.1vs3.chh=read.delim('conservative_method_results/dmr.sm_Bd1vs3_CHH.txt',head=T)
smooth.1vs3.chh$contrast="Bd1-1vsBd3-1"
smooth.1vs3.chh$context="CHH"

smooth.21vs1.cg=read.delim('conservative_method_results/dmr.sm_Bd21vs1_CG.txt',head=T)
smooth.21vs1.cg$contrast="Bd21vsBd1-1"
smooth.21vs1.cg$context="CG"
smooth.21vs1.chg=read.delim('conservative_method_results/dmr.sm_Bd21vs1_CHG.txt',head=T)
smooth.21vs1.chg$contrast="Bd21vsBd1-1"
smooth.21vs1.chg$context="CHG"
smooth.21vs1.chh=read.delim('conservative_method_results/dmr.sm_Bd21vs1_CHH.txt',head=T)
smooth.21vs1.chh$contrast="Bd21vsBd1-1"
smooth.21vs1.chh$context="CHH"

smooth.21vs3.cg=read.delim('conservative_method_results/dmr.sm_Bd21vs3_CG.txt',head=T)
smooth.21vs3.cg$contrast="Bd21vsBd3-1"
smooth.21vs3.cg$context="CG"
smooth.21vs3.chg=read.delim('conservative_method_results/dmr.sm_Bd21vs3_CHG.txt',head=T)
smooth.21vs3.chg$contrast="Bd21vsBd3-1"
smooth.21vs3.chg$context="CHG"
smooth.21vs3.chh=read.delim('conservative_method_results/dmr.sm_Bd21vs3_CHH.txt',head=T)
smooth.21vs3.chh$contrast="Bd21vsBd3-1"
smooth.21vs3.chh$context="CHH"

smooth.all=rbind(smooth.1vs3.cg,smooth.1vs3.chg,smooth.1vs3.chh,smooth.21vs1.cg,smooth.21vs1.chg,smooth.21vs1.chh,smooth.21vs3.cg,smooth.21vs3.chg,smooth.21vs3.chh)

#this is to remove the additional header lines that were errors in the creation of the file.
smooth.all=smooth.all[grep("^chr",smooth.all$chr,invert=T),]

#non-smoothed DMRs
unsmooth.1vs3.cg=read.delim('conservative_method_results/dmr_Bd1vs3_CG.txt',head=T)
unsmooth.1vs3.cg$contrast="Bd1-1vsBd3-1"
unsmooth.1vs3.cg$context="CG"
unsmooth.1vs3.chg=read.delim('conservative_method_results/dmr_Bd1vs3_CHG.txt',head=T)
unsmooth.1vs3.chg$contrast="Bd1-1vsBd3-1"
unsmooth.1vs3.chg$context="CHG"
unsmooth.1vs3.chh=read.delim('conservative_method_results/dmr_Bd1vs3_CHH.txt',head=T)
unsmooth.1vs3.chh$contrast="Bd1-1vsBd3-1"
unsmooth.1vs3.chh$context="CHH"

unsmooth.21vs1.cg=read.delim('conservative_method_results/dmr_Bd21vs1_CG.txt',head=T)
unsmooth.21vs1.cg$contrast="Bd21vsBd1-1"
unsmooth.21vs1.cg$context="CG"
unsmooth.21vs1.chg=read.delim('conservative_method_results/dmr_Bd21vs1_CHG.txt',head=T)
unsmooth.21vs1.chg$contrast="Bd21vsBd1-1"
unsmooth.21vs1.chg$context="CHG"
unsmooth.21vs1.chh=read.delim('conservative_method_results/dmr_Bd21vs1_CHH.txt',head=T)
unsmooth.21vs1.chh$contrast="Bd21vsBd1-1"
unsmooth.21vs1.chh$context="CHH"

unsmooth.21vs3.cg=read.delim('conservative_method_results/dmr_Bd21vs3_CG.txt',head=T)
unsmooth.21vs3.cg$contrast="Bd21vsBd3-1"
unsmooth.21vs3.cg$context="CG"
unsmooth.21vs3.chg=read.delim('conservative_method_results/dmr_Bd21vs3_CHG.txt',head=T)
unsmooth.21vs3.chg$contrast="Bd21vsBd3-1"
unsmooth.21vs3.chg$context="CHG"
unsmooth.21vs3.chh=read.delim('conservative_method_results/dmr_Bd21vs3_CHH.txt',head=T)
unsmooth.21vs3.chh$contrast="Bd21vsBd3-1"
unsmooth.21vs3.chh$context="CHH"

unsmooth.all=rbind(unsmooth.1vs3.cg,unsmooth.1vs3.chg,unsmooth.1vs3.chh,unsmooth.21vs1.cg,unsmooth.21vs1.chg,unsmooth.21vs1.chh,unsmooth.21vs3.cg,unsmooth.21vs3.chg,unsmooth.21vs3.chh)

#this is to remove the additional header lines that were errors in the creation of the file.
unsmooth.all=unsmooth.all[grep("^chr",unsmooth.all$chr,invert=T),]

a=table(smooth.all$contrast,smooth.all$context)
b=table(unsmooth.all$contrast,unsmooth.all$context)

ggplot(as.data.frame(a),aes(Var2,Freq))+geom_bar(aes(fill=Var1),stat="identity",position="dodge") + theme_sre + xlab("Contrast") + ylab("Number of DMRs") + ggtitle("Smooth DMRs as called by DSS by contrast and context") + labs(fill="DMR Contrast")
```

```
table(smooth.all$contrast,smooth.all$context)
```

```
##               
##                   CG   CHG   CHH
##   Bd1-1vsBd3-1 18941 11645  2679
##   Bd21vsBd1-1  29853 16227  7839
##   Bd21vsBd3-1  22071 10108  4317
```

```
ggplot(as.data.frame(b),aes(Var2,Freq))+geom_bar(aes(fill=Var1),stat="identity",position="dodge") + theme_sre + xlab("Contrast") + ylab("Number of DMRs") + ggtitle("Unsmooth DMRs as called by DSS by contrast and context") + labs(fill="DMR Contrast")
```

```
table(unsmooth.all$contrast,unsmooth.all$context)
```

```
##               
##                   CG   CHG   CHH
##   Bd1-1vsBd3-1 12722  9743   198
##   Bd21vsBd1-1  11437  9129   209
##   Bd21vsBd3-1   8401  5069    98
```

```
ggplot(smooth.all) + geom_density(aes(as.numeric(length),fill=factor(contrast)),alpha=0.5,size=1) + theme_sre + xlab("Smooth DSS DMR size") + scale_x_continuous(limits=c(0,4000)) +facet_grid(. ~ context)
```

```
ggplot(unsmooth.all) + geom_density(aes(as.numeric(length),fill=factor(contrast)),alpha=0.5,size=1) + theme_sre + xlab("Unsmooth DSS DMR size") + scale_x_continuous(limits=c(0,4000)) +facet_grid(. ~ context)
```

```
ggplot(smooth.all) + geom_density(aes(as.numeric(nCG),fill=factor(contrast)),alpha=0.5,size=1) + theme_sre + xlab("Smooth DSS DMR site count") + facet_grid(. ~ context)
```

```
ggplot(unsmooth.all) + geom_density(aes(as.numeric(nCG),fill=factor(contrast)),alpha=0.5,size=1) + theme_sre + xlab("Unsmooth DSS DMR site count") + facet_grid(. ~ context) + ggtitle("Unsmooth DSS DMRs - DMR site count")
```

```
ggplot(smooth.all) + geom_density(aes(as.numeric(as.character(diff.Methy)),fill=factor(contrast)),alpha=0.5,size=1) + theme_sre + xlab("Smooth DSS DMR methylation difference") + facet_grid(. ~ context) + ggtitle("Smooth DSS DMRs - Difference in methylation value")
```

```
ggplot(unsmooth.all) + geom_density(aes(as.numeric(as.character(diff.Methy)),fill=factor(contrast)),alpha=0.5,size=1) + theme_sre + xlab("Unsmooth DSS DMR methylation difference") + facet_grid(. ~ context) + ggtitle("Unsmooth DSS DMRs - Difference in methylation value")
```

```
ggplot(smooth.all) + geom_density(aes(as.numeric(as.character(areaStat)),fill=factor(contrast)),alpha=0.5,size=1) + theme_sre + xlab("Smooth DSS areaStat") + facet_grid(. ~ context) + xlim(-5000,5000) + ggtitle("Smooth DSS DMRs - areaStatistic")
```

```
ggplot(unsmooth.all) + geom_density(aes(as.numeric(as.character(areaStat)),fill=factor(contrast)),alpha=0.5,size=1) + theme_sre + xlab("Unsmooth DSS areaStat") + facet_grid(. ~ context) + xlim(-5000,5000) + ggtitle("Unsmooth DSS DMRs - areaStatistic")
```

```
write.table(smooth.all,'conservative_method_results/DSS_smooth.all.bed',sep='\t',row.names=F,quote=F,col.names=F)
write.table(unsmooth.all,'conservative_method_results/DSS_unsmooth.all.bed',sep='\t',row.names=F,quote=F,col.names=F)
```

```
unsmooth.set=subset(unsmooth.all,unsmooth.all$contrast!='Bd1-1vsBd3-1')
ggplot(unsmooth.set) + geom_density(aes(abs(as.numeric(as.character(diff.Methy))),fill=factor(context)),alpha=0.5,size=1) + theme_sre + xlab("Unsmooth DSS DMR methylation difference") + ggtitle("Unsmooth DSS DMRs vs Bd21only - Absolute Difference in methylation value") + geom_vline(xintercept=0.7,colour="red") + geom_vline(xintercept=0.5,colour="blue")
```

```
unsmooth.set.CG=subset(unsmooth.set,unsmooth.set$context=='CG')
unsmooth.set.CHG=subset(unsmooth.set,unsmooth.set$context=='CHG')
unsmooth.set.CHH=subset(unsmooth.set,unsmooth.set$context=='CHH')
#number of CG DMRs that are under 100bp threshold
dim(subset(unsmooth.set.CG,abs(as.numeric(as.character(unsmooth.set.CG$diff.Methy))) < 0.7))
```

```
## [1] 6323   11
```

```
#number of CHG DMRs that are under 100bp threshold
dim(subset(unsmooth.set.CHG,abs(as.numeric(as.character(unsmooth.set.CHG$diff.Methy))) < 0.5))
```

```
## [1] 4205   11
```

```
#number of CG DMRs that are under 100bp threshold
dim(subset(unsmooth.set.CHH,(as.numeric(as.character(unsmooth.set.CHH$meanMethy1)) < 0.05 & as.numeric(as.character(unsmooth.set.CHH$meanMethy2)) > 0.20) | (as.numeric(as.character(unsmooth.set.CHH$meanMethy2)) < 0.05 & as.numeric(as.character(unsmooth.set.CHH$meanMethy1)) > 0.20)))
```

```
## [1] 127  11
```

```
library(VennDiagram)
draw.pairwise.venn(19838,15138,11769,fill=c('yellow','blue'))
```

```
## (polygon[GRID.polygon.2803], polygon[GRID.polygon.2804], polygon[GRID.polygon.2805], polygon[GRID.polygon.2806], text[GRID.text.2807], text[GRID.text.2808], text[GRID.text.2809], text[GRID.text.2810], text[GRID.text.2811])
```

CG DMRs: Blue is 100bp tile set, yellow is DSS unsmoothed set

```
draw.pairwise.venn(14198,12964,7684,fill=c('yellow','blue'))
```

```
## (polygon[GRID.polygon.2812], polygon[GRID.polygon.2813], polygon[GRID.polygon.2814], polygon[GRID.polygon.2815], text[GRID.text.2816], text[GRID.text.2817], text[GRID.text.2818], text[GRID.text.2819], text[GRID.text.2820])
```

CHG DMRs: Blue is 100bp tile set, yellow is DSS unsmoothed set

```
draw.pairwise.venn(307,1441,44,fill=c('yellow','blue'))
```

```
## (polygon[GRID.polygon.2821], polygon[GRID.polygon.2822], polygon[GRID.polygon.2823], polygon[GRID.polygon.2824], text[GRID.text.2825], text[GRID.text.2826], text[GRID.text.2827], lines[GRID.lines.2828], text[GRID.text.2829], text[GRID.text.2830])
```

CHH DMRs: Blue is 100bp tile set, yellow is DSS unsmoothed set
