# Supplementary material for: DNA methylation profiles of diverse Brachypodium distachyon align with underlying genetic diversity
Source: Genome Res. 2016 Nov;26(11):1520–31. doi: 10.1101/gr.205468.116 (PMC5088594; doi:10.1101/gr.205468.116)
Supplement: Supplemental Material [file supp_gr.205468.116_Supplemental_Data_1.zip › FIGURE5_TEPID_to_DMRs_v5.html]

locaTE results to DMR v5


# locaTE results to DMR v5

#### *SRE*

#### *7 August 2015*

0 - Get number of insertions per sample

```
in.list=dir(pattern='^insertions_*')
geno.list=matrix(unlist(strsplit(as.character(in.list),'_')),ncol=2,byrow=T)[,2]
insertions=NULL

for(i in 1:length(in.list)){
  file=read.delim(in.list[i],head=F)
  samplename=matrix(rep(geno.list[i],nrow(file)),ncol=1)
  file=cbind(file,samplename)
  insertions=rbind(insertions,file)
}

counts=table(insertions$samplename)
counts.in=counts
barplot(counts,main='number of insertions per genotype',col=1,las=2)
```

```
a=table(insertions$V1, insertions$samplename)
barplot(a[1:5,],beside=T,las=2,main='Number of insertions per chromosome')
```

Look at insertions vs genes:

```
bedtools closest -D "ref" -a insertions_Bd1-1.bed -b Bdis.gene.bed > Bd1-1.genes.to.insertions.txt
bedtools closest -D "ref" -a insertions_Bd21-3.bed -b Bdis.gene.bed > Bd21-3.genes.to.insertions.txt
bedtools closest -D "ref" -a insertions_Bd3-1.bed -b Bdis.gene.bed > Bd3-1.genes.to.insertions.txt
bedtools closest -D "ref" -a insertions_Bd30-1.bed -b Bdis.gene.bed > Bd30-1.genes.to.insertions.txt
bedtools closest -D "ref" -a insertions_BdTR12c.bed -b Bdis.gene.bed > BdTR12c.genes.to.insertions.txt
bedtools closest -D "ref" -a insertions_Koz-3.bed -b Bdis.gene.bed > Koz-3.genes.to.insertions.txt
```

```
count.close=counts

in.genes.list=dir(pattern='*genes.to.insertions.txt')
insertions.to.genes=NULL
for(i in 1:length(in.genes.list)){
  temp=read.delim(in.genes.list[i],head=F)
  temp=cbind(temp,rep(in.genes.list[i],nrow(temp)))
  insertions.to.genes=rbind(insertions.to.genes,temp)
  }
colnames(insertions.to.genes)[17]='V17'
insertions.to.genes=subset(insertions.to.genes,insertions.to.genes$V16 != -1)
insertions.sub=subset(insertions.to.genes, insertions.to.genes$V16 < 500 & insertions.to.genes$V16 > -500)

close.dmrs=table(insertions.sub$V17)
count.close=rbind(count.close,close.dmrs[1:6])

rownames(count.close)=c('total insertions','insertions with gene within 500bp')
barplot(count.close,main='Number of TE insertions with gene within 500bp',las=2,beside=T,legend=rownames(count.close))
```

```
#proportion of insertions with gene within 500bp:
count.close[2,] / count.close[1,]
```

```
##   Bd1-1.bed  Bd21-3.bed   Bd3-1.bed  Bd30-1.bed BdTR12c.bed   Koz-3.bed 
##   0.2817680   0.3166667   0.2173913   0.2656250   0.2254902   0.3012048
```

# Insertions to CG DMRs

1 - split DMRs to individual sample sets

```
cg.dmrs=read.delim('CGDMRS.all.txt',head=T)
cg.dmrs.bed=cg.dmrs[,c(4,5,6,2,7,1,8,3)]

dmr.type=c('Bd1-1.CG','Bd21-3.CG','Bd3-1.CG','Bd30-1.CG','BdTR12c.CG','Koz-3.CG')
for(i in 1:length(dmr.type)){
  temp=subset(cg.dmrs.bed,cg.dmrs.bed[,8]==dmr.type[i])
  write.table(temp,paste(dmr.type[i],'DMRS.bed',sep='.'),sep='\t',row.names=F,quote=F,col.names=F)

}
```

2 - Map to locaTE insertion files

```
bedtools closest -D "ref" -a insertions_Bd1-1.bed -b Bd1-1.CG.DMRS.bed > Bd1-1.CG.DMRs.to.insertions.txt
bedtools closest -D "ref" -a insertions_Bd21-3.bed -b Bd21-3.CG.DMRS.bed > Bd21-3.CG.DMRs.to.insertions.txt
bedtools closest -D "ref" -a insertions_Bd3-1.bed -b Bd3-1.CG.DMRS.bed > Bd3-1.CG.DMRs.to.insertions.txt
bedtools closest -D "ref" -a insertions_Bd30-1.bed -b Bd30-1.CG.DMRS.bed > Bd30-1.CG.DMRs.to.insertions.txt
bedtools closest -D "ref" -a insertions_BdTR12c.bed -b BdTR12c.CG.DMRS.bed > BdTR12c.CG.DMRs.to.insertions.txt
bedtools closest -D "ref" -a insertions_Koz-3.bed -b Koz-3.CG.DMRS.bed > Koz-3.CG.DMRs.to.insertions.txt
```

3 - Compare total number of insertions to those insertions with DMRs within 500bp

```
count.close=counts

in.dmr.list=dir(pattern='*CG.DMRs.to.insertions.txt')
insertions.to.dmrs=NULL
for(i in 1:length(in.dmr.list)){
  temp=read.delim(in.dmr.list[i],head=F)
  insertions.to.dmrs=rbind(insertions.to.dmrs,temp)
  }

#remove the shit scaffold things with no dmrs to target
insertions.to.dmrs=subset(insertions.to.dmrs,insertions.to.dmrs$V17 != -1)
insertions.sub=subset(insertions.to.dmrs, insertions.to.dmrs$V17 < 500 & insertions.to.dmrs$V17 > -500)

close.dmrs=table(insertions.sub$V16)
count.close=rbind(count.close,close.dmrs)

rownames(count.close)=c('total insertions','insertions with CG DMRs within 500bp')
barplot(count.close,main='Number of TE insertions with CG DMRs within 500bp',las=2,beside=T,legend=rownames(count.close))
```

```
#proportion of insertions with DMRs within 500bp:
count.close[2,] / count.close[1,]
```

```
##   Bd1-1.bed  Bd21-3.bed   Bd3-1.bed  Bd30-1.bed BdTR12c.bed   Koz-3.bed 
##  0.10497238  0.06666667  0.13043478  0.15625000  0.10784314  0.09638554
```

```
dmr.type=table(insertions.sub$V16, insertions.sub$V15)
dmr.type=dmr.type

rownames(dmr.type)=c('Bd1-1','Bd21-3','Bd3-1','Bd30-1','BdTR12c','Koz-3')
barplot(t(dmr.type),las=2,col=c(3,4),legend=rownames(t(dmr.type)),beside=T,main='Type of CG DMR compared to reference Bd21')
```

```
cg.count.close=count.close
```

# Insertions to CHG DMRs

1 - split DMRs to individual sample sets

```
CHG.dmrs=read.delim('CHGDMRS.all.txt',head=T)
CHG.dmrs.bed=CHG.dmrs[,c(4,5,6,2,7,1,8,3)]

dmr.type=c('Bd1-1.CHG','Bd21-3.CHG','Bd3-1.CHG','Bd30-1.CHG','BdTR12c.CHG','Koz-3.CHG')
for(i in 1:length(dmr.type)){
  temp=subset(CHG.dmrs.bed,CHG.dmrs.bed[,8]==dmr.type[i])
  write.table(temp,paste(dmr.type[i],'DMRS.bed',sep='.'),sep='\t',row.names=F,quote=F,col.names=F)

}
```

2 - Map to locaTE insertion files

```
bedtools closest -D "ref" -a insertions_Bd1-1.bed -b Bd1-1.CHG.DMRS.bed > Bd1-1.CHG.DMRs.to.insertions.txt
bedtools closest -D "ref" -a insertions_Bd21-3.bed -b Bd21-3.CHG.DMRS.bed > Bd21-3.CHG.DMRs.to.insertions.txt
bedtools closest -D "ref" -a insertions_Bd3-1.bed -b Bd3-1.CHG.DMRS.bed > Bd3-1.CHG.DMRs.to.insertions.txt
bedtools closest -D "ref" -a insertions_Bd30-1.bed -b Bd30-1.CHG.DMRS.bed > Bd30-1.CHG.DMRs.to.insertions.txt
bedtools closest -D "ref" -a insertions_BdTR12c.bed -b BdTR12c.CHG.DMRS.bed > BdTR12c.CHG.DMRs.to.insertions.txt
bedtools closest -D "ref" -a insertions_Koz-3.bed -b Koz-3.CHG.DMRS.bed > Koz-3.CHG.DMRs.to.insertions.txt
```

3 - Compare total number of insertions to those insertions with DMRs within 500bp

```
count.close=counts

in.dmr.list=dir(pattern='*CHG.DMRs.to.insertions.txt')
insertions.to.dmrs=NULL
for(i in 1:length(in.dmr.list)){
  temp=read.delim(in.dmr.list[i],head=F)
  insertions.to.dmrs=rbind(insertions.to.dmrs,temp)
  }

#remove the shit scaffold things with no dmrs to target
insertions.to.dmrs=subset(insertions.to.dmrs,insertions.to.dmrs$V17 != -1)
insertions.sub=subset(insertions.to.dmrs, insertions.to.dmrs$V17 < 500 & insertions.to.dmrs$V17 > -500)

close.dmrs=table(insertions.sub$V16)
count.close=rbind(count.close,close.dmrs)

rownames(count.close)=c('total insertions','insertions with CHG DMRs within 500bp')
barplot(count.close,main='Number of TE insertions with CHG DMRs within 500bp',las=2,beside=T,legend=rownames(count.close))
```

```
#proportion of insertions with DMRs within 500bp:
count.close[2,] / count.close[1,]
```

```
##   Bd1-1.bed  Bd21-3.bed   Bd3-1.bed  Bd30-1.bed BdTR12c.bed   Koz-3.bed 
##  0.08287293  0.08333333  0.17391304  0.10937500  0.08823529  0.06024096
```

```
dmr.type=table(insertions.sub$V16, insertions.sub$V15)

rownames(dmr.type)=c('Bd1-1','Bd21-3','Bd3-1','Bd30-1','BdTR12c','Koz-3')
barplot(t(dmr.type),las=2,col=c(3,4),legend=rownames(t(dmr.type)),beside=T,main='Type of CHG DMR compared to reference Bd21')
```

```
chg.count.close=count.close
```

# Insertions to CHH DMRs

1 - split DMRs to individual sample sets

```
CHH.dmrs=read.delim('CHHDMRS.all.txt',head=T)
CHH.dmrs.bed=CHH.dmrs[,c(4,5,6,2,7,1,8,3)]

dmr.type=c('Bd1-1.CHH','Bd21-3.CHH','Bd3-1.CHH','Bd30-1.CHH','BdTR12c.CHH','Koz-3.CHH')
for(i in 1:length(dmr.type)){
  temp=subset(CHH.dmrs.bed,CHH.dmrs.bed[,8]==dmr.type[i])
  write.table(temp,paste(dmr.type[i],'DMRS.bed',sep='.'),sep='\t',row.names=F,quote=F,col.names=F)

}
```

2 - Map to locaTE insertion files

```
bedtools closest -D "ref" -a insertions_Bd1-1.bed -b Bd1-1.CHH.DMRS.bed > Bd1-1.CHH.DMRs.to.insertions.txt
bedtools closest -D "ref" -a insertions_Bd21-3.bed -b Bd21-3.CHH.DMRS.bed > Bd21-3.CHH.DMRs.to.insertions.txt
bedtools closest -D "ref" -a insertions_Bd3-1.bed -b Bd3-1.CHH.DMRS.bed > Bd3-1.CHH.DMRs.to.insertions.txt
bedtools closest -D "ref" -a insertions_Bd30-1.bed -b Bd30-1.CHH.DMRS.bed > Bd30-1.CHH.DMRs.to.insertions.txt
bedtools closest -D "ref" -a insertions_BdTR12c.bed -b BdTR12c.CHH.DMRS.bed > BdTR12c.CHH.DMRs.to.insertions.txt
bedtools closest -D "ref" -a insertions_Koz-3.bed -b Koz-3.CHH.DMRS.bed > Koz-3.CHH.DMRs.to.insertions.txt
```

3 - Compare total number of insertions to those insertions with DMRs within 500bp

```
count.close=counts

in.dmr.list=dir(pattern='*CHH.DMRs.to.insertions.txt')
insertions.to.dmrs=NULL
for(i in 1:length(in.dmr.list)){
  temp=read.delim(in.dmr.list[i],head=F)
  insertions.to.dmrs=rbind(insertions.to.dmrs,temp)
  }

#remove the shit scaffold things with no dmrs to target
insertions.to.dmrs=subset(insertions.to.dmrs,insertions.to.dmrs$V17 != -1)
insertions.sub=subset(insertions.to.dmrs, insertions.to.dmrs$V17 < 500 & insertions.to.dmrs$V17 > -500)

close.dmrs=table(insertions.sub$V16)
count.close=rbind(count.close,close.dmrs)

rownames(count.close)=c('total insertions','insertions with CHH DMRs within 500bp')
barplot(count.close,main='Number of TE insertions with CHH DMRs within 500bp',las=2,beside=T,legend=rownames(count.close))
```

```
#proportion of insertions with DMRs within 500bp:
count.close[2,] / count.close[1,]
```

```
##   Bd1-1.bed  Bd21-3.bed   Bd3-1.bed  Bd30-1.bed BdTR12c.bed   Koz-3.bed 
## 0.016574586 0.000000000 0.000000000 0.015625000 0.009803922 0.000000000
```

```
dmr.type=table(insertions.sub$V16, insertions.sub$V15)

rownames(dmr.type)=c('Bd1-1','Bd21-3','Bd3-1','Bd30-1','BdTR12c','Koz-3')
barplot(t(dmr.type),las=2,col=c(3,4),legend=rownames(t(dmr.type)),beside=T,main='Type of CHH DMR compared to reference Bd21')
```

```
chh.count.close=count.close
```

# Insertion Figure

```
all.close=rbind(cg.count.close,chg.count.close[2,],chh.count.close[2,])
row.names(all.close)=c('Total TE insertions','TE insertions with CG DMR < 500bp','TE insertions with CHG DMR < 500bp','TE insertions with CHH DMR < 500bp')
barplot(all.close,main='TE Insertions',las=2,beside=T,col=c('black','#990000','#000099','#336600'),ylim=c(0,200))
```

## Deletions

0 - Get number of deletions per sample

```
in.list=dir(pattern='^deletions_*')
geno.list=matrix(unlist(strsplit(as.character(in.list),'_')),ncol=2,byrow=T)[,2]
deletions=NULL

for(i in 1:length(in.list)){
  file=read.delim(in.list[i],head=F)
  samplename=matrix(rep(geno.list[i],nrow(file)),ncol=1)
  file=cbind(file,samplename)
  deletions=rbind(deletions,file)
}

counts=table(deletions$samplename)
counts.in=counts
barplot(counts,main='number of deletions per genotype',col=1,las=2)
```

```
a=table(deletions$V1, deletions$samplename)
barplot(a[1:5,],beside=T,las=2,main='Number of deletions per chromosome')
```

# deletions to CG DMRs

1 - split DMRs to individual sample sets

```
cg.dmrs=read.delim('CGDMRS.all.txt',head=T)
cg.dmrs.bed=cg.dmrs[,c(4,5,6,2,7,1,8,3)]

dmr.type=c('Bd1-1.CG','Bd21-3.CG','Bd3-1.CG','Bd30-1.CG','BdTR12c.CG','Koz-3.CG')
for(i in 1:length(dmr.type)){
  temp=subset(cg.dmrs.bed,cg.dmrs.bed[,8]==dmr.type[i])
  write.table(temp,paste(dmr.type[i],'DMRS.bed',sep='.'),sep='\t',row.names=F,quote=F,col.names=F)

}
```

2 - Map to locaTE insertion files

```
bedtools closest -D "ref" -a deletions_Bd1-1.bed -b Bd1-1.CG.DMRS.bed > Bd1-1.CG.DMRs.to.deletions.txt
bedtools closest -D "ref" -a deletions_Bd21-3.bed -b Bd21-3.CG.DMRS.bed > Bd21-3.CG.DMRs.to.deletions.txt
bedtools closest -D "ref" -a deletions_Bd3-1.bed -b Bd3-1.CG.DMRS.bed > Bd3-1.CG.DMRs.to.deletions.txt
bedtools closest -D "ref" -a deletions_Bd30-1.bed -b Bd30-1.CG.DMRS.bed > Bd30-1.CG.DMRs.to.deletions.txt
bedtools closest -D "ref" -a deletions_BdTR12c.bed -b BdTR12c.CG.DMRS.bed > BdTR12c.CG.DMRs.to.deletions.txt
bedtools closest -D "ref" -a deletions_Koz-3.bed -b Koz-3.CG.DMRS.bed > Koz-3.CG.DMRs.to.deletions.txt
```

3 - Compare total number of deletions to those deletions with DMRs within 500bp

```
count.close=counts

in.dmr.list=dir(pattern='*CG.DMRs.to.deletions.txt')
deletions.to.dmrs=NULL
for(i in 1:length(in.dmr.list)){
  temp=read.delim(in.dmr.list[i],head=F)
  deletions.to.dmrs=rbind(deletions.to.dmrs,temp)
  }

#remove the shit scaffold things with no dmrs to target
deletions.to.dmrs=subset(deletions.to.dmrs,deletions.to.dmrs$V15 != -1)
deletions.sub=subset(deletions.to.dmrs, deletions.to.dmrs$V15 < 500 & deletions.to.dmrs$V15 > -500)

close.dmrs=table(deletions.sub$V14)
count.close=rbind(count.close,close.dmrs[1:6])

rownames(count.close)=c('total deletions','deletions with CG DMRs within 500bp')
barplot(count.close,main='Number of TE deletions with CG DMRs within 500bp',ylim=c(0,600),las=2,beside=T,legend=rownames(count.close))
```

```
#proportion of deletions with DMRs within 500bp:
count.close[2,] / count.close[1,]
```

```
##   Bd1-1.bed  Bd21-3.bed   Bd3-1.bed  Bd30-1.bed BdTR12c.bed   Koz-3.bed 
##  0.08571429  0.08207934  0.13043478  0.10940171  0.09861592  0.11976048
```

```
dmr.type=table(deletions.sub$V14, deletions.sub$V13)
dmr.type=dmr.type[,1:2]

rownames(dmr.type)=c('Bd1-1','Bd21-3','Bd3-1','Bd30-1','BdTR12c','Koz-3')
barplot(t(dmr.type),las=2,col=c(3,4),legend=rownames(t(dmr.type)),beside=T,main='Type of CG DMR compared to reference Bd21')
```

```
cg.count.close.del=count.close
```

# deletions to CHG DMRs

1 - split DMRs to individual sample sets

```
CHG.dmrs=read.delim('CHGDMRS.all.txt',head=T)
CHG.dmrs.bed=CHG.dmrs[,c(4,5,6,2,7,1,8,3)]

dmr.type=c('Bd1-1.CHG','Bd21-3.CHG','Bd3-1.CHG','Bd30-1.CHG','BdTR12c.CHG','Koz-3.CHG')
for(i in 1:length(dmr.type)){
  temp=subset(CHG.dmrs.bed,CHG.dmrs.bed[,8]==dmr.type[i])
  write.table(temp,paste(dmr.type[i],'DMRS.bed',sep='.'),sep='\t',row.names=F,quote=F,col.names=F)

}
```

2 - Map to locaTE insertion files

```
bedtools closest -D "ref" -a deletions_Bd1-1.bed -b Bd1-1.CHG.DMRS.bed > Bd1-1.CHG.DMRs.to.deletions.txt
bedtools closest -D "ref" -a deletions_Bd21-3.bed -b Bd21-3.CHG.DMRS.bed > Bd21-3.CHG.DMRs.to.deletions.txt
bedtools closest -D "ref" -a deletions_Bd3-1.bed -b Bd3-1.CHG.DMRS.bed > Bd3-1.CHG.DMRs.to.deletions.txt
bedtools closest -D "ref" -a deletions_Bd30-1.bed -b Bd30-1.CHG.DMRS.bed > Bd30-1.CHG.DMRs.to.deletions.txt
bedtools closest -D "ref" -a deletions_BdTR12c.bed -b BdTR12c.CHG.DMRS.bed > BdTR12c.CHG.DMRs.to.deletions.txt
bedtools closest -D "ref" -a deletions_Koz-3.bed -b Koz-3.CHG.DMRS.bed > Koz-3.CHG.DMRs.to.deletions.txt
```

3 - Compare total number of deletions to those deletions with DMRs within 500bp

```
count.close=counts

in.dmr.list=dir(pattern='*CHG.DMRs.to.deletions.txt')
deletions.to.dmrs=NULL
for(i in 1:length(in.dmr.list)){
  temp=read.delim(in.dmr.list[i],head=F)
  deletions.to.dmrs=rbind(deletions.to.dmrs,temp)
  }

#remove the shit scaffold things with no dmrs to target
deletions.to.dmrs=subset(deletions.to.dmrs,deletions.to.dmrs$V15 != -1)
deletions.sub=subset(deletions.to.dmrs, deletions.to.dmrs$V15 < 500 & deletions.to.dmrs$V15 > -500)

close.dmrs=table(deletions.sub$V14)
count.close=rbind(count.close,close.dmrs[1:6])

rownames(count.close)=c('total deletions','deletions with chg DMRs within 500bp')
barplot(count.close,main='Number of TE deletions with chg DMRs within 500bp',ylim=c(0,600),las=2,beside=T,legend=rownames(count.close))
```

```
#proportion of deletions with DMRs within 500bp:
count.close[2,] / count.close[1,]
```

```
##   Bd1-1.bed  Bd21-3.bed   Bd3-1.bed  Bd30-1.bed BdTR12c.bed   Koz-3.bed 
##  0.11255411  0.09575923  0.08695652  0.09059829  0.09688581  0.11177645
```

```
dmr.type=table(deletions.sub$V14, deletions.sub$V13)
dmr.type=dmr.type[,1:2]

rownames(dmr.type)=c('Bd1-1','Bd21-3','Bd3-1','Bd30-1','BdTR12c','Koz-3')
barplot(t(dmr.type),las=2,col=c(3,4),legend=rownames(t(dmr.type)),beside=T,main='Type of chg DMR compared to reference Bd21')
```

```
chg.count.close.del=count.close
```

# deletions to CHH DMRs

1 - split DMRs to individual sample sets

```
CHH.dmrs=read.delim('CHHDMRS.all.txt',head=T)
CHH.dmrs.bed=CHH.dmrs[,c(4,5,6,2,7,1,8,3)]

dmr.type=c('Bd1-1.CHH','Bd21-3.CHH','Bd3-1.CHH','Bd30-1.CHH','BdTR12c.CHH','Koz-3.CHH')
for(i in 1:length(dmr.type)){
  temp=subset(CHH.dmrs.bed,CHH.dmrs.bed[,8]==dmr.type[i])
  write.table(temp,paste(dmr.type[i],'DMRS.bed',sep='.'),sep='\t',row.names=F,quote=F,col.names=F)

}
```

2 - Map to locaTE insertion files

```
bedtools closest -D "ref" -a deletions_Bd1-1.bed -b Bd1-1.CHH.DMRS.bed > Bd1-1.CHH.DMRs.to.deletions.txt
bedtools closest -D "ref" -a deletions_Bd21-3.bed -b Bd21-3.CHH.DMRS.bed > Bd21-3.CHH.DMRs.to.deletions.txt
bedtools closest -D "ref" -a deletions_Bd3-1.bed -b Bd3-1.CHH.DMRS.bed > Bd3-1.CHH.DMRs.to.deletions.txt
bedtools closest -D "ref" -a deletions_Bd30-1.bed -b Bd30-1.CHH.DMRS.bed > Bd30-1.CHH.DMRs.to.deletions.txt
bedtools closest -D "ref" -a deletions_BdTR12c.bed -b BdTR12c.CHH.DMRS.bed > BdTR12c.CHH.DMRs.to.deletions.txt
bedtools closest -D "ref" -a deletions_Koz-3.bed -b Koz-3.CHH.DMRS.bed > Koz-3.CHH.DMRs.to.deletions.txt
```

3 - Compare total number of deletions to those deletions with DMRs within 500bp

```
count.close=counts

in.dmr.list=dir(pattern='*CHH.DMRs.to.deletions.txt')
deletions.to.dmrs=NULL
for(i in 1:length(in.dmr.list)){
  temp=read.delim(in.dmr.list[i],head=F)
  deletions.to.dmrs=rbind(deletions.to.dmrs,temp)
  }

#remove the shit scaffold things with no dmrs to target
deletions.to.dmrs=subset(deletions.to.dmrs,deletions.to.dmrs$V15 != -1)
deletions.sub=subset(deletions.to.dmrs, deletions.to.dmrs$V15 < 500 & deletions.to.dmrs$V15 > -500)

close.dmrs=table(deletions.sub$V14)
count.close=rbind(count.close,close.dmrs[1:6])

rownames(count.close)=c('total deletions','deletions with chh DMRs within 500bp')
barplot(count.close,main='Number of TE deletions with chh DMRs within 500bp',ylim=c(0,600),las=2,beside=T,legend=rownames(count.close))
```

```
#proportion of deletions with DMRs within 500bp:
count.close[2,] / count.close[1,]
```

```
##   Bd1-1.bed  Bd21-3.bed   Bd3-1.bed  Bd30-1.bed BdTR12c.bed   Koz-3.bed 
##  0.02034632  0.01094391  0.01086957  0.01196581  0.02249135  0.01796407
```

```
dmr.type=table(deletions.sub$V14, deletions.sub$V13)
dmr.type=dmr.type[,1:2]

rownames(dmr.type)=c('Bd1-1','Bd21-3','Bd3-1','Bd30-1','BdTR12c','Koz-3')
barplot(t(dmr.type),las=2,col=c(3,4),legend=rownames(t(dmr.type)),beside=T,main='Type of chh DMR compared to reference Bd21')
```

```
chh.count.close.del=count.close
```

# Deletion Figure

```
all.close.del=rbind(cg.count.close.del,chg.count.close.del[2,],chh.count.close.del[2,])
row.names(all.close.del)=c('Total TE deletions','TE deletions with CG DMR < 500bp','TE deletions with CHG DMR < 500bp','TE deletions with CHH DMR < 500bp')
barplot(all.close.del,main='TE deletions',las=2,beside=T,col=c('black','#990000','#000099','#336600'),ylim=c(2500,0))
```
