# Supplementary figures and images for: DNA methylation profiles of diverse Brachypodium distachyon align with underlying genetic diversity
Source: Genome Res. 2016 Nov;26(11):1520–31. doi: 10.1101/gr.205468.116 (PMC5088594; doi:10.1101/gr.205468.116)

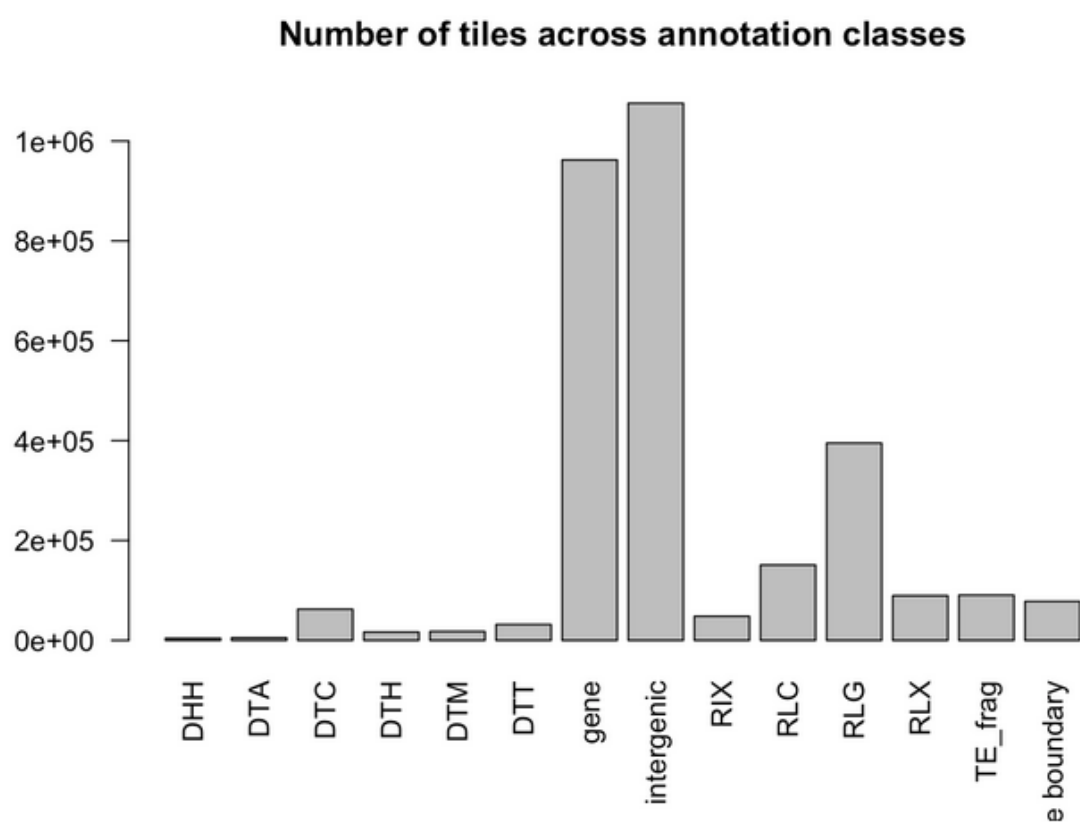

**Supplemental Figure 3:** Number of genomic tiles intersecting with annotation features

Supplement: Supplemental Material [file supp_gr.205468.116_Supplemental_Fig_S3.pdf]

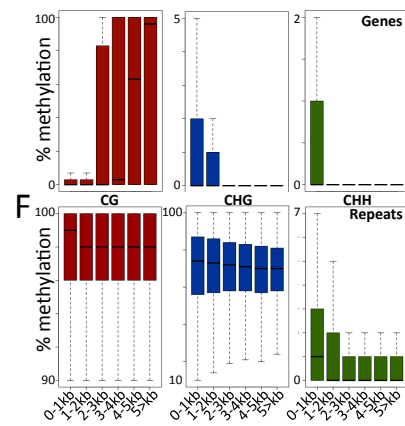

**Supplemental Figure 8:** DNA methylation for genes (top) and TEs (bottom) divided by element size

Supplement: Supplemental Material [file supp_gr.205468.116_Supplemental_Fig_S8.pdf]

## Bd21-3

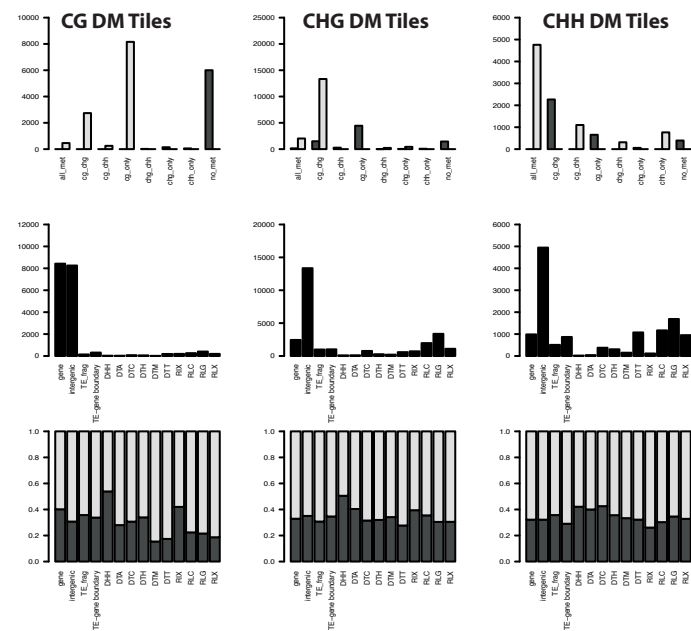

## Bd3-1

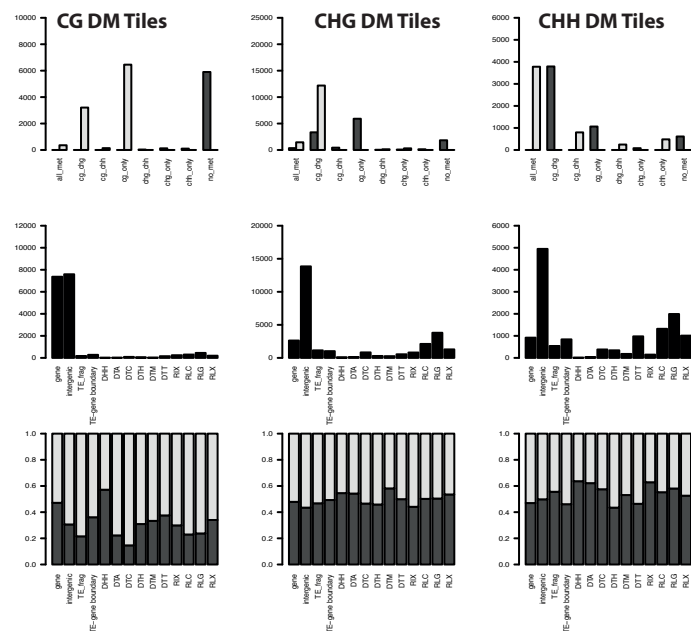

## Bd30-1

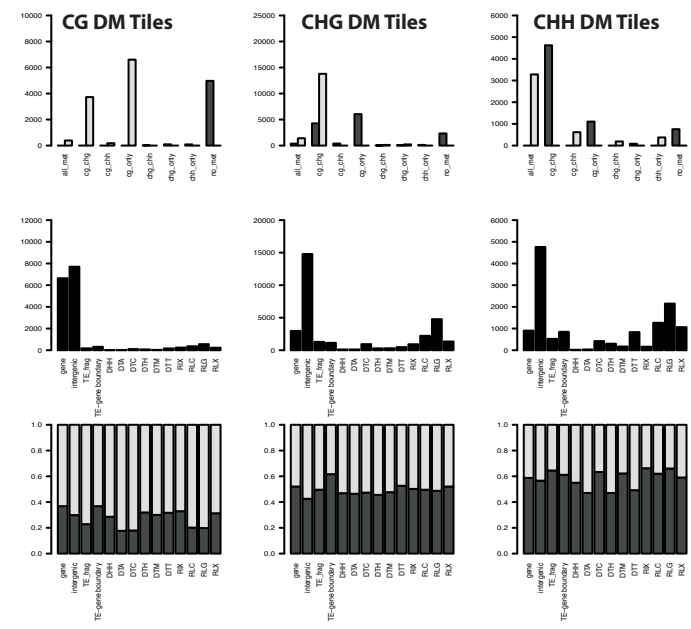

## Koz-3

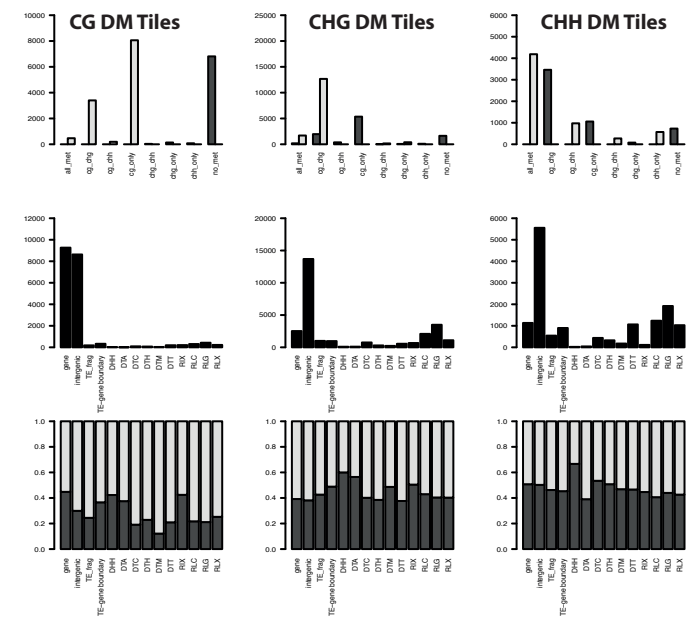

## BdTR12c

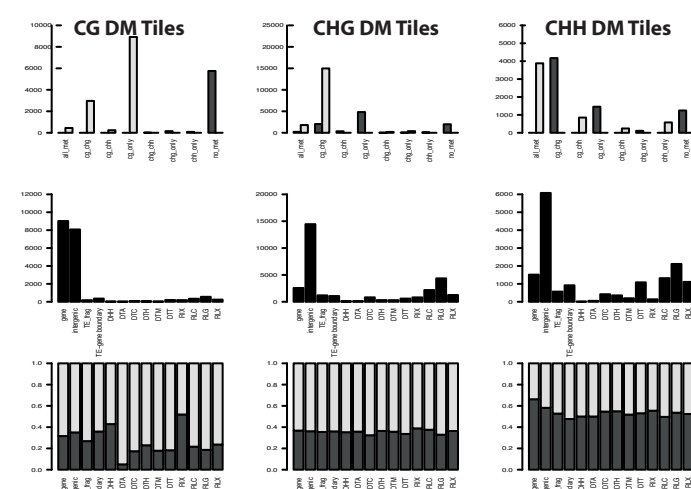

**Supplemental Figure 12:** DM Tile analysis for inbreds Bd21-3, Bd3-1, Bd30-1, Koz-3, and BdTR12c.

Supplement: Supplemental Material [file supp_gr.205468.116_Supplemental_Fig_S12.pdf]

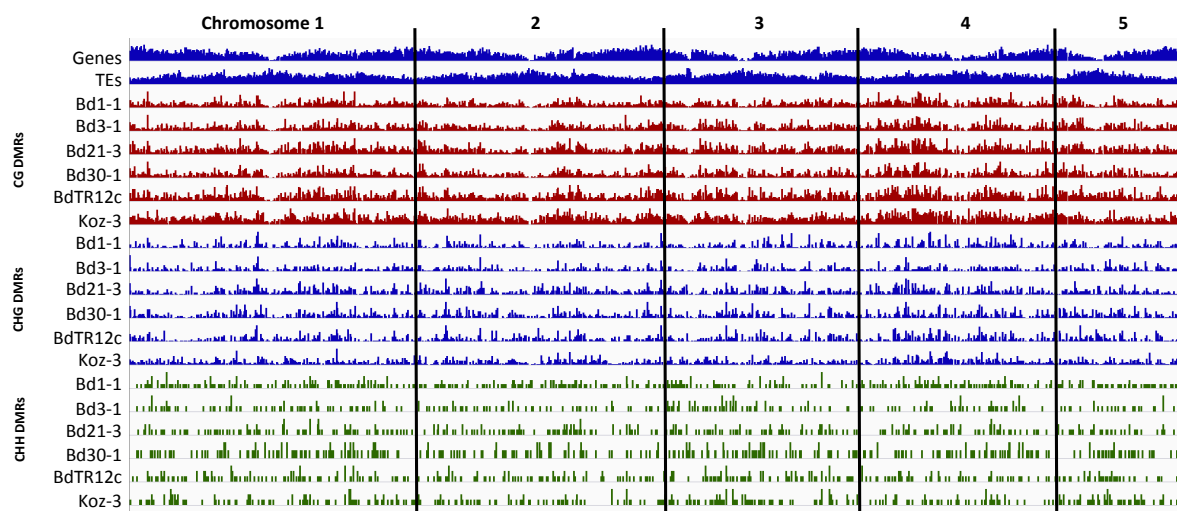

**Supplemental Figure 13.** Position of DMRs across all lines and chromosomes

Supplement: Supplemental Material [file supp_gr.205468.116_Supplemental_Fig_S13.pdf]

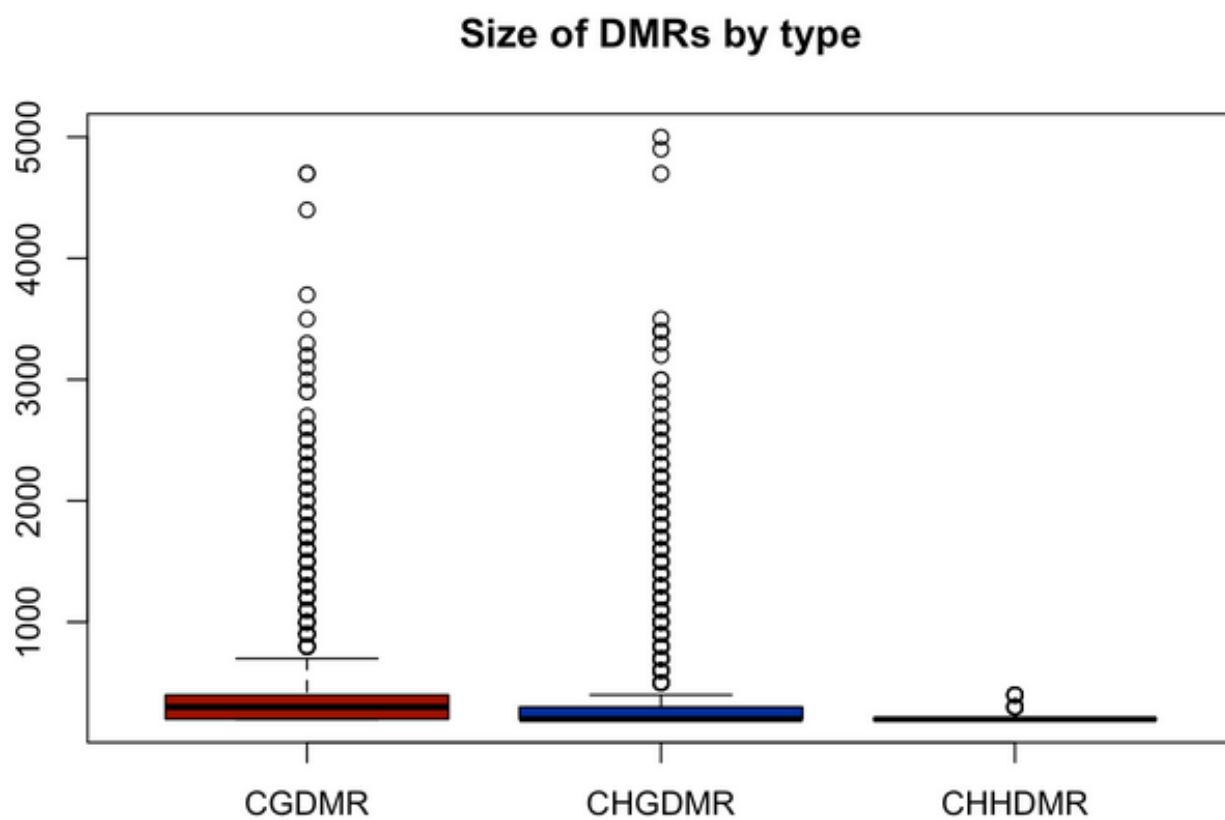

**Supplemental Figure 14.** Boxplot of DMR sizes per sequence context

Supplement: Supplemental Material [file supp_gr.205468.116_Supplemental_Fig_S14.pdf]

CG

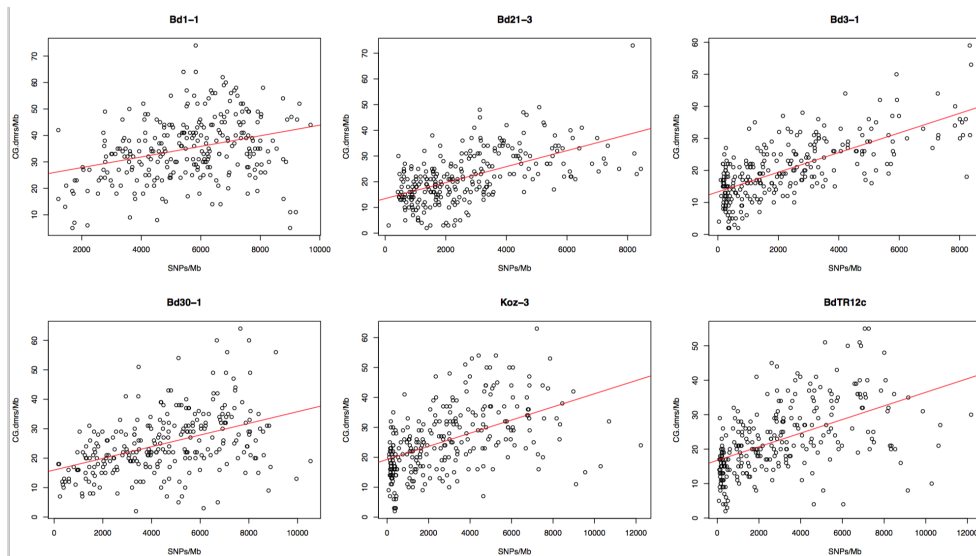

CHG

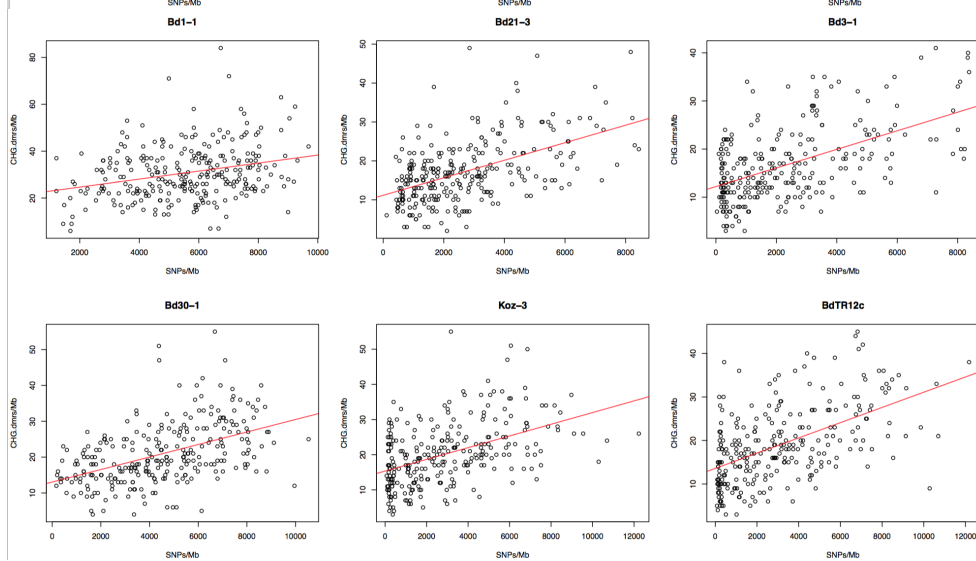

CHH

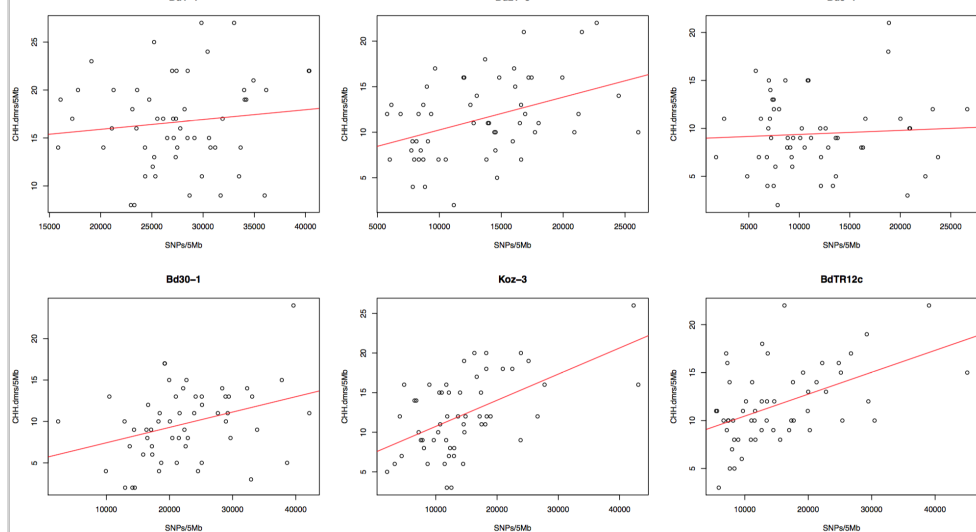

Supplemental Figure 22: Correlation plots for all DMR types for all individual samples

Supplement: Supplemental Material [file supp_gr.205468.116_Supplemental_Fig_S22.pdf]
