# Supplementary material for: DNA methylation profiles of diverse Brachypodium distachyon align with underlying genetic diversity
Source: Genome Res. 2016 Nov;26(11):1520–31. doi: 10.1101/gr.205468.116 (PMC5088594; doi:10.1101/gr.205468.116)
Supplement: Supplemental Material [file supp_gr.205468.116_Supplemental_Fig_S9.pdf]

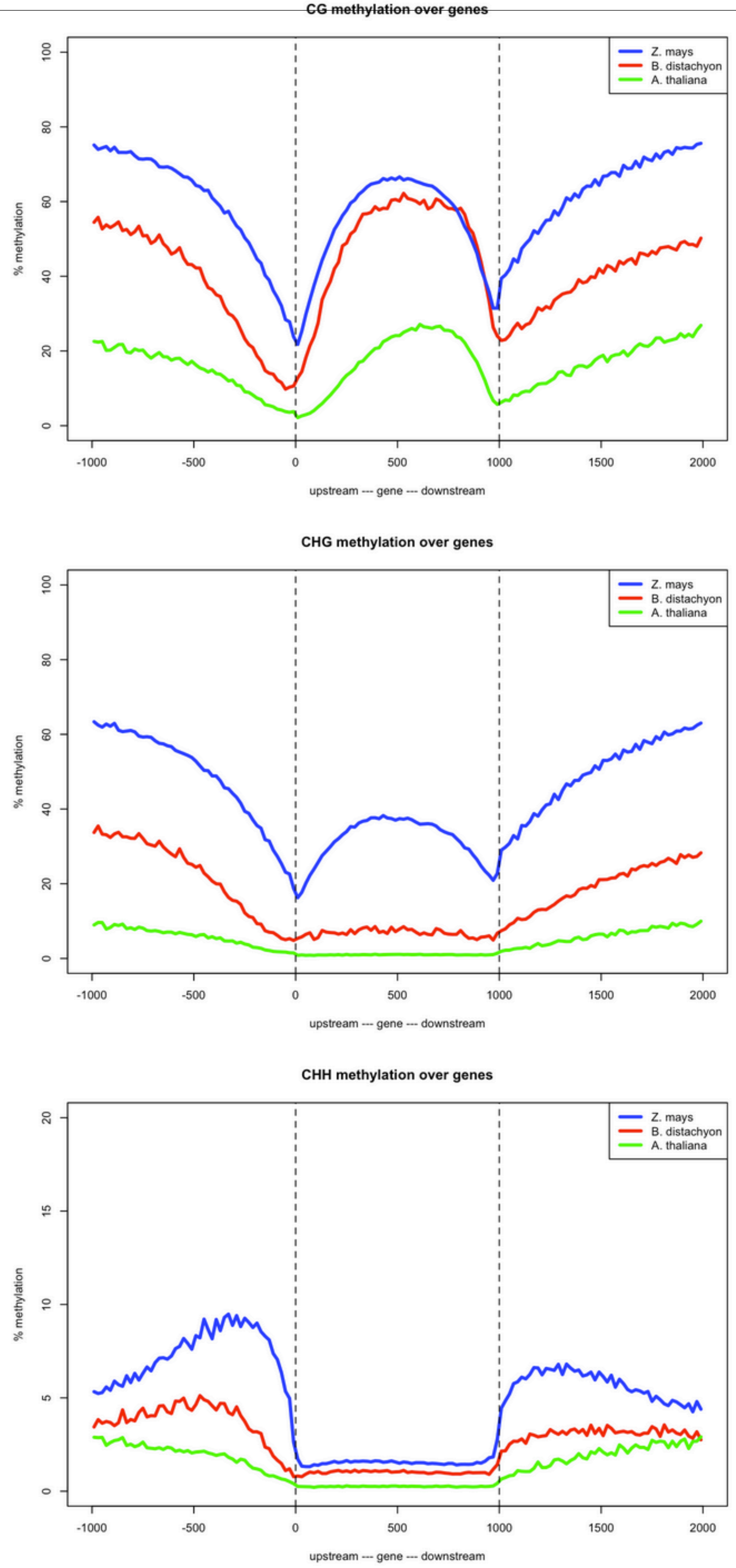

Supplemental Figure 9: Methylation comparisons of B73 (Z. mays), Col-0 (A. thaliana), and Bd21 (B. distachyon) over gene models. Plots are colored by species and split by methylation sequence context. Note scale for CHH methylation plot
