# Supplementary material for: DNA methylation profiles of diverse Brachypodium distachyon align with underlying genetic diversity
Source: Genome Res. 2016 Nov;26(11):1520–31. doi: 10.1101/gr.205468.116 (PMC5088594; doi:10.1101/gr.205468.116)
Supplement: Supplemental Material [file supp_gr.205468.116_Supplemental_Fig_S15.pdf]

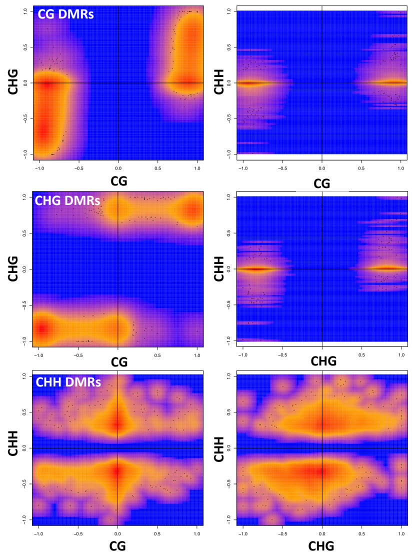

Supplemental Figure 15. Scatter plots of methylation states across CG, CHG, and CHH DMR regions. Red indicates increased point cloud density.
