# Supplementary material for: DNA methylation profiles of diverse Brachypodium distachyon align with underlying genetic diversity
Source: Genome Res. 2016 Nov;26(11):1520–31. doi: 10.1101/gr.205468.116 (PMC5088594; doi:10.1101/gr.205468.116)
Supplement: Supplemental Material [file supp_gr.205468.116_Supplemental_Fig_S17.pdf]

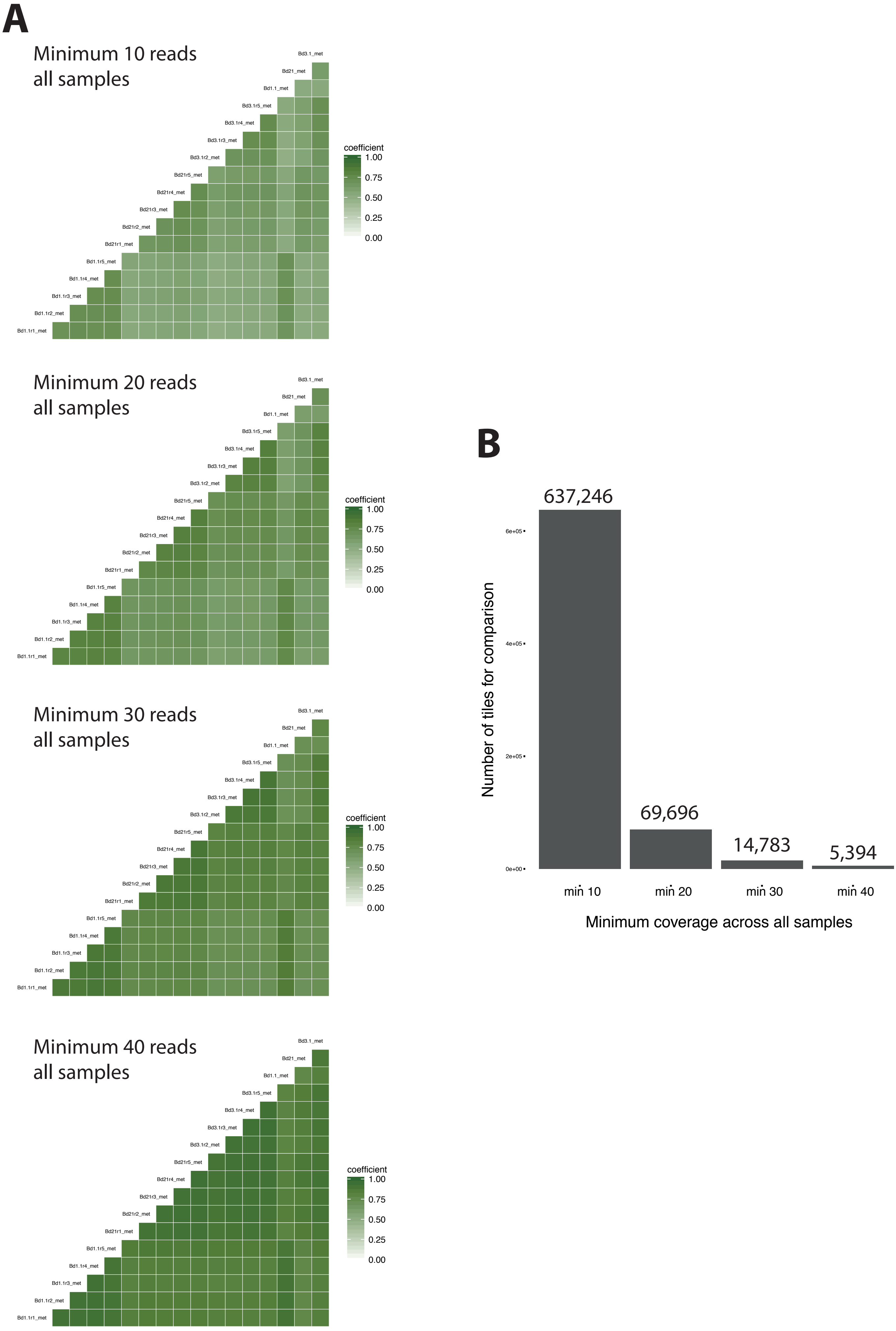

Supplemental Figure 17: (A) Correlation heatmaps for CHH sites requiring various minimum read depth. (B) Barplot of number of sites available for analysis given minimum read depth filtering.
