# Supplementary material for: DNA methylation profiles of diverse Brachypodium distachyon align with underlying genetic diversity
Source: Genome Res. 2016 Nov;26(11):1520–31. doi: 10.1101/gr.205468.116 (PMC5088594; doi:10.1101/gr.205468.116)
Supplement: Supplemental Material [file supp_gr.205468.116_Supplemental_Fig_S18.pdf]

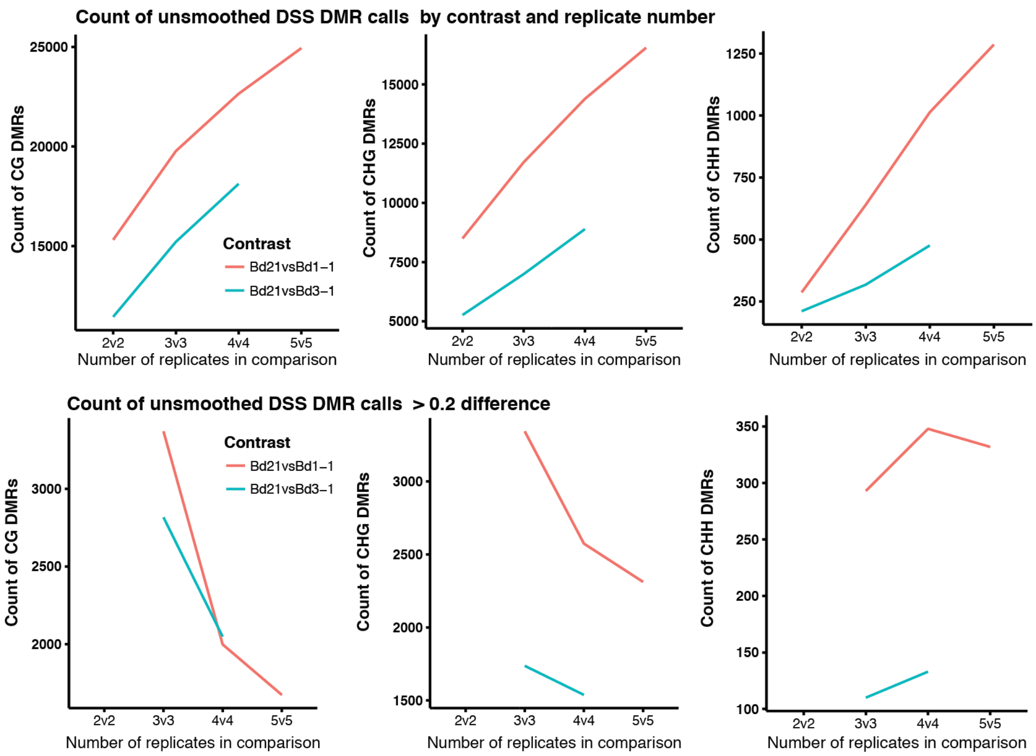

Supplemental Figure 18. Comparisons of unsmoothed DSS results for 2v2, 3v3, 4v4, and 5v5 (Bd1-1 only) replicate comparisons. Plots indicate total number of DMRs called (top panels) and the number of DMRs with differences between accessions > 0.2 (bottom panels).
