# Supplementary material for: DNA methylation profiles of diverse Brachypodium distachyon align with underlying genetic diversity
Source: Genome Res. 2016 Nov;26(11):1520–31. doi: 10.1101/gr.205468.116 (PMC5088594; doi:10.1101/gr.205468.116)
Supplement: Supplemental Material [file supp_gr.205468.116_Supplemental_Fig_S19.pdf]

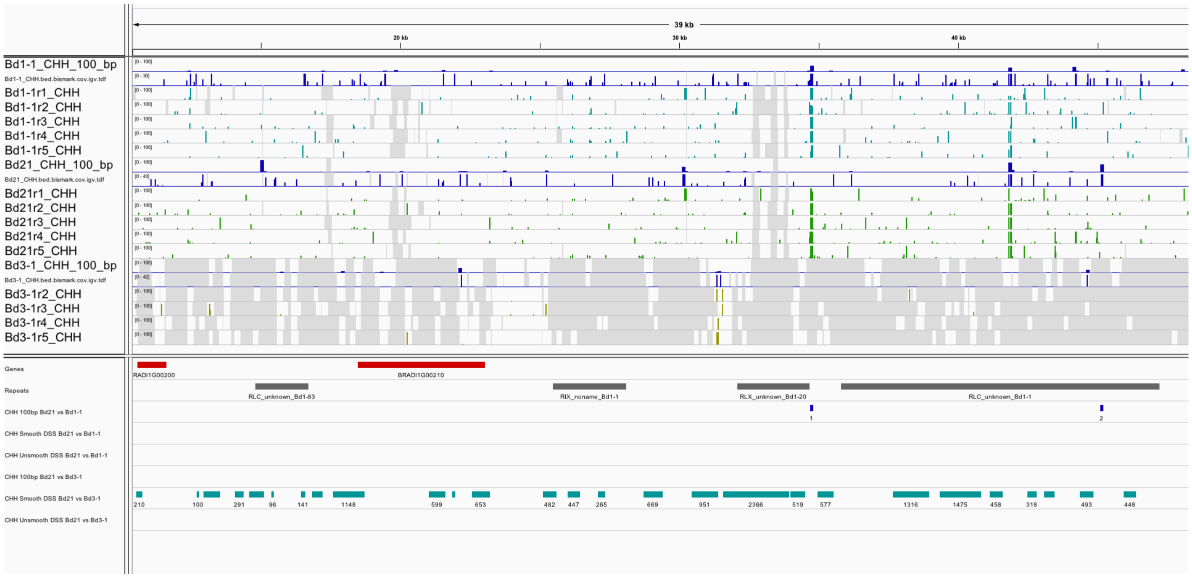

Supplemental Figure 19. Genomic view of Bd1:10,391-49,958 highlighting smooth DSS DMRs being called across a largely absent region of Bd3-1. Aqua bars indicate DMR calls.
