# Supplementary material for: DNA methylation profiles of diverse Brachypodium distachyon align with underlying genetic diversity
Source: Genome Res. 2016 Nov;26(11):1520–31. doi: 10.1101/gr.205468.116 (PMC5088594; doi:10.1101/gr.205468.116)
Supplement: Supplemental Material [file supp_gr.205468.116_Supplemental_Fig_S20.pdf]

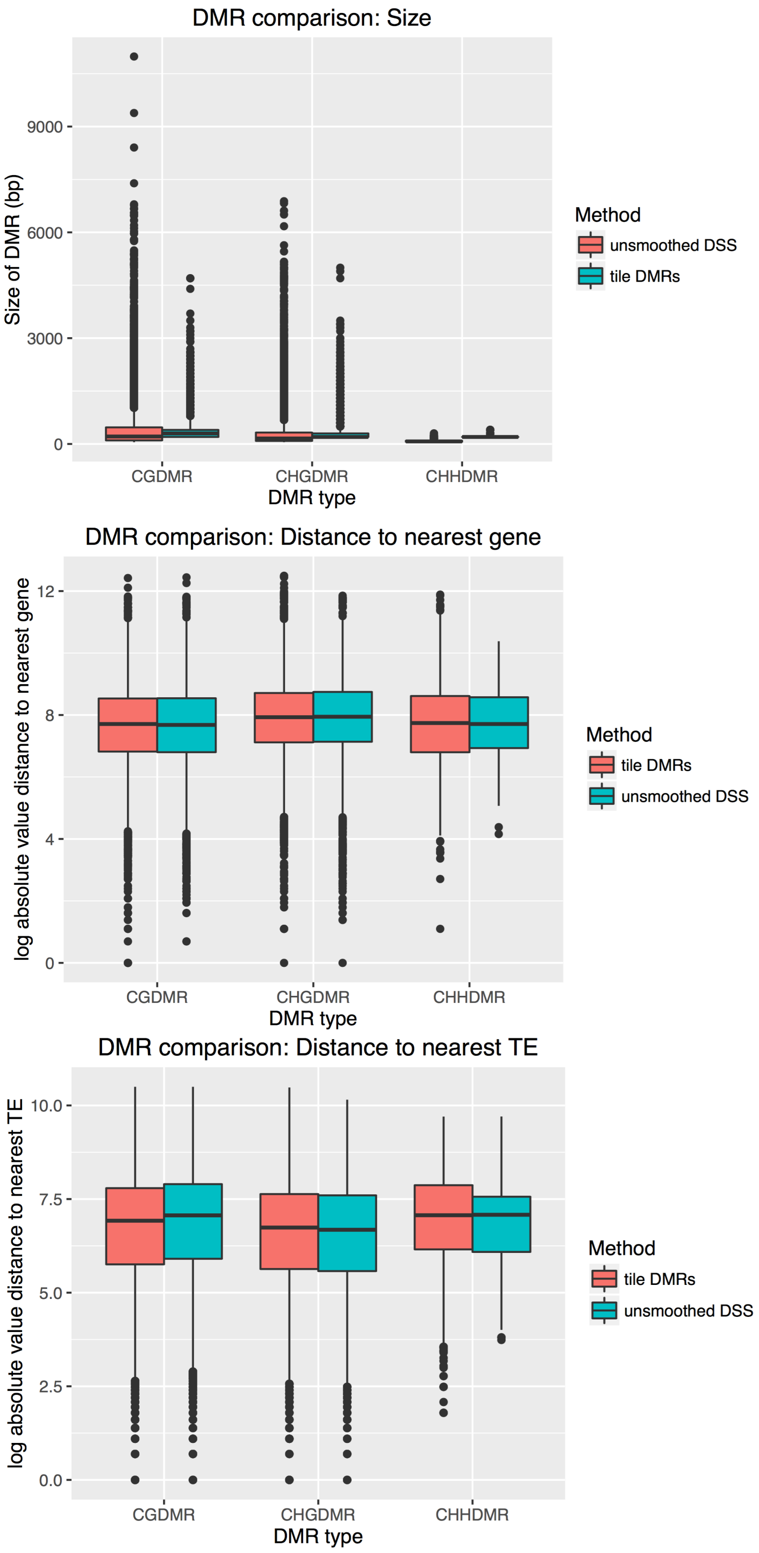

Supplemental Figure 20. Boxplots of size (top), log distance to nearest annotated gene (center), and log distance to nearest annotated te (bottom) for 100bp tile DMRs compared to unsmoothed DSS DMRs.
