# Supplementary material for: DNA methylation profiles of diverse Brachypodium distachyon align with underlying genetic diversity
Source: Genome Res. 2016 Nov;26(11):1520–31. doi: 10.1101/gr.205468.116 (PMC5088594; doi:10.1101/gr.205468.116)
Supplement: Supplemental Material [file supp_gr.205468.116_Supplemental_Fig_S26.pdf]

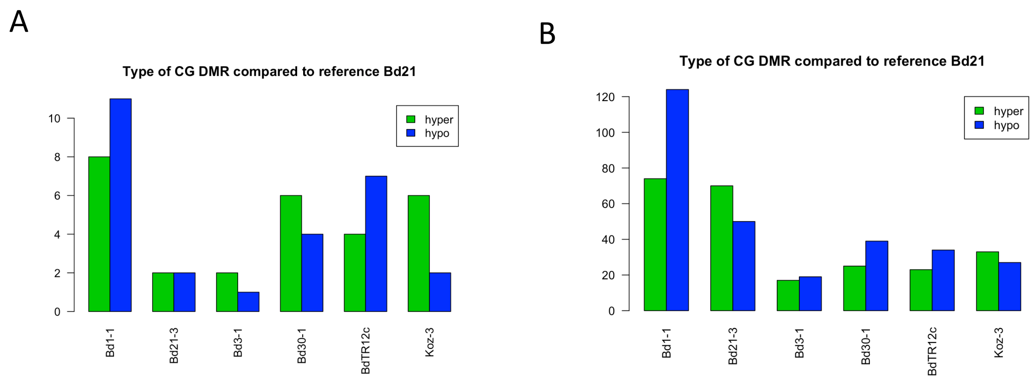

Supplemental Figure 26. Barplots indicating the DMR state (hyper or hypomethylated) compared to the Bd21 reference methylation state for DMRs within 500bp of (A) transposable element insertions and (B) deletions
