# Supplementary material for: DNA methylation profiles of diverse Brachypodium distachyon align with underlying genetic diversity
Source: Genome Res. 2016 Nov;26(11):1520–31. doi: 10.1101/gr.205468.116 (PMC5088594; doi:10.1101/gr.205468.116)
Supplement: Supplemental Material [file supp_gr.205468.116_Supplemental_Legends.docx]

**Supplemental Fig S1**: Density distributions describing CG, CHG, and CHH methylation Brachypodium.

**Supplemental Fig S2:** Number of genomic tiles falling into eight possible DNA methylation classes in Bd21

**Supplemental Fig S3**: Number of genomic tiles intersecting with annotation features

**Supplemental Fig S4:** Relative distance plots of average methylation for ten *B. distachyon* annotated transposon superfamilies. Methylation is shown in CG (red), CHG (blue), and CHH (green) contexts.

**Supplemental Fig S5**: Density distribution (A) and median distance (B) of each annotated transposon superfamily to its nearest gene. (C) Element size (in bp) boxplot by transposon annotated superfamily.

**Supplemental Fig S6:** Sum of 24nt RNAs from IBI 2010 normalized by total element size sum by transposon superfamily.

**Supplemental Fig S7:** Relative methylation of annotated Bd21 genes with intronic gene sequences removed. CG gene-body methylation is maintained without intronic sequence methylation levels.

**Supplemental Fig S8**: DNA methylation for genes (top) and TEs (bottom) divided by element size

**Supplemental Fig S9:** Methylation comparisons of B73 (*Z. mays*), Col-0 (*A. thaliana*), and Bd21 (*B. distachyon*) over gene models. Plots are colored by species and split by methylation sequence context. Note scale for CHH methylation plot

**Supplemental Fig S10:** Proportion of methylated tiles across Bd21 chromosomes. Percentages defining methylated are provided

**Supplemental Fig S11**: Relative methylation across genes and repeats for all seven inbreds. Plots have been split by methylation context.

**Supplemental Fig S12**: DM Tile analysis for inbreds Bd21-3, Bd3-1, Bd30-1, Koz-3, and BdTR12c

**Supplemental Fig S13**: Position of DMRs across all lines and chromosomes

**Supplemental Fig S14**: Boxplot of DMR sizes per sequence context

**Supplemental Fig S15**: Scatter plots of methylation states across CG, CHG, and CHH DMR regions. Red indicates increased point cloud density.

**Supplemental Fig S16:** CHH methylation correlation plots for biological replicates for CHH levels > 30% in at least one (center) or all (right) samples.

**Supplemental Fig S17:** (A) Correlation heatmaps for CHH sites requiring various minimum read depth. (B) Barplot of number of tiles available for analysis given minimum read depth filtering.

**Supplemental Fig S18:** Comparisons of unsmoothed DSS results for 2v2, 3v3, 4v4, and 5v5 (Bd1-1 only) replicate comparisons. Plots indicate total number of DMRs called (top panels) and the number of DMRs with differences between accessions > 0.2 (bottom panels).

**Supplemental Fig S19**: Genomic view of Bd1:10,391-49,958 highlighting smooth DSS DMRs being called across a largely absent region of Bd3-1. Aqua bars indicate DMR calls.

**Supplemental Fig S20:** Boxplots of size (top), log distance to nearest annotated gene (center), and log distance to nearest annotated te (bottom) for 100bp tile DMRs compared to unsmoothed DSS DMRs.

**Supplemental Fig S21:** Density distribution of methylation differences between accession groups in 100bp tile DMRs. Vertical bars indicate fixed cutoff for CG (red) and CHG (blue) DMRs.

**Supplemental Fig S22:** Correlation plots for all DMR types for all individual samples

**Supplemental Fig S23**: Correlation of DSS DMRs defined from biological replicates to SNP density across methylation contexts

**Supplemental Fig S24**: Barplots indicating the number of genotypes individual transposable elements are inserted (A) or deleted (B) across samples.

**Supplemental Fig S25**: TEs inserted into multiple genotypes and/or multiple times in a single sample

**Supplemental Fig S26**: Barplots indicating the DMR state (hyper or hypomethylated) compared to the Bd21 reference methylation state for DMRs within 500bp of (A) transposable element insertions and (B) deletions

**Supplemental Table 1**: Bisulfite sequencing summary statistics

**Supplemental Table 2**: List of CG/CHG Differentially Methylated Regions

**Supplemental Table 3**: List of CHH Differentially Methylated Regions

**Supplemental Table 4**: Bisulfite sequencing summary statistics for biological replicate data

**Supplemental Table 5**: List of Smooth DSS DMRs

**Supplemental Table 6**: List of Unsmooth DSS DMRs

**Supplemental Table 7**: Transposable element polymorphisms in *Brachypodium distachyon*

**Supplemental Table 8**: Bisulfite sequencing summary statistics for other plant species

**Supplemental Data 1:** Analysis scripts for sequence alignment and figure development
